# Fluorescence Lifetime Imaging Techniques—A Review on Principles, Applications and Clinical Relevance

**DOI:** 10.1002/jbio.202400450

**Published:** 2025-02-19

**Authors:** V. I. Shcheslavskiy, M. V. Shirmanova, K. S. Yashin, A. C. Rück, M. C. Skala, W. Becker

**Affiliations:** ^1^ Privolzhsky Research Medical University Nizhny Novgorod Russia; ^2^ Becker&Hickl GmbH Berlin Germany; ^3^ Centre for Biomedical Research, Microscopy/Neurology Group University Ulm Ulm Germany; ^4^ Morgridge Institute for Research, Department of Biomedical Engineering University of Wisconsin Madison Wisconsin USA

**Keywords:** fluorescence lifetime imaging, metabolic imaging, optical biopsy, phosphorescence lifetime imaging, time‐correlated single photon counting

## Abstract

This article gives an overview of the most frequently used fluorescence‐lifetime imaging (FLIM) techniques, their capabilities, and typical applications. Starting from a general introduction to fluorescence and phosphorescence lifetime, we will show that the fluorescence lifetime or, more accurately, the fluorescence decay function of a fluorophore is a direct indicator of the interaction with its molecular environment. FLIM is therefore more than a simple contrast technique in microscopy—it is a technique of molecular imaging. FLIM techniques can be classified into time‐domain and frequency‐domain techniques, analogue and photon counting techniques, and scanning and wide‐field techniques. Starting from an overview of these general technical principles we will describe the features and peculiarities of the different FLIM techniques in use. An extended section is dedicated to TCSPC FLIM, addressing unique capabilities that make the technique especially interesting to FLIM of biological systems.

## Graphical abstract

This article gives an overview of the most frequently used fluorescence‐lifetime imaging (FLIM) techniques, their capabilities and typical applications in biology and clinical studies.

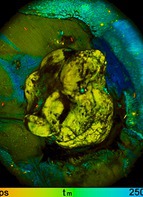

## 1 Introduction

Fluorescence techniques have found broad application in life sciences because they are non‐invasive, non‐destructive, extremely sensitive and able to deliver information about biochemical interactions on the molecular scale. Fluorescence techniques have benefited from the development of highly sensitive semiconductor‐based CCD and CMOS imaging sensors and, especially, confocal and multiphoton laser scanning microscopes. Semiconductor chips allow images to be recorded within extremely short acquisition times, whereas laser scanning microscopes record images from exactly defined focal planes within a sample. Another advantage of laser scanning systems is that the recording process can be made multi‐dimensional. Data are obtained over three spatial dimensions, the emission and excitation wavelength, the experiment time and the polarisation of the light.

The combination of fluorescence imaging with fluorescence‐decay detection adds additional dimensions to the recording process. Unlike the fluorescence intensity, the fluorescence lifetime generally does not depend on the concentration of the fluorophore. In the simplest case, the fluorescence lifetime can be used as a parameter to separate or identify the emission of different fluorophores [1, 2, 3]. More importantly, the parameters of the fluorescence decay function are direct indicators of molecular states in biological systems. FLIM data contain information on the concentration of biologically relevant ions, pH, protein interaction and protein structure or the metabolic state of cells and tissues.

Different technical principles of fluorescence‐decay recording and different combinations with imaging techniques are in use. There are wide‐field techniques, scanning techniques, frequency‐ and time‐domain techniques, digital (photon counting) techniques and analogue techniques. The techniques differ in time resolution, ability to resolve multi‐exponential decay functions into exponential components, sensitivity, photon efficiency, intensity range they work best, recording speed and, importantly, capability to record several biologically relevant parameters simultaneously and their mutual dependence.

The authors admit that they are strong fanciers of TCSPC FLIM. They have good reasons for that. TCSPC FLIM is a combination of the most sensitive and accurate techniques of spatial resolution and temporal resolution, and it addresses the complex needs of molecular imaging. Additionally, TCSPC instrumentation is well documented with technical details, best practices for analysis, and statistical considerations for interpretation [4, 5].

The authors of this article are under the impression that the relationship between spatial resolution, temporal resolution, multi‐parameter recording, recording speed and applications of different FLIM techniques is not commonly understood in its full complexity. This article is an attempt to help FLIM users bridge the gap between the technical principles of FLIM and applications in life sciences.

Starting from an overview of the general technical principles, we will describe the features and peculiarities of the different FLIM techniques in use. An extended section is dedicated to TCSPC FLIM, addressing unique capabilities that make the technique especially interesting to FLIM of biological systems. This section contains multi‐wavelength FLIM, excitation‐wavelength multiplexing, accumulation of fast time‐series of dynamic fluorescence lifetime effects, simultaneous FLIM/PLIM and TCSPC FLIM with different scanning techniques.

A short section considers non‐ideal effects of FLIM, time resolution and the parameters describing it, and statistical accuracy of FLIM data.

A section on FLIM data analysis covers frequency‐domain (Phasor) analysis and time‐domain analysis, including data processing principles.

A section on standard molecular‐imaging applications covers measurement of molecular‐environment parameters, measurement of fast dynamic effects, membrane potential measurement and FRET measurement. Special room is given to metabolic imaging by NADH and FAD FLIM, and the metabolic indicators are derived from the corresponding decay parameters.

The last part of the article describes clinical and preclinical applications of FLIM. It covers FLIM Histology, personalised Chemotherapy, T cells and macrophages, stem cells, multiphoton tomography of skin, malignant melanoma, ophthalmic FLIM, metabolic FLIM of macroscopic objects, FLIM endoscopy, FLIM assisted surgery and optical biopsy.

### 1.1 Fluorescence

**FIGURE 1:**
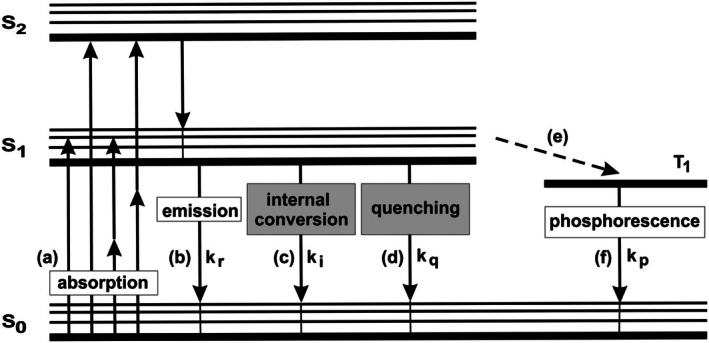
Processes involved in the excitation and emission of fluorescence including (a) absorption, (b) fluorescent emission, (c) internal conversion, (d) quenching, (e) transit to the triplet state and (f) phosphorescence emission.

Processes involved in the excitation and emission of fluorescence and phosphorescence are illustrated in Figure 1. When a molecule absorbs a photon it enters an excited state (a). From there it can return by emitting a photon of longer wavelength (b), by internally converting the energy into heat (c) or by transferring the energy to another molecule (d). This can be a solvent molecule, another light‐absorbing molecule or an electron donor or acceptor with which the excited molecule exchanges an electron. The average time the molecule stays in the excited state is the excited‐state lifetime. The absorption/emission process is called fluorescence, the temporal profile of the emission is the fluorescence decay function, and the 1/*e* time of the fluorescence decay function is the fluorescence lifetime.

A molecule can also be excited by two or more photons arriving simultaneously [6, 7]. Two‐photon or, in the general case, multi‐photon excitation requires extremely high power density. However, when femtosecond laser pulses are focused by a high‐NA microscope lens the required power density is easily reached. Multiphoton excitation is therefore a standard technique in laser scanning microscopy.

Instead of taking one of the relaxation paths, b, c, and d, an excited molecule can also transit to the triplet state (e), see Figure 1, right. From there, it can transit to the ground state by emitting phosphorescence (f). The transition from T1 to S0 is a ‘forbidden’ process, therefore phosphorescence lifetimes can be very long. Phosphorescence is extremely weak for organic molecules but it can be the dominating decay path for specially designed phosphorescence markers.

The probability that the molecule returns from the excited state is time‐invariant, that is, it does not depend on the time the molecule has already spent in the excited state. When a homogeneous assembly of molecules is excited with a short pulse of light the temporal profile of the emission is therefore a single exponential.

### 1.2 The Fluorescence Decay Function as an Indicator of Molecular Interactions

**FIGURE 2:**
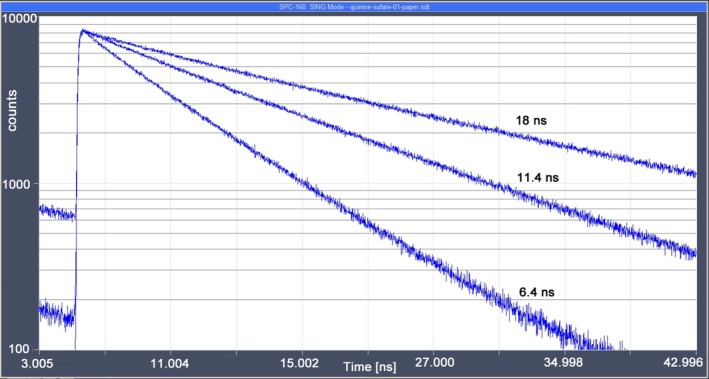
Dependence of the fluorescence lifetime of quinine sulphate on the Cl^−^ concentration. The lifetime decreases from 18 ns at low Cl^−^ to 6.4 ns at high Cl^−^.

As can be seen from Figure 1, the effective decay rate of the fluorescence is the sum of the decay rates of the radiative decay, *k*_r_ and the non‐radiative decay rates, *k*_i_ and *k*_ext_, of internal conversion and energy transition to the molecular environment. Important is that the radiative decay rate and the rate of internal conversion are properties of the molecule, but k_ext_ depends on the molecular environment. The fluorescence lifetime (the reciprocal sum of the decay rates) is therefore environment‐dependent. It can be used as an indicator of the molecular environment of the molecule. The classic example is the dependence of the fluorescence lifetime of Quinine sulphate on the concentration of chloride ions, see Figure 2. However, there are environment‐dependent lifetime changes for almost all fluorophores. Even the green fluorescent protein (GFP) which is often presumed to be environment‐invariant shows detectable changes [8, 9].

The molecular process of the lifetime change of quinine is ‘collisional quenching’: The excited quinine molecule collides with a Cl^−^ which takes away the energy, leaving the quinine molecule in the ground state. The concentration of Cl^−^ can be directly calculated from the lifetime change.

**FIGURE 3:**
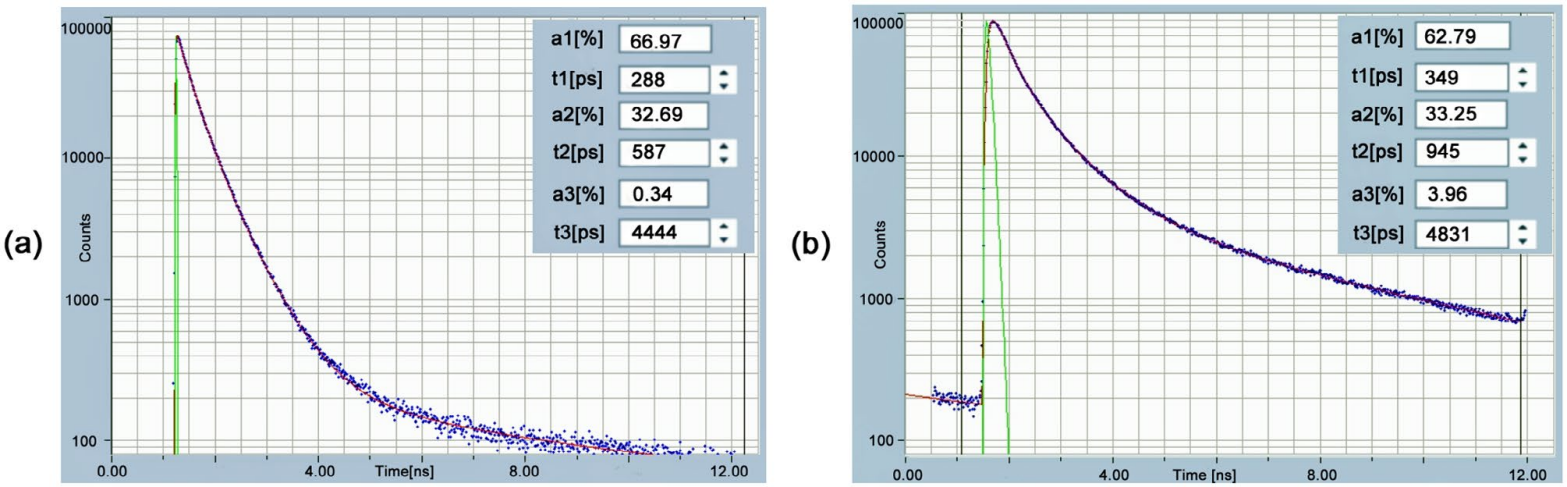
Multi‐exponential composition of decay curves of NAD(P)H. (a) NAD(P)H in water. (b) NAD(P)H in a live cell. The instrument response function (IRF, see Section 2.5.1) is in green, data points in blue, and best‐fit line in red. IRF width is different due to different detectors, 22 ps for (a) and 100 ps for (b). Triple‐exponential analysis, amplitudes, *a*_1_–*a*_3_, and decay times, *t*_1_, *t*_2_, *t*_3_ of the decay components listed in the inserts. Time axis is from 0 to 12 ns.

In the case of collisional quenching the shape of the fluorescence decay curve remains single exponential. However, in biological applications, single‐exponential decay functions are rather the exception than the rule. In the simplest case, the environment of the molecules may be inhomogeneous. In other cases, a part of the fluorophore population is bound to proteins. Bound and unbound molecules have different non‐radiative decay rates and thus different lifetimes. It also happens that the fluorophore interacts with an absorber via Förster‐Resonance Energy Transfer (FRET) [10, 11]. Also here, only a part of the molecules may interact, causing a double‐exponential decay. There are many other examples. In each case, the biological information is not primarily in an average fluorescence lifetime but in the lifetimes (*t*_1_, *t*_2_, …) and amplitudes (*a*_1_, *a*_2_, …) of the individual decay components [4]. An example is shown in Figure 3. It shows fluorescence decay curves of NAD(P)H in water and in a live cell. Even visually, the shapes of the curves are different. Detailed analysis shows that there are at least three decay components. It is the task of FLIM data analysis to split the decay curves into their components and create colour‐coded images from these parameters, see Section 2.6.

Natural fluorescence lifetimes of organic dyes and fluorescence proteins are in the range of a few nanoseconds. It is therefore often believed that a time resolution just below 1 ns is sufficient to resolve the fluorescence decay functions of biological material. This is not generally correct. The interacting‐donor component of FRET can be below 400 ps, the fast components of NAD(P)H and FAD below 300 ps, the lifetimes of carotenoids and other natural fluorophores on the order of 20 ps and less [12, 13], and the lifetime of melanin can be shorter than 10 ps [14, 15].

## 2 Lifetime Imaging Techniques

Lifetime Imaging (FLIM) techniques can be classified into wide‐field and scanning techniques, time‐domain and frequency‐domain techniques, and analogue and digital (photon counting) techniques [16, 17, 18, 19]. All possible combinations are in use and violently defended by their originators. We will therefore restrict the discussion here to the most important features.

### 2.1 Wide‐Field Techniques

Wide‐field techniques illuminate the entire sample by the excitation light and project an image of the emission light on a camera‐like detector. This can be a gated or modulated image intensifier or an array of single‐photon avalanche photodiodes.

An advantage of wide‐field FLIM is that the data in the pixels are acquired simultaneously. Compared to scanning techniques, wide‐field techniques therefore can, in principle, achieve shorter acquisition time. Moreover, the optical setup is relatively simple, which is an advantage when FLIM is used in combination with TIRF [20], Nipkow–Disk scanning [21], light‐sheet microscopy [22] or endoscopes [23], see Section 4.12.

However, there are also disadvantages: The penetration depth into biological tissue is small, and the image contrast is impaired by scattering (Section 2.2; Figure 8). The imaging process itself does not provide any depth resolution or optical sectioning capability and does not suppress light scattered in the sample. Another problem can be glass fluorescence. In contrast to scanning, where the pinhole rejects fluorescence in the optics, in a wide‐field setup glass fluorescence directly reaches the detector. This is a problem especially when FLIM is recorded at UV excitation wavelength, and when FLIM is recorded through an endoscope. A solution is to excite through a separate optical pathway [23] (Section 4.12).

In principle, rejection of out‐of‐focus signals can be obtained by structured illumination techniques [24, 25]. However, these techniques are based on calculating differences between images recorded for different illumination patterns. The process adds up the photon noise in the images of the different excitation patters. The photon efficiency (see Section 1) is therefore low. The problem of missing depth resolution can be overcome by a light sheet excitation geometry [26], TIRF [20, 27] or Nipkow–Disk scanning [21]. These techniques work without much loss in photon efficiency but need relatively complex optical systems.

Another possibility is to use a combination of scanning and multifocus multiphoton excitation [28]. By doing so, the technique loses part of the advantages of wide‐filed imaging, namely the simplicity of the optical setup and the recoding speed. True wide‐field two‐photon excitation with depth resolution has been achieved by ‘temporal focusing’ [29]. The technique uses the fact that the spectral width of a femtosecond beam correlates inversely with the pulse width. By sending sub‐beams of different wavelengths through different parts of the microscope lens short pulse width—and thus efficient two‐photon excitation—is obtained only in the depth of the sample where the sub‐beams combine. Temporal focusing requires extremely high laser power which precludes the use on most biological samples.

#### 2.1.1 Modulated and Gated Image Intensifiers

Image intensifiers are vacuum devices containing a photocathode, an electron multiplication system, and a spatially resolved readout element. The image is projected on the photocathode, the photoelectrons are multiplied in a multichannel plate (or two multichannel plates in series), and the multiplied electrons are recorded either directly in a semiconductor camera chip inside the tube or converted into light by a luminescent screen and the detected by a CMOS or CCD camera outside the tube. Time resolution is achieved by gating or modulating the electron multiplication in the tube.

**FIGURE 4:**
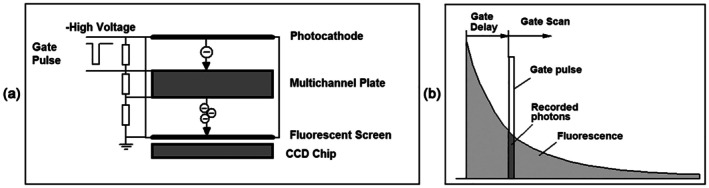
Gated image intensifier. (a) Electronic principle. (b) Shift of the gate pulse over the fluorescence waveform.

The principle of the gated intensifier is shown in Figure 4a. The sample is excited by a pulsed light source. Normally this is a high‐repetition‐rate laser but image intensifiers can work also with low‐repetition‐rate lasers, such as Nitrogen lasers or Q‐switched Nd‐YAG lasers [23, 30]. A fluorescence image of the sample is projected onto the photocathode of a gated image intensifier. Photoelectrons emitted by the photocathode are accelerated towards a multichannel plate (MCP). In the MCP they are multiplied by secondary‐electron emission. The multiplied electrons leaving the MCP are accelerated towards the anode of the intensifier. In the original design, the anode was a fluorescent screen on which the electrons created an intensified image resembling the image at the photocathode. This image is detected by a CCD camera outside the tube. In the modern implementation, a CCD chip is placed inside the tube, and the image is created directly by the electrons. Gating is performed by pulsing the voltage between the photocathode and the MCP or an additional grid in between. To record the waveform of the input signal the image intensifier is gated with a fast electrical pulse synchronously with the pulsing of the light source. The gate pulse is shifted over the time interval of the fluorescence‐decay function (Figure 4b). This way, images are obtained for different time intervals within the fluorescence decay [30, 31]. It is also possible to project several images of the sample on the image intensifier with different optical delays and record them with one gate pulse [32]. Depending on the number of gate intervals the fluorescence lifetime is calculated by ratio calculation [33], first‐moment calculation [34] or iterative convolution and fitting the data with a suitable decay model (Section 2.6). For early implementations and applications, please see [8, 30, 35, 36, 37], for signal‐to‐noise considerations [38, 39, 40].

**FIGURE 5:**
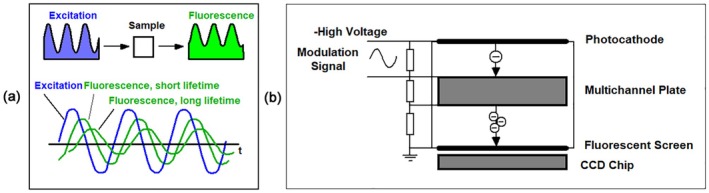
FLIM by a modulated image intensifier. (a) Principle of modulated fluorescence excitation and emission. (b) Principle of modulated image intensifier.

The modulation technique is based on modulated excitation of the sample and measuring the amplitude and the phase of the fluorescence light. When a sample is excited with modulated light amplitude and phase of the fluorescence signal changes in a characteristic way: Short lifetime delivers a small phase and large amplitude, long lifetime large phase and small amplitude, see Figure 5, left. To record the data, a fluorescence image of the sample is projected on the photocathode of the image intensifier. The principle of the intensifier is shown in Figure 5, right. By applying a modulation voltage between the photocathode and the MCP the electron transfer efficiency is modulated with the same frequency as the light source. The phase of the modulation voltage compared to the excitation is changed, and several images are recorded for different phase shifts. From the images, the amplitude and the phase of the emission light compared to the excitation are calculated [41]. The amplitudes and phases are used for frequency‐domain (phasor) analysis [42], Section 1. For signal‐to‐noise considerations, please see [38, 40].

#### 2.1.2 Externally Gated CMOS Cameras

In the past 10 years, conventional imaging has been almost exclusively taken over by semiconductor imaging chips. Pixel numbers are in the Megapixel range. Time‐gating or shutter functions of these chips, if implemented at all, are far from the resolution required for FLIM [43]. It has therefore been attempted to gate the detection signal externally of the detector. The optical signal in front of the detector is gated by an electro‐optical modulator (EOM) and the gate signal is shifted in small increments with reference to the excitation pulses. This way, a series of images for different times in the excitation pulse period is obtained. The problem is that it is not possible to obtain sub‐ns gate duration with an EOM. According to [44] the effective IRF width is about 6 ns (full width at half maximum). This may be enough to detect the lifetime of a single‐exponential approximation of the fluorescence decay (the ‘apparent’ lifetime) but it is not enough to split a multi‐exponential decay into its components. Lifetime sensitivity was reported to be 2.53 ps with 10^7^ photons per frame. With an IRF width of a few ns, it must be supposed that this is the accuracy of the fluorescence lifetime averaged over the entire image.

#### 2.1.3 SPAD Arrays

A new level of fast wide‐field imaging has been reached by image sensors based on single‐photon avalanche diode (SPAD) arrays. The SPADs detect optical signals at the single‐photon level and at a time resolution in the ps range. The timing information provided by the SPADs is made available either by gating [45, 46, 47, 48] the entire array or by processing the photon times by time‐to‐digital converters (TDCs) connected to the individual SPADs [26, 49, 50].

**FIGURE 6:**
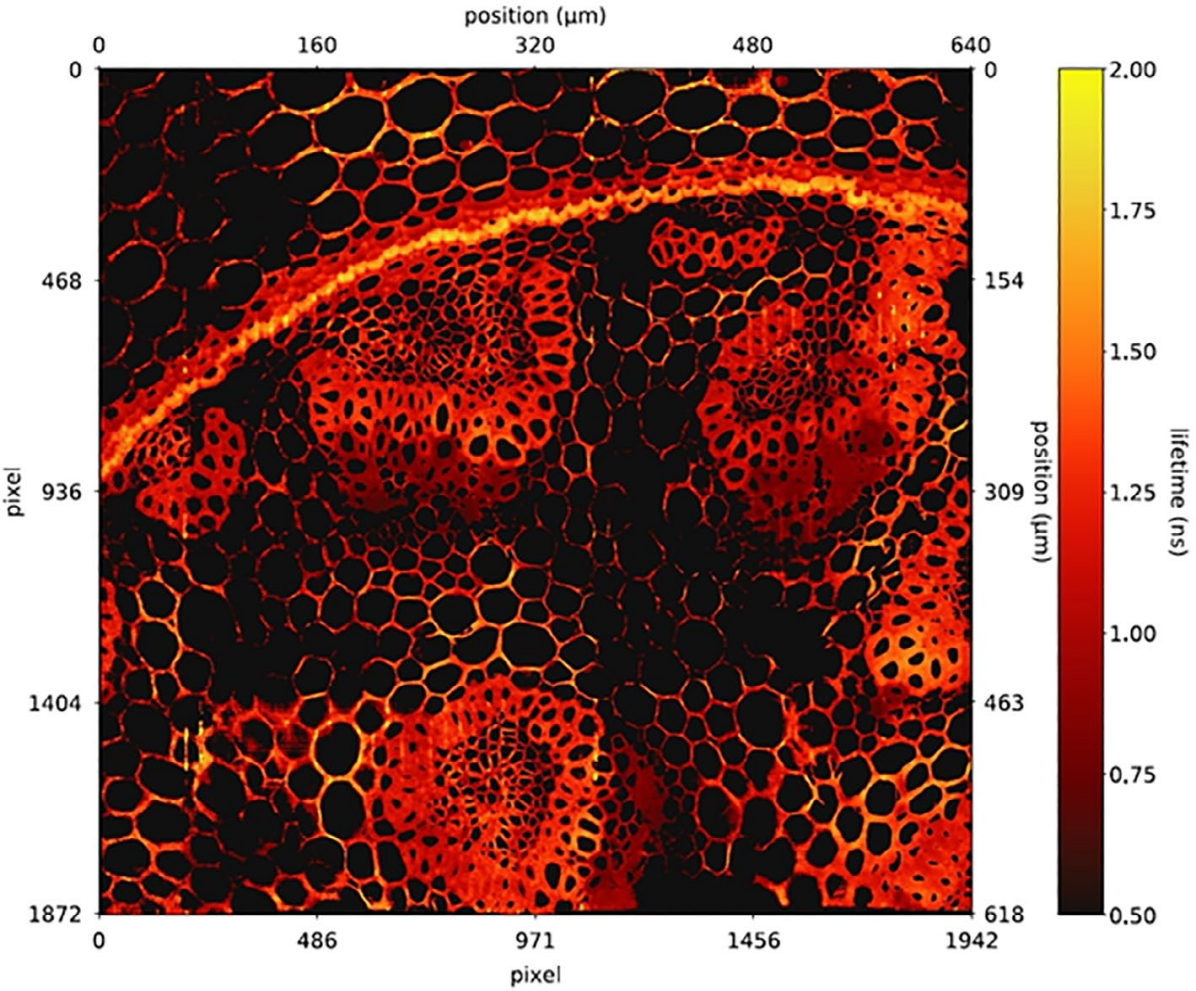
FLIM image recorded by gated SPAD array. Gate width 3.8 ns, 8 × 4 images stitched together. Image size 618 × 650 μm. From [51].

When gated detection is used, photon numbers in different time windows of the pixels are recorded, and lifetimes are determined by ratio calculation, first‐moment calculation or fit procedures. The problem with the gated SPAD detector is time resolution. The resolution is limited by the shortest gate duration the detector can achieve, and by the loss in photon efficiency with decreasing gate time. High resolution can be obtained only by narrow gate pulses. Resolution then comes at the expense of efficiency because most of the photons are gated off. It has been attempted to use wide gate pulses which are shifted in increments smaller than the gate width. The decay curve then has to be reconstructed by calculating differences between the corresponding intensities. However, this results in an increase in noise and, again, in a decrease in photon efficiency. An example of a Gated‐SPAD image is shown in Figure 6. The image is a composite of 8 × 4 images from a 500 × 1024 pixel array [51]. It was acquired with an effective gate width of 3.5 ns. This is enough to determine a single‐exponential approximation of the pixel lifetimes but not enough for multi‐exponential decay analysis.

Higher time resolution is obtained by TDC‐based SPAD arrays. Each pixel has an on‐chip TDC connected to it. For each detected photon, the TDC delivers the time in the excitation pulse period. TDC‐based sensors achieve high time resolution in combination with reasonable photon efficiency [26, 49, 50]. However, TDC‐based SPAD sensors are difficult to build with high pixel numbers. Currently, TDC‐based SPAD arrays are mainly used as confocal detectors in combination with scanning. Parallel detection of the spatial intensity distribution in several sub‐pixel elements within the excited spot helps reduce pile‐up and dead‐time effects at high count rate [52], and allows associated software to calculate images of diffraction‐limited resolution without the need for extremely small pinholes [53, 54].

A problem is that the TDCs or the signal lines to the TDCs occupy a large fraction of the active area. For pixel numbers larger than 256 × 256 as they are typically used in microscopy, this leads to a low ‘fill factor’, and, consequently, low detection efficiency. The effective fill factor can be increased by an array of micro lenses in front of the detector chip [49]. However, the lens array can only be configured for a given (normally low) NA of the optics which projects the image on the chip. If the projection optics have to de‐magnify the image, as is the case in a wide‐field microscope, the NA is high, resulting in sub‐optimal efficiency of the array.

#### 2.1.4 Wide‐Field TCSPC

Except for SPAD arrays with internal TDCs, wide‐field FLIM does not reach the time resolution of TCSPC FLIM (Section 2.5.1). Moreover, compared to SPAD arrays—both gated and TDC‐based—the background count rate per pixel is several orders of magnitude smaller. It has therefore been attempted to combine wide‐field imaging with multi‐dimensional TCSPC.

**FIGURE 7:**
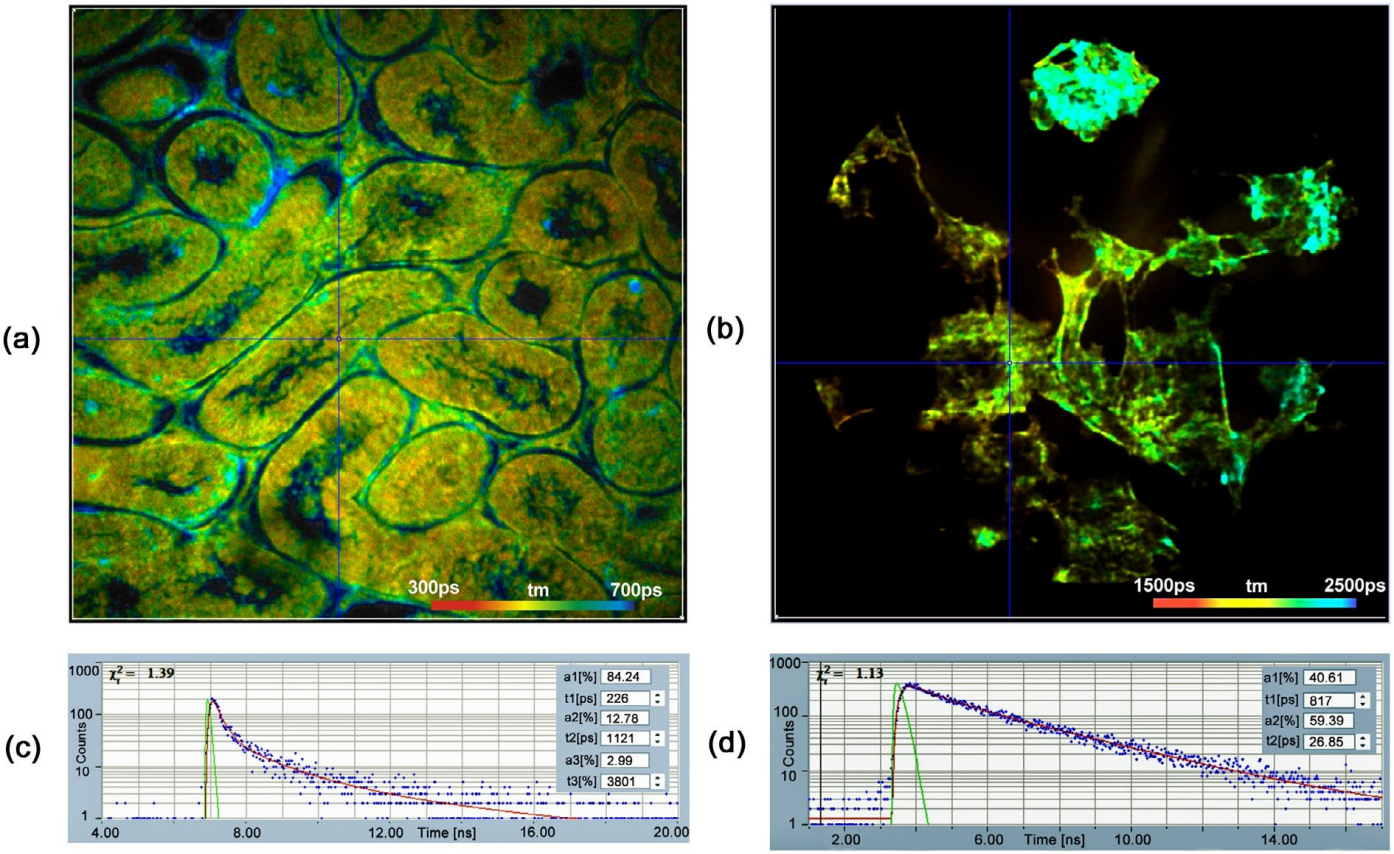
Wide‐field FLIM with position‐sensitive TCSPC detectors. (a) Mouse tissue slice stained with acridine orange, recorded with resistive‐anode MCP. (b) BPAE (bovine pulmonary artery) cells, stained with Alexa 488, recorded with delay‐line anode MCP. (c, d) Decay curves in a selected spot in (a, b) with the IRF in green, data points in blue and best‐fit line in red. IRF widths are different due to different systems. Image sizes are approximately 100 × 100 μm.

An image is projected on the photocathode of an image intensifier tube and the photoelectrons are multiplied by several multichannel plates (MCP). The anode of the tube is a resistive layer [55] or a system of crossed delay‐lines [56]. For every photon, the electronics measure the electrical charge at the four sides of the resistive anode or the delay of the pulses between the outputs of the delay lines. Together with the measurement of the arrival time relative to the excitation pulse a photon distribution over the image coordinates and the time in the decay curve is built up [4, 20, 56, 57]. Images and decay curves recorded with the two sensor types are shown in Figure 7. The FLIM data have TCSPC‐typical time resolution and very good spatial resolution. A light sheet FLIM microscope based on the delay‐line detector has been described by Hirvonen et al. [22].

### 2.2 FLIM Techniques Based on Scanning

#### 2.2.1 Advantages of Scanning

Confocal and multiphoton laser scanning delivers images from a defined focal plane in the sample. The sample is scanned by a focused laser beam. Excitation can be performed by a one‐photon or by a multiphoton process, please see Figure 1. In the first case, the fluorescence is fed back through the scanner, sent through a pinhole and projected on the detectors(s). In the second case, the fluorescence is separated from the excitation by a dichroic mirror already behind the microscope lens and sent to the detectors directly. Fluorescence from unwanted sample planes is suppressed by the pinhole or, in the case of multiphoton imaging, not excited at all. This ‘optical sectioning’ capability is extraordinarily important in FLIM applications: Fluorescence decay functions recorded from biological objects are usually multi‐exponential. The decay components have to be separated to obtain quantitative FRET results, distinguish different proteins, different metabolic states or to derive biochemical information from autofluorescence. Separating several decay components is generally difficult and requires a large number of photons in the pixels of the image [5, 58]. The situation becomes virtually hopeless if the pixels not only contain fluorescence of several fluorophore populations but also fluorescence from different sample layers. Suppression of out‐of‐focus signals is therefore a precondition for obtaining meaningful lifetime images.

However, optical sectioning capability is not the only advantage of scanning. Scanning also avoids crosstalk between the pixels by scattering. Scattering is not important for imaging thin and transparent samples, as they are usually considered in conventional microscopy. It is, however, the dominating process of image deterioration in thick samples, such as mammalian skin, bone, or other tissues.

**FIGURE 8:**
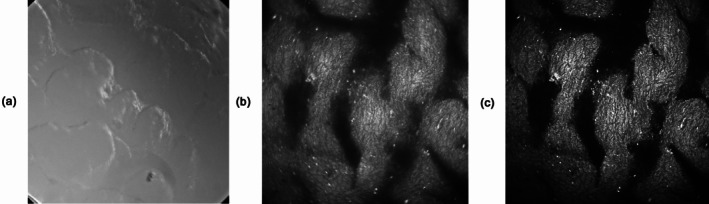
(a) Wide‐field image; (b) non‐confocal scan image; (c) confocal image. Image size: 220 × 220 μm.

An example is shown in Figure 8. The left image (a) shows a conventional image of pig skin taken through a microscope. The image shows some surface details of the sample but the internal structure is entirely hidden. Scanning with a focused laser beam yields the image in Figure 8b. It clearly shows the internal tissue structure. Please note that the image was *not* taken through a confocal pinhole. The contrast is still impaired by out‐of‐focus haze, which clearly can be seen in the image. The image in Figure 8c was taken through a confocal pinhole. The pinhole also removes the out‐of‐focus haze, resulting in a clean image of the focal plane scanned.

Why is the image quality so much better for scanning? The reason is scattering. A scanning device excites only one pixel at a time. Consequently, it records scattered light *only from the scanned pixel* but not from the other pixels of the image. A wide‐field imaging system excites all pixels simultaneously. In every pixel, it detects scattered light not only from *this* pixel, but also from *all other pixels* of the illuminated area. Considering the fact that a good microscopy image has at least 500 × 500 = 250,000 pixels it becomes clear why the wide field image does not show much detail from inside the tissue.

#### 2.2.2 Frequency‐Domain FLIM

Frequency‐domain FLIM records the amplitude and the phase of the detected signal at the fundamental frequency (the first Fourier component) of the laser pulse sequence. Early implementations used gain‐modulated photomultipliers in a heterodyne setup to determine phase and amplitude [18, 59]. The modern implementation uses the timing of single photons and derives the phasors from calculating sine‐ and cosine‐weighted moments of the photon density over the time in the laser pulse period.

**FIGURE 9:**
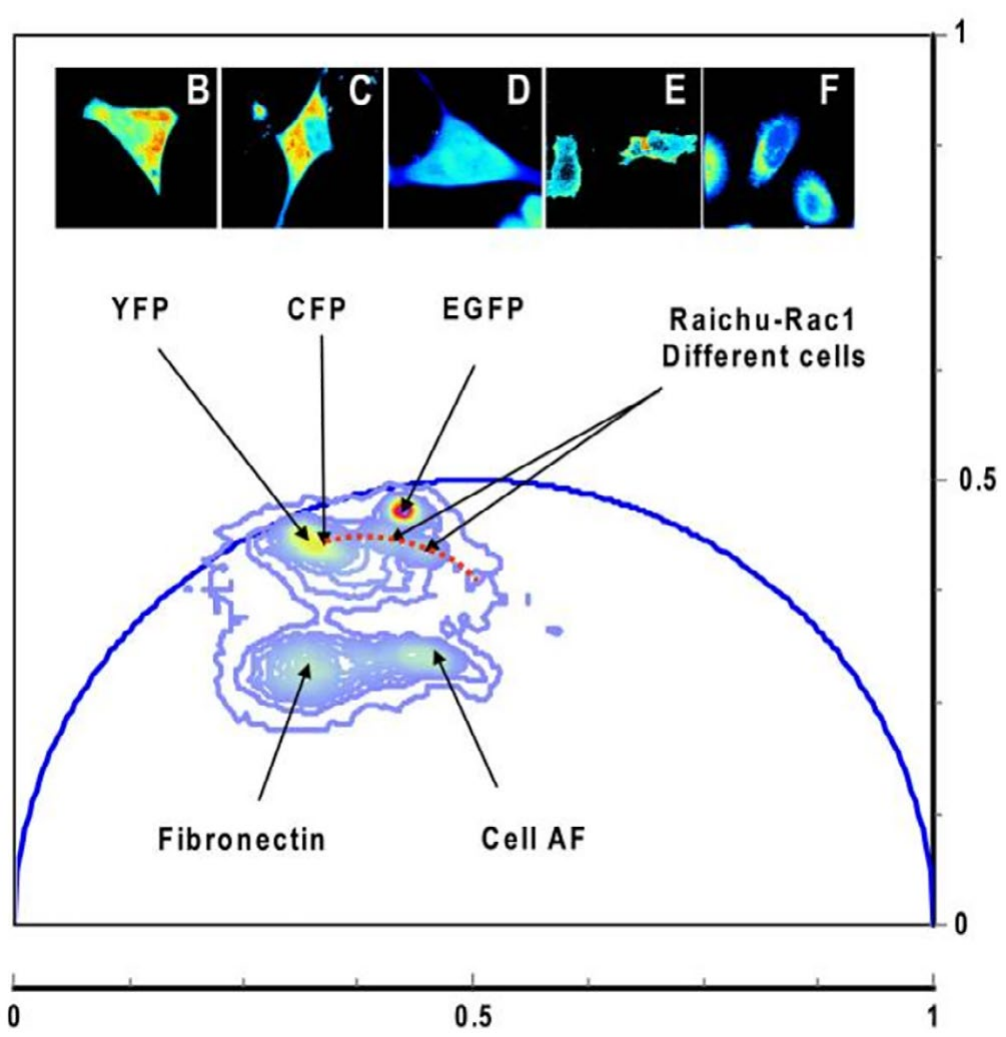
FLIM data obtained by frequency‐domain FLIM where YFP, CFP and EGFP are yellow, cyan and enhanced green fluorescent protein, respectively and AF is autofluorescence (from [60]).

With increasing lifetime the amplitude decreases and the phase increases. Amplitudes and phases of the individual pixels are considered pointers (or ‘Phasors’) in a plane of complex numbers. The phasors can, in principle, be transformed back into fluorescence decay times. However, normally they are used directly in a ‘Phasor Plot’, see Figure 9. Pixels of different fluorescence lifetimes form clusters in the phasor plot. These can be back‐annotated in the image as false colour, see upper row of see Figure 9. Please see Section 2.6 for details.

#### 2.2.3 Gated Photon Counting

Gated photon counting records photon numbers in a few (usually no more than four) time intervals distributed over the decay functions [61]. Scanning is made slow enough so that the data from the counters can be read at the end of each pixel. Fluorescence decay times are calculated from the photon numbers in the gates. Because the data are recorded in all time gates in parallel the technique reaches a good photon efficiency [39]. The maximum count rate is very high, and only limited by the counting capability of the detector. The time resolution is limited by the minimum gate width, which usually is 0.5–1 ns. Faster gates and more gate time intervals could possibly have been achieved by modern FPGA techniques, if the technique had not been replaced with advanced TCSPC techniques.

#### 2.2.4 Analogue‐Waveform Detection

Scanning procedures have been combined with the recording of analogue signals delivered by linear detectors. The techniques are mostly driven by an over‐estimation of the available photon rates and the size of pile‐up in TCSPC FLIM systems. Analogue detection may also be dictated by low‐repetition rate excitation sources which make photon counting impossible [62, 63, 64, 65]. The sample is excited by a pulsed laser, the emission from the sample is detected by a PMT or MCP PMT, and the output signal is fed into a fast digitizer. The waveform recorded by the digitizer is then stored in a pixel‐related data array.

In contrast to TCSPC, where the IRF is the transit‐time‐spread of the detector, the IRF of analog detection is the single‐photon response. The single‐photon response is 10–20 times broader than the transit‐time spread. Consequently, the technique suffers from low time resolution and the inability to resolve fast components of multi‐exponential decay functions.

#### 2.2.5 TCSPC FLIM

Classic TCSPC is based on the excitation of a sample by a high‐repetition‐rate light source, the detection of single photons of the fluorescence signals, the measurement of the photon arrival times in the excitation pulse period, and the build‐up of a photon distribution over these times [4, 16, 66]. TCSPC delivers extremely high time resolution. In contrast to analogue recording techniques, the time resolution is not the width of the temporal single‐photon response of the detector but the average time jitter of the pulses [4, 16]. The jitter is at least an order of magnitude smaller than the width of the pulses. By recording all photons which are seen by the detector TCSPC achieves near‐ideal sensitivity. The photon efficiency, that is, the accuracy for a given number of recorded photons, is the highest of all time‐resolved optical recording techniques [38, 39, 40]. However, the classic TCSPC process is one‐dimensional: it only delivers a single fluorescence decay curve at a single spatial position of the sample. Nevertheless, classic TCSPC has been used for FLIM in combination with laser scanning. A decay curve was recorded at one spatial position, the data saved, and then the scanner moved to the next position. The process was repeated until decay day data for all pixels of the image had been recorded [67]. Naturally, the imaging process was very slow, and not compatible with the fast scan of modern laser scanning microscopes.

**FIGURE 10:**
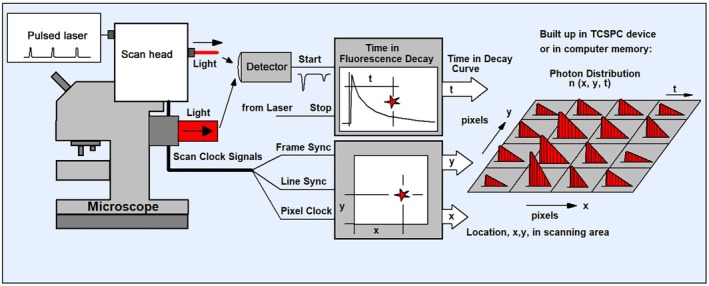
FLIM by laser scanning and multi‐dimensional TCSPC.

**FIGURE 11:**
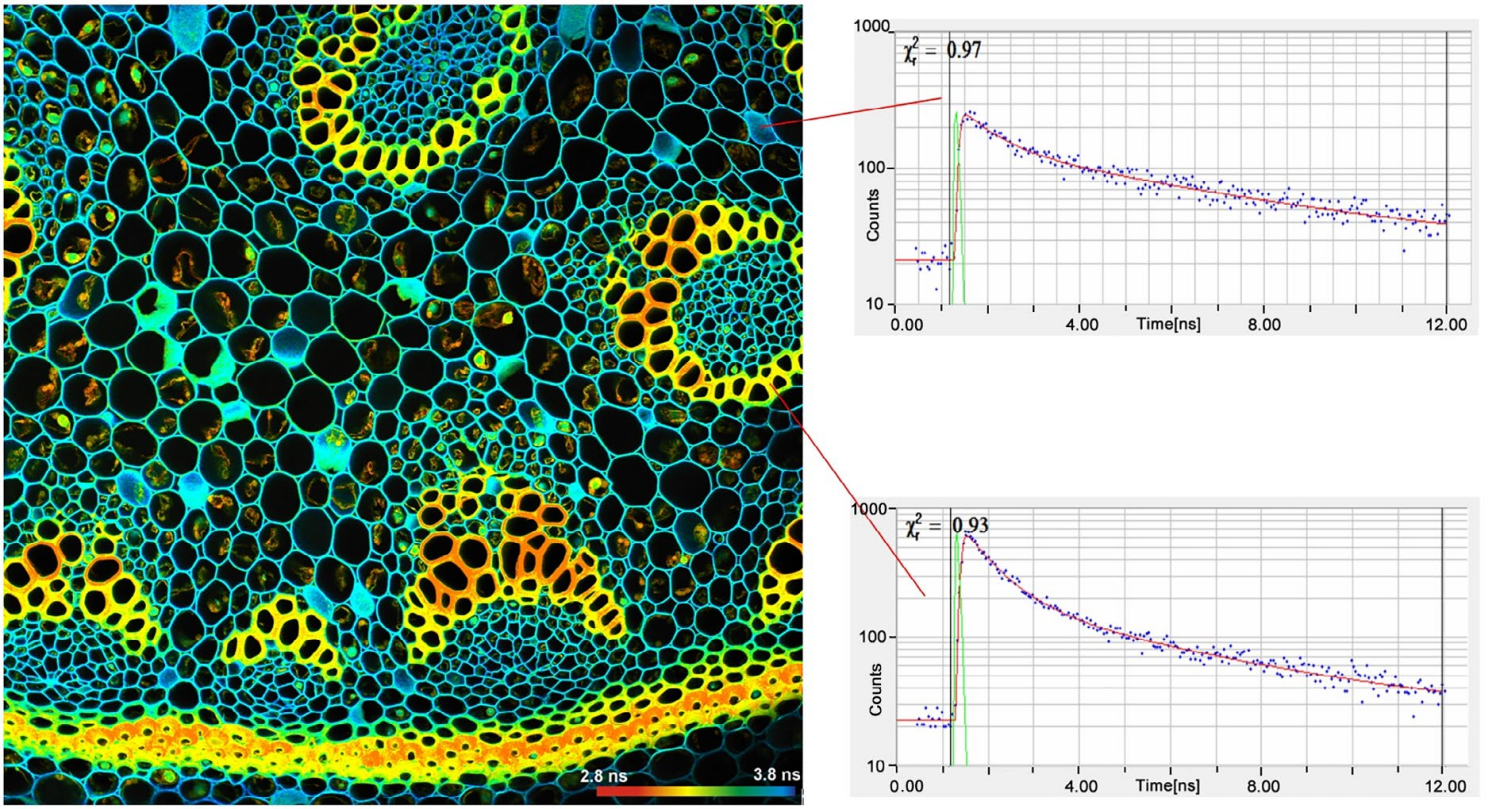
TCSPC FLIM image. Mean (amplitude weighted) lifetime of double‐exponential decay. Image size: 715 × 715 μm (1024 × 1024 pixels). Right: decay curves in selected spots of the image. Blue dots are photon numbers in the TCSPC time channels, red curves are the model functions fit to the TCSPC data and green curves are the IRF. IRF width is 97 ps.

The problem of fast scanning was solved by making the recording process of TCSPC multi‐dimensional [4, 16, 68]. Each photon is characterised not only by its time in the laser pulse period but also by the position, *x*, *y* of the laser spot in the moment of the photon detection. The recording process builds up a photon distribution over these parameters. The result can be interpreted as an array of pixels, each containing a full fluorescence decay curve. Importantly, the recording process is independent of the scan rate: When a photon is detected the electronics determines the time and the position and puts the photon in the corresponding memory location of the photon distribution. The principle is illustrated in Figure 10, a typical result is shown in Figure 11.

Even in its basic configuration, TCSPC FLIM is characterised by near‐ideal sensitivity, near‐ideal photon efficiency, fast recording, extremely high time resolution, capability to resolve multi‐exponential decay profiles and optical sectioning capability when combined with confocal or multiphoton laser scanning microscopes. Moreover, due to its digital nature, the results are exactly described by Poisson statistics. Well‐defined statistics not only make the signal‐to‐noise ratio exactly predictable, but it also allow analysis software to derive unbiased decay parameters at an accuracy limited only by the number of photons in the current pixel, please see Section 1.

#### 2.2.6 Multi‐Dimensional TCSPC FLIM

TCSPC FLIM can be extended by measuring additional parameters of the photons and adding them as additional dimensions to the photon distribution. The parameters can be wavelength, polarisation, time from the start of an experiment, time from a stimulation of the sample or the time within the period of a modulation of the laser. Depending on which additional parameters are recorded, the result is multi‐wavelength FLIM, wavelength multiplexed FLIM, recording of fast dynamic lifetime effects and simultaneous FLIM/PLIM.

##### 2.2.6.1 Multi‐Wavelength FLIM

**FIGURE 12:**
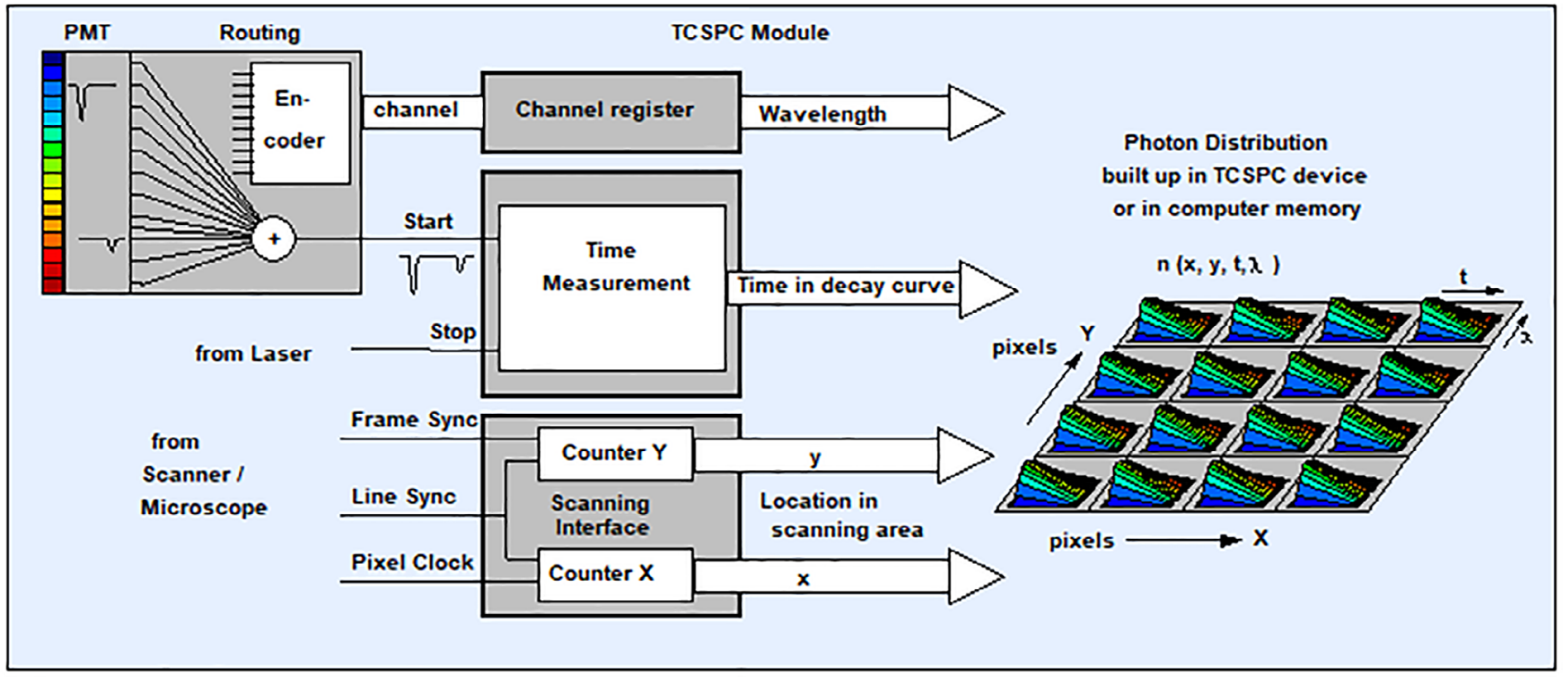
Principle of multi‐wavelength FLIM.

**FIGURE 13:**
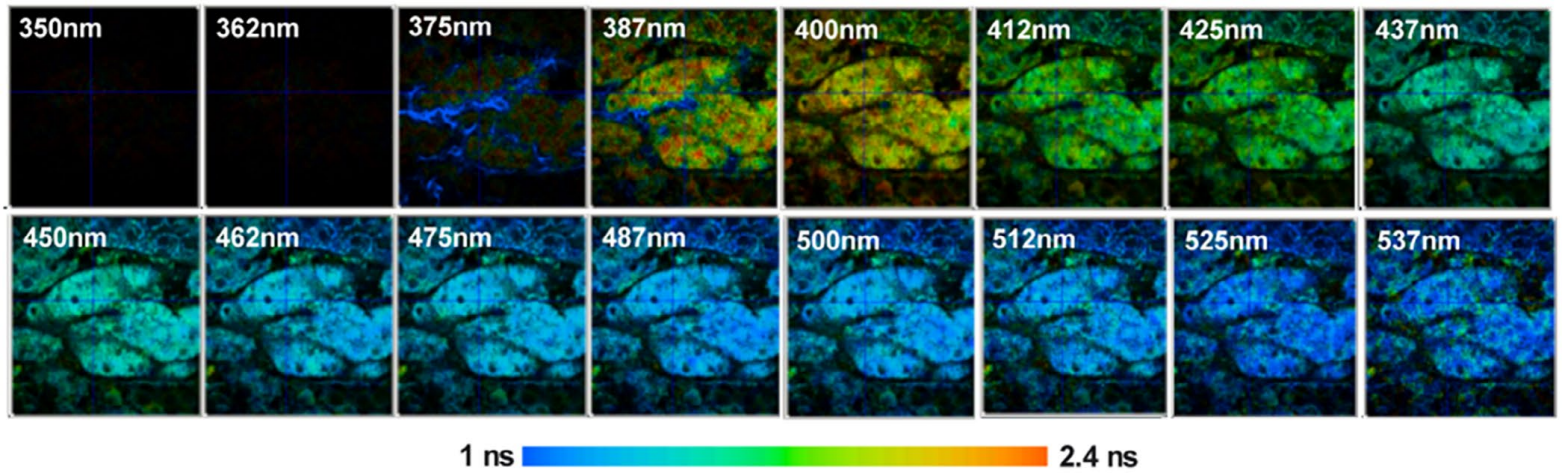
Multi‐wavelength fluorescence lifetime images of a mouse‐kidney section. Autofluorescence, two‐photon excitation at 750 nm. Images in subsequent wavelength intervals from 350 nm to 537 nm. Image sizes are 200 × 200 μm, 256 × 256 pixels (from [4]).

Multi‐wavelength FLIM (also called multi‐spectral FLIM) uses an extension of the principle shown in Figure 10. A spectrum of the fluorescence light is spread over an array of detector channels. Usually, these are the channels of a multi‐anode PMT, or an array of single‐photon avalanche diodes. In addition to the times of the photons and the positions, *x*, and *y*, of the scanner, the TCSPC module determines the detector channel that detected the photon. These pieces of information are used to build up a photon distribution over the time of the photons in the fluorescence decay, the wavelength and the coordinates of the image [4, 16, 69]. The result is an image that contains several decay curves for different wavelengths in each pixel [69, 70, 71, 72, 73, 74, 75, 76, 77, 78] (Figure 12). With a fibre–bundle interface [4] the technique works efficiently not only at confocal ports of one‐photon microscopes but also at non‐descanned ports of multiphoton microscopes. A practical example is shown in Figure 13. It shows the intensity‐weighted lifetime of a mouse kidney sample. The data quality of multi‐wavelength FLIM is sufficient for double exponential decay analysis [4, 16].

Multi‐wavelength FLIM with a 256 × 2 pixel SPAD/TDC array has been described in [79]. Different from the technique described above, it uses fully parallel detection in the 256 channels of the SPAD array. This gives the system high spectral resolution and high throughput. Due to the small size of the sensor elements, the sensor works with one‐photon confocal scanning but not with multiphoton excitation and NDD (non‐descanned detection) optics.

##### 2.2.6.2 Excitation–Wavelength Multiplexing

TCSPC FLIM can be used with excitation‐wavelength multiplexing. Several lasers are multiplexed synchronously with the frames, lines, or pixels of the scan. Each photon is marked with an identifier of the laser that was active when it was recorded. The photons are then ‘routed’ into different memory blocks so that separate images for the different lasers are built up [4, 16]. The technique is free of wavelength crosstalk. A typical application is metabolic FLIM of NADH and FAD: Two ps diode lasers, with wavelengths of 375 and 440 nm are multiplexed, and the data are recorded in two parallel TCSPC FLIM channels at different detection wavelengths [4, 80, 81, 82, 83]. The principle works also with two‐photon excitation by two femtosecond fibre lasers [4]. It can also be used to multiplex fluorescence excitation with illumination of an operation area [82, 84, 85].

#### 2.2.7 Recording of Dynamic Lifetime Effects

##### 2.2.7.1 Record‐and‐Save Procedure

Recording dynamic changes in the fluorescence lifetime of a sample requires some kind of time‐series recording. In the simplest case, a FLIM image is recorded, the data are saved on a mass storage device, then the next image is recorded, and so on. This ‘record‐and‐save’ procedure is applicable to all lifetime imaging techniques. The speed at which the series can be recorded is determined by the time it takes to acquire a sufficient number of photons from the sample and by the time necessary to save the data [4].

##### 2.2.7.2 Temporal Mosaic FLIM

**FIGURE 14:**
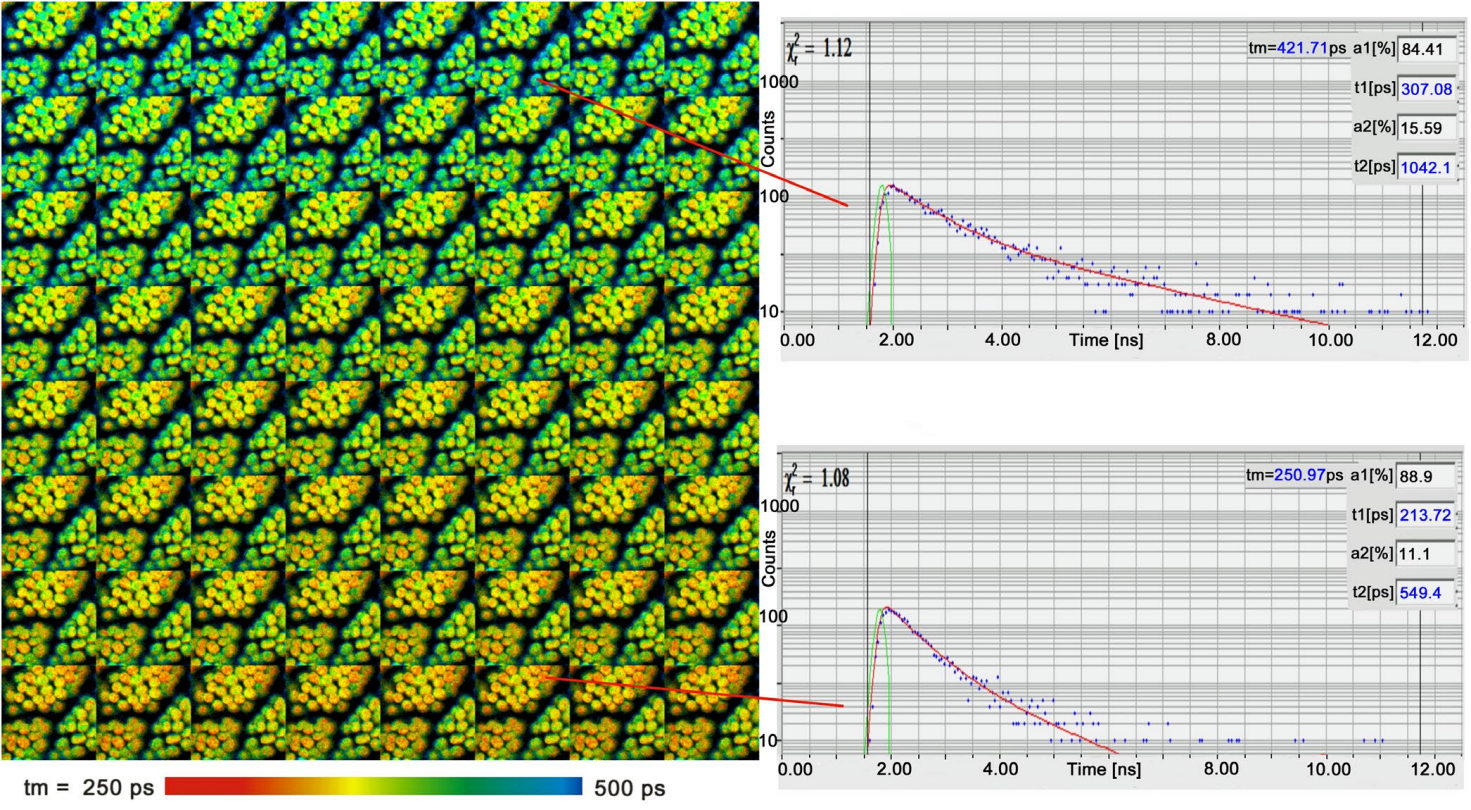
Time series acquired by mosaic FLIM. Recorded at a speed of 1 mosaic element per second. 64 elements, each element 128 × 128 pixels, 256‐time channels, double‐exponential fit of decay data. Size of each element is 40 × 40 μm. Sequence starts at the upper left. Moss leaf, lifetime changes by non‐photochemical chlorophyll transient.

Mosaic FLIM was originally intended to record spatial mosaics with mechanically driven sample stages [4, 86]. However, mosaic FLIM can also be used for time series recording [4], such as recording of chlorophyll transients and fluorescence recovery аfter photobleaching (FRAP) trajectories. After the start of the experiment, the FLIM system takes consecutive scans of the same sample area and stores the data in consecutive elements of the mosaic (Figure 14). The advantage is that no time has to be reserved for saving the data of the individual scans. Moreover, as the result is one large photon distribution, data analysis of the entire mosaic can be performed in a single data analysis run. That means all mosaic elements are analysed with the same fit parameters, and image segmentation or global analysis procedures can be applied to the whole mosaic.

##### 2.2.7.3 Triggered Accumulation of Time Series

**FIGURE 15:**
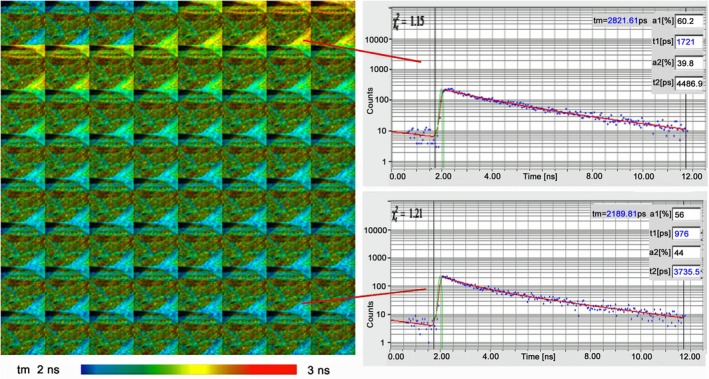
Ca^2+^ transient in cultured neurons, incubated with Oregon Green Bapta. Electrical stimulation, stimulation period 3 s, data accumulated over 100 stimulation periods. Image size per element: 50 μm. Time per mosaic element is 38 ms.

The biggest advantage of temporal mosaic FLIM is that the data can be accumulated. The recording procedure would be triggered with a (periodic) stimulation of the sample. Starting with the trigger, the recording runs through all elements of the mosaic and then stops. When the sample has recovered from the previous stimulation the procedure is repeated. The data from a large number of stimulation periods are accumulated in the same data mosaic. The result is that the number of photons in the pixels, that is, the signal‐to‐noise ratio, no longer depends on the speed of the sequence. Instead, it depends on the total acquisition time (and, of course, on the photon rate and the number of pixels per mosaic element). Extremely fast time series can be recorded without the need for extreme excitation power. The procedure is therefore friendly to live cells. The speed of the sequence is limited only by the scan rate. For fast galvanometer scanners operating at medium numbers of pixels, the frame time can be faster than 50 ms [87]. A triggered‐accumulation series of a Ca^2+^ transient in cultured neurons is shown in Figure 15. Time per image is 38 ms, which is considerably faster than traditional FLIM implementations. Even faster recordings are possible by line scanning. By building up a photon distribution along a line scan, fluorescence‐lifetime transient scanning (FLITS) reaches a transient‐time resolution of about 1 ms [87, 88, 89].

##### 2.2.7.4 Simultaneous FLIM/PLIM

Phosphorescence is usually not perceptible in organic dyes, but it can be the dominating decay process for specially designed phosphorescence markers. Phosphorescence decay times range from microseconds to milliseconds. Notably for applications in biology, phosphorescence lifetimes depend on the oxygen partial pressure. Phosphorescence dyes therefore make excellent oxygen sensors. Early applications are described in [90, 91, 92, 93, 94, 95]. Please see [4] for more references.

The problem with phosphorescence‐lifetime imaging (PLIM) is that the decay times are longer (usually much longer) than the excitation pulse periods of the excitation sources used for FLIM. Early PLIM systems therefore used low‐repetition rate light sources and gated image intensifiers, gated photon counting, or simply fast cameras to detect phosphorescence lifetimes. A recent PLIM system using a photon‐counting camera of nanosecond time resolution has been described in [96]. The reduction of the excitation pulse rate was a problem also on the laser side, especially when two‐photon excitation had to be used. Moreover, a direct combination with FLIM recording was not possible.

**FIGURE 16:**
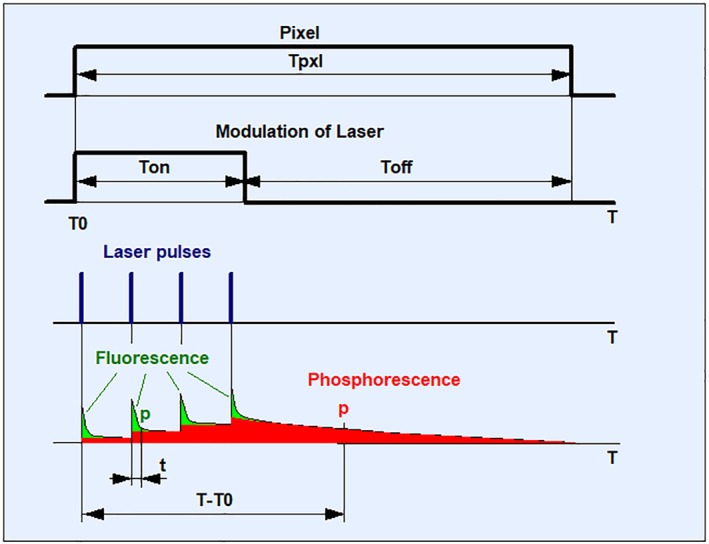
Principle of simultaneous FLIM. *T*_0_: time, when the laser is switched on; *T*_on_: time interval when the laser is switched on. *T*_off_: time interval when the laser is switched off. *T*_pxl_: time duration of a pixel. *t*: Photon time in laser period. *T*–*T*_0_: Photon time in modulation period.

A TCSPC process that delivers both FLIM and PLIM was introduced in 2010 [4, 97, 98, 99, 100, 101]. The principle is shown in Figure 16. The technique is based on modulating a high‐frequency pulsed laser synchronously with the pixel clock of the scanner. Photon times are determined both with reference to the laser pulses and the laser modulation period. Fluorescence is recorded during the ‘on’ time, and phosphorescence during the ‘off’ time of the laser. The technique does not require a reduction of the laser repetition rate, works with two‐photon excitation and non‐descanned detection, and delivers an extremely high PLIM sensitivity. Unlike other scanning PLIM techniques, it does not cause moiré patterns in the images. A typical result is shown in Section 3.6.6 (Figure 28). For detailed description and application references, please see [4, 101].

### 2.3 Fast FLIM

**FIGURE 17:**
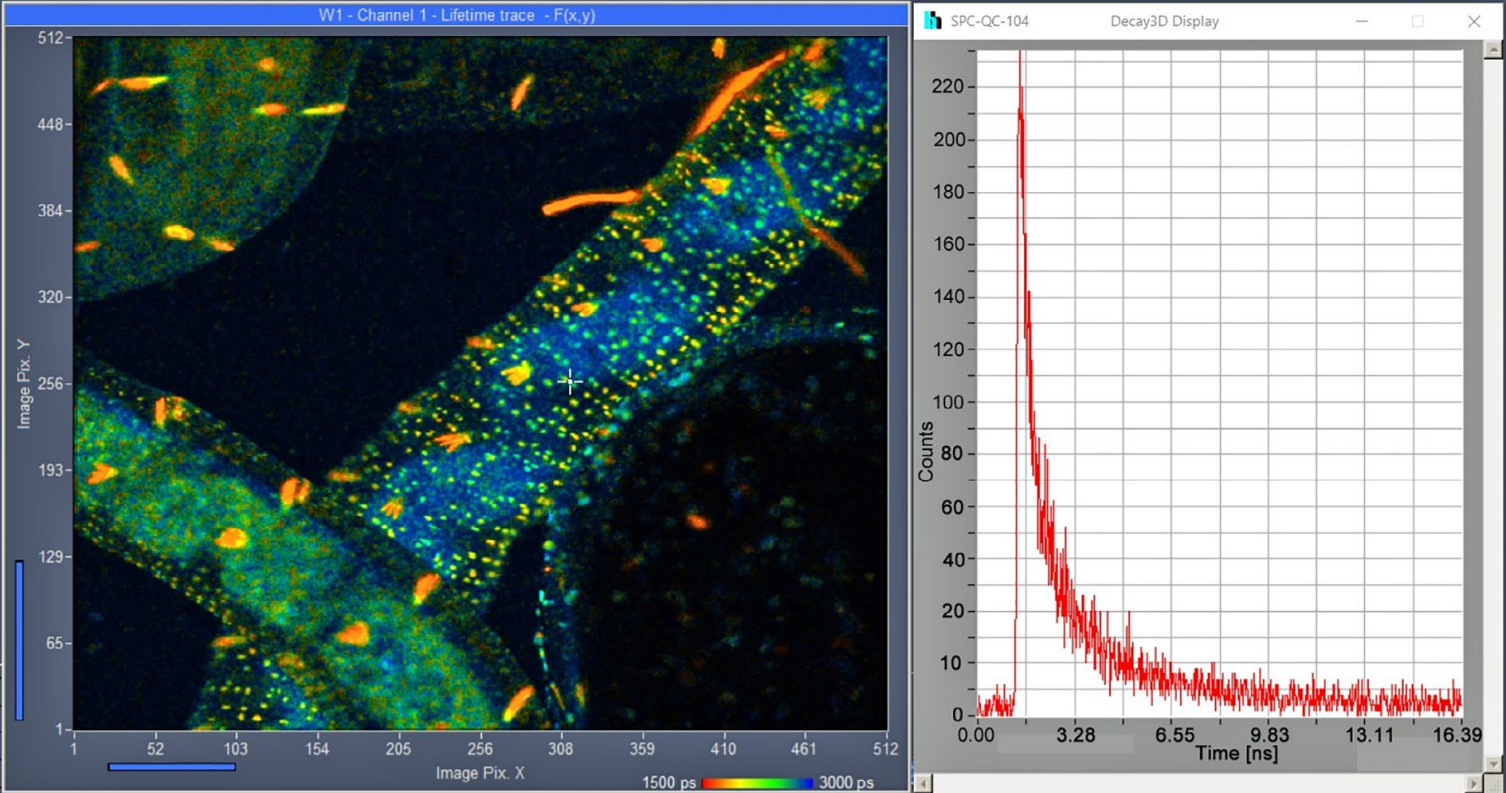
TCSPC FLIM image taken from a live *Enchytraeus albidus* (a little worm). Autofluorescence, image size 1 × 1 mm, 512 × 512 pixels. Excitation wavelength 405 nm, detection wavelength 420 to 650 nm, 1 s acquisition time and 8 MHz average count rate. Decay curve at the cursor position is shown on the right. From [4].

There is currently a run towards faster acquisition of FLIM data. These developments may be technologically impressive [102], but their value as molecular‐imaging techniques is questionable. What is usually forgotten is that a FLIM technique cannot record faster than the sample can deliver the photons. Under these ‘sample‐limited’ conditions the fastest FLIM technique is the one with the highest photon efficiency. Even TCSPC, which is considered to be ‘slow’, is able to record high‐quality FLIM images at acquisition times smaller than one second and below [4]. Standard TCSPC FLIM has been demonstrated for image rates down to 100 ms [103]. The problem of sample‐limited photon rate is mitigated when the image area in the sample is large. The excitation power is then distributed over a larger area [4, 104], massively reducing photodamage effects. An example of fast TCSPC FLIM for a sample of 1 × 1 mm is shown in Figure 17.

A fast TCSPC FLIM technique that distributes the photons from one detector into four TCSPC modules has been described in [105]. The IRF width of the system was 25 ps (FWHM), including the detector, the maximum count rate was 40 × 10^6^ photons/s.

A new version of fast TCSPC FLIM has been introduced recently [106]. The technique reduces data transfer and online data operations by calculating the first moment of decay for every pixel in the TCSPC hardware. The only data that have to be transferred are the first moment of the decay curve and the number of photons in the current pixel. This is achievable even for pixel rates in the MHz region. The charm of the technique is that the instrument can be used for fast FLIM and for ‘normal’ precision FLIM as well [4].

Of course, fast FLIM scanning techniques are limited in speed by the maximum frame rate of the scanner. Wide‐field techniques do not have this limitation. However, apart from the fact that there is no suppression of scattering and out‐of‐focus light, they are still limited by the limited photon rate from the sample. To deal with low photon numbers, fast wide‐field systems sometimes deliver lifetimes only for the whole image, not for the individual pixels. This may be acceptable in special cases, such as Calcium spiking in neurons [44] or contraction of cardiomyocytes [32], but not in the majority of FLIM applications.

### 2.4 FLIM With Special Optical Scanning Techniques

#### 2.4.1 Scanning of Macroscopic Objects

When FLIM techniques are discussed, usually only the combination with microscopy is considered. However, FLIM can also be used on objects with sizes in the cm range. It is often believed that FLIM of such objects has to be performed with a wide‐field technique. However, larger objects can easily be imaged directly in the focal plane of a confocal scanner [4, 77, 104, 107]. The advantage is that out‐of‐focus signals, scattering, glass fluorescence and environment light are suppressed. Examples are shown in Figures 32 and 33, Section 4.7.

#### 2.4.2 Scanning Through Endoscopes

**FIGURE 18:**
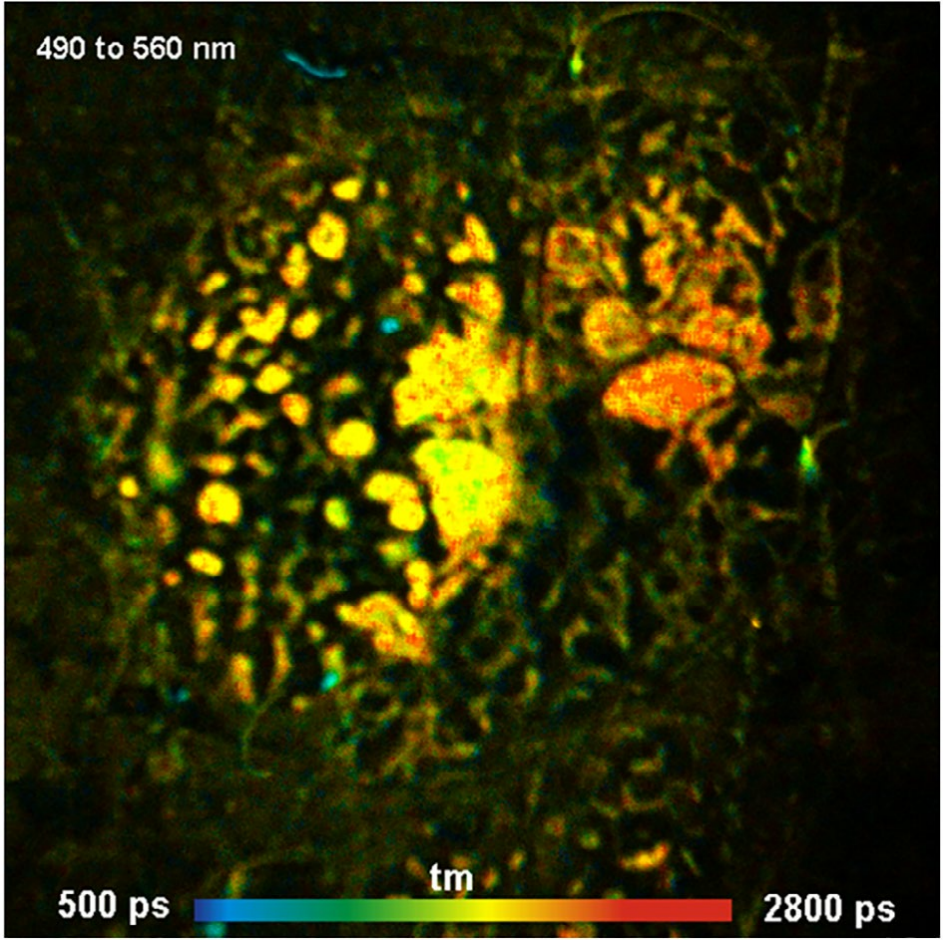
Pigmented skin lesion, scanned in vivo through a lens endoscope. Image size 5 × 5 mm, scanned with 512 × 512 pixels. Becker and Hickl DCS‐120 confocal scanner and Simple‐Tau 152 TCSPC FLIM system. Amplitude‐weighted lifetime of double‐exponential decay, lifetime range 500–2800 ps. Excitation wavelength 405 nm, detection wavelength 480–560 nm, excitation power 50 μW and acquisition time 10 s.

Endoscopes are important for the clinical application of FLIM. In principle, scanning through an endoscope is possible by placing the input image plane of the endoscope in the primary image plane of a confocal scanner [4, 108]. However, there is a caveat. Clinical endoscopes are built with extremely low numerical aperture to achieve a large focus depth. Consequently, the light‐collection efficiency is insufficient for FLIM. Also, the excitation light goes through the same beam path as the light returning from the sample. This creates fluorescence in the glass of the endoscope. Fluorescence in the glass can be so strong that FLIM, especially FLIM of endogenous fluorophores, becomes impossible. This is especially the case for endoscopes with fibre bundles or GRIN lenses where most of the beam path is in the glass. Special fibre bundles made of fused silica mitigate the problem but still restrict the excitation wavelength range to > 450 nm. The problem can be solved by making the endoscope of normal lenses and keeping all intermediate image planes outside the glass. The pinhole of the scanner then suppresses the glass fluorescence [4]. An example is shown in Figure 18. Although the results show that scanning through endoscopes is generally possible—probably even with better image quality than wide‐field imaging—a satisfactory FLIM endoscope for clinical use has not become commonly available yet.

#### 2.4.3 Resonance Scanners

Resonance scanners achieve very fast scanning by operating a galvanometer mirror in a mechanical resonance mode. Resonance scanners can be used with TCSPC FLIM. However, in contrast to common opinion, faster scanning does not result in shorter FLIM acquisition time: The acquisition time depends on the photon rate, not on the rate of scanning. Resonance scanners therefore have not been widely used in FLIM microscopy. They are, however, used in FLIM ophthalmoscopy: The high speed of the resonance scanner prevents the eye from localising the laser spot on the retina and follows it during the scan [4] (Section 4.10).

#### 2.4.4 Bidirectional Scanning

Laser scanning systems normally use a unidirectional scan, with a linear forward portion and a fast flyback when the line is finished. It is often suggested to use a bidirectional scan to exploit also the time when the scanner runs back to the start of the line. Bidirectional scanning has been demonstrated with TCSPC FLIM [4]. However, there is a problem. Unavoidably, the scanner position lags behind the electrical control signal. As a result, the forward portion and the backward portion of the line get dynamically shifted with respect to each other. The shift depends on the scan speed and the scan amplitude. A compensation of the effect is possible only if the scanner runs at a speed below its absolute maximum. The gain in scan speed is therefore smaller than commonly believed.

#### 2.4.5 Acousto‐Optical Scanning

Scanning by acousto‐optical deflectors has been demonstrated by Duocastella et al. [109]. The technique achieves extremely fast scan rates, but the image quality so far does not reach the quality obtained by galvanometer scanners.

#### 2.4.6 Airy Scan

Zeiss introduced a confocal scanning technique in which out‐of‐focus suppression is not performed by a pinhole but by recording an image of the illuminated spot (the Airy disk) by a two‐dimensional detector [54]. The pixels of the detector act as individual pinholes spread over the area of the Airy disk. By aligning and adding the images recorded for the different detector pixels an image with near‐ideal diffraction‐limited resolution is obtained without the need of an extremely small pinhole. The technique can, in principle, be used for FLIM if a SPAD/TDC array or a multichannel PMT is used as a confocal detector. An application of the technique to real FLIM experiments has been described in [53].

### 2.5 Technical Characterisation of FLIM Systems

#### 2.5.1 Time Resolution‐The Instrument Response Function

**FIGURE 19:**
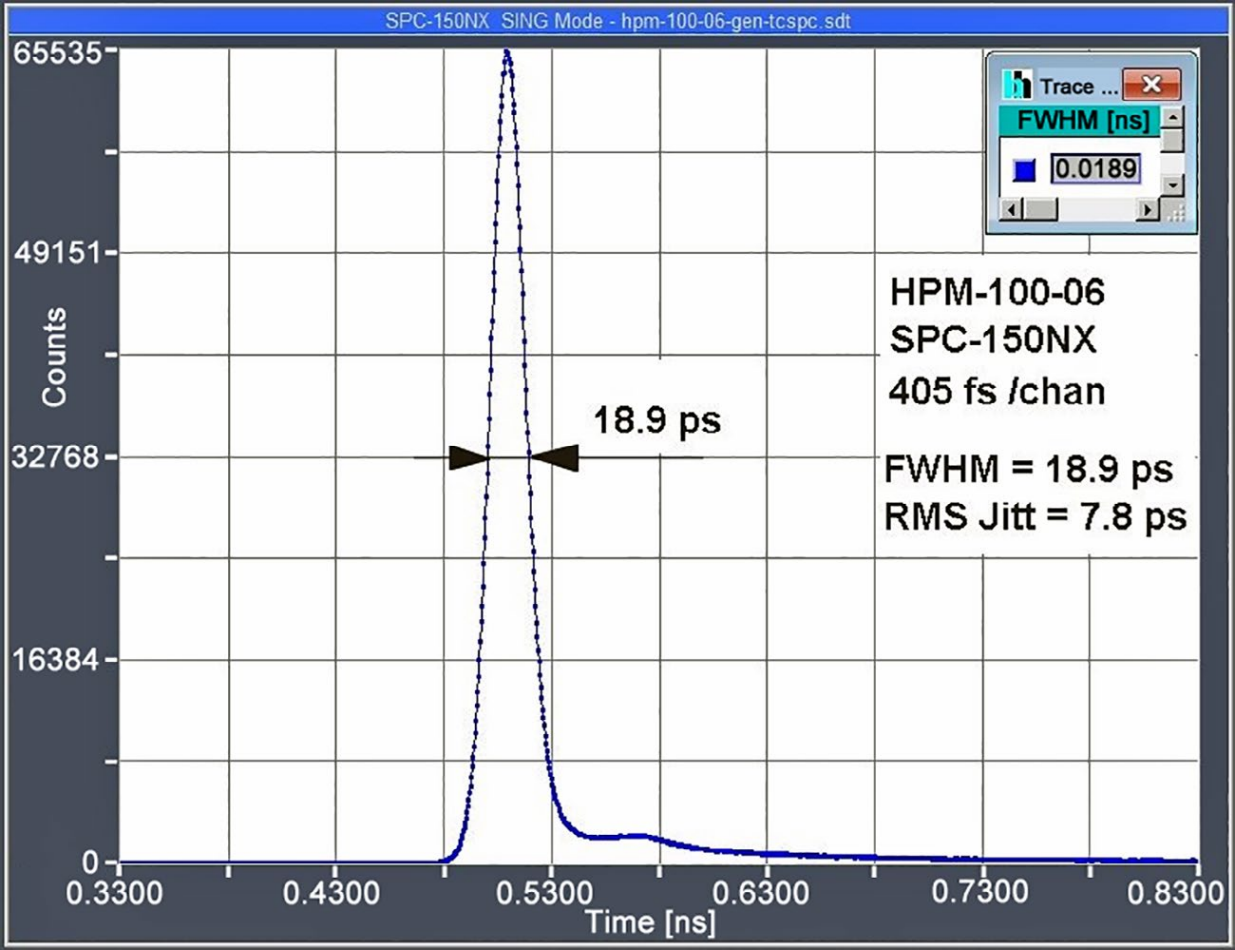
IRF of a fast TCSPC FLIM system. Measured with 120‐fs pulses from femtosecond fibre laser.

The Instrument‐Response Function, or IRF, is the waveform a (time‐domain) FLIM system would record for a sample with infinitely short fluorescence decay time. The IRF is the convolution of the laser pulse width, the transit‐time distribution of the detector, and the temporal jitter of the recording electronics, please see [5, 110]. The width of the IRF is a measure of the time resolution of the system and thus an important quality indicator of a FLIM system. The instrument‐response function of a fast TCSPC FLIM system with a fast hybrid detector is shown in Figure 19. The full width at half maximum is 18.9 ps [4, 12, 13, 14, 15, 111].

There are different parameters to characterise the IRF. The most common one is the ‘Full Width at Half Maximum’, (FWHM). Another one is the ‘Root Mean Square’ (RMS). Calling it an ‘IRF width’ is not correct—technically it should be called the ‘RMS Timing Jitter’ of the system. For a Gaussian IRF, the RMS timing jitter is 1/2.3 of the FWHM of the IRF. It is often used by manufacturers to pretend a higher time resolution than their system actually has.

Other definitions of the time resolution, such as time‐channel width, the minimum lifetime that can be resolved or the standard deviation of the lifetime are completely misleading. According to Nyquist's theory, the time‐channel width has to be several times shorter than the IRF width to avoid sampling artefacts. Reducing it further does not increase the effective time resolution. The minimum resolved lifetime depends on the number of photons in the pixels of the image, and the standard deviation not only depends on the photon number but also on the lifetime itself. These parameters are thus useless to characterise the resolution of the FLIM system. An extremely misleading definition is the lifetime accuracy that is obtained when the data are averaged over the entire image. Averaging over the entire image results in extremely high photon number and thus in ridiculously low standard deviation of the lifetime.

The definitions described above can be extended to time‐gating techniques. The IRF is then determined by the shape and the width of the gate pulse. The minimum gate‐shift interval is the equivalent of the time‐channel width of TCSPC. To obtain a maximum resolution, it has to be shorter than the gate width, but by itself, it does not define the time resolution of the system.

For frequency‐domain systems, the definition of an ‘IRF’ is more complicated. The phases and the amplitudes of the phasors are not visibly connected to the IRF width in the time domain. It is therefore often believed that the IRF width has no influence on phasor analysis. This is, however, not the case. Even if the effect of the temporal IRF is correctly taken into account in the phasor calculation (Section 2.6) the timing jitter of the individual photons is present as phase and amplitude jitter in the phasors of the individual pixels. It therefore increases the standard deviation of the phasors and broadens the phasor clusters in the phasor plot. The frequency‐domain equivalent of the time‐domain IRF would be a ‘System Bandwidth’. However, to the best of our knowledge, there is no such parameter that would be commonly used in the frequency domain.

#### 2.5.2 Timing Stability

In many instances, *timing stability* (including low‐frequency timing wobble) is as important as time resolution. Even in standard fluorescence‐decay measurements timing drift matters: Timing shift between the fluorescence recording and a related IRF recording transfers directly into the measured fluorescence lifetime. Moreover, there are setups where an exact IRF is difficult to record. Typical examples are confocal microscopes, where dichroic mirrors and filters block the detection path for the excitation wavelength. In these cases, it is important that a stable IRF is maintained over a long period of time. TCSPC FLIM reaches a specified timing stability of better than 5 ps [4].

#### 2.5.3 Statistical Accuracy

The statistical accuracy, or the standard deviation of the measured fluorescence lifetime can be derived from the first moment of the photon distribution [4, 16, 34, 103, 112]. For decay data recorded with a sufficiently short IRF, negligible background, and within an observation‐time interval covering the entire decay the relative standard deviation is the reciprocal square root, $1/\sqrt{N}$, of the number of photons, *N* [58, 103]. The relative standard deviation of an MLE fit comes close to this, and so does the standard deviation of a phasor.

This is a remarkable result in several respects. First, the signal‐to‐noise ratio for the pixel lifetime is the same as for the *pixel intensity*. This invalidates the widespread opinion that FLIM needs more photons (and thus more acquisition time) than steady‐state imaging. Second, the signal‐to‐noise ratio depends *only* on N. In particular, is does not depend on the number of time channels into which the fluorescence decay is recorded. In other words, you can increase the number of time channels to reduce sampling artefacts without compromising the signal‐to‐noise ratio. Third, because the SNR depends on N only, the only way to increase the lifetime accuracy is to increase N. That means you either have to increase the count rate—which is prevented by sample degradation, increase the acquisition time or decrease the number of pixels—which you normally don't want—or use overlapping binning in the lifetime analysis [110], see Section 2.6.

There is no measurement or data processing technique which can exceed the magic limit of $1/\sqrt{N}$. There are, however, a number of effects that lead to *lower* accuracy. The most common one is background counts. Background counts usually come from insufficient shielding from room light but may also originate from the detector after pulsing. A decrease in accuracy also occurs if the tail of the decay curve is not in the observation‐time interval or if the IRF width is in the same order as the fluorescence lifetime. Please see [5, 58] for details.

The relative accuracy for a given number of photons (or the relative number of photons for a given accuracy) under ideal and non‐ideal conditions is quantified by a ‘Photon Efficiency’. The Photon Efficiency is the ratio of the number of photons needed under ideal conditions to the number of photons needed under real conditions. Theoretical considerations on the photon efficiency of different FLIM techniques can be found in [38, 39, 40].

With enough photons, the lifetime of a single‐exponential decay can, in principle be detected down to values much shorter than the IRF width. However, the accuracy decreases (or the number of required photons increases) rapidly when the decay time becomes shorter than the IRF width.

The number of photons required for a given accuracy increases dramatically when multi‐exponential decay functions have to be split into single‐exponential components [5]. An attempt at an estimation of the photon number has been made by Köllner and Wolfrum [58]. The result was that 400,000 photons would be needed for resolving a double‐exponential decay. However, the example given in [58] assumes an extremely unfavourable composition of the decay function. Under typical FLIM conditions, with the component lifetimes apart by a factor of 5 or more, double‐exponential analysis by MLE techniques is possible with 1000 photons, and very satisfactory results can be obtained from 5000 photons. These numbers can easily be reached by moderate binning radius in the lifetime analysis (Section 2.6.1.5) or even in single pixels. The required photon number can be reduced if a priori knowledge of the component lifetimes is available.

#### 2.5.4 Non‐Ideal Effects of FLIM

‘Counting loss’ is the loss of a photon in the data processing time (dead time) of a previous one. Counting loss causes a nonlinearity in the intensity scale of the FLIM image. This is normally not a problem because the intensity is rarely used as a source of information in FLIM experiments. In the past few years counting loss of TCSPC has been dramatically reduced by faster time‐conversion principles [4] and by deriving the pixel intensities from a fast counter that bypasses the TCSPC timing electronics [113].

More severe is the influence of the ‘Pile‐Up’ effect. Pile‐Up is the possible detection—and loss—of a second photon in the same excitation pulse period as a previous one. As a consequence, an optical waveform recorded by TCSPC gets distorted when the count rate becomes too high. Extreme pile‐up situations are described in [114], ways to deal with moderate pile‐up in [52, 115]. However, the best way to avoid pile‐up distortion is to keep the excitation pulse rate as high as possible and the detection rate at reasonable level. It is also possible—yet not often needed—to split the detection signals into several detection channels [105] or into a larger number of elements of a SPAD array [49].

The pile‐up effect is often used as an argument against TCSPC and, especially, TCSPC FLIM. It is often stated that the count rate of TCSPC and TCSPC FLIM must not exceed 0.1% of the excitation pulse rate. This is wrong. The correct pile‐up limit is 0.1× the excitation pulse rate, not 0.1% of it. The correct value has been calculated by O'Connor and Phillips [66] and by Becker [4, 16]. The mistake appeared in papers later than 1985 and probably stems from a typo in one of the later publications.

With the correct value of the pile‐up limit, the maximum count rate for 80 MHz laser repetition rate is 8 MHz (8 × 10^6^ photons per second). This is 100 times more than commonly believed and significantly more than typical FLIM samples can deliver without photo‐induced changes in their molecular structure. It is probably a singular case in technical history that a typo has impeded the application of a technique over half a century.

The fact that the technical literature mentions systematic errors only for TCSPC does not mean that other techniques are free of such errors. At high count rates, all detectors show saturation effects, nonlinearity and timing shifts. The micro‐channels of multichannel plates in image intensifiers do not fully recharge between subsequent photons, the reverse voltage of SPADs does not cleanly recover, and the output pulse amplitude of PMTs and hybrid detectors breaks down under the load of the photocurrent. The size of these effects depends on the details of the detector design and is thus not entirely predictable. A common mistake is to assume that a device based on single‐photon timing (such as TCSPC) is free of counting‐loss errors if only the dead time is short enough. If two photons come closer to each other than the width of the single‐photon response of the detector/discriminator combination they cannot be distinguished. Consequently, they are counted as one photon. The result is a nonlinear distortion around the maximum of the recorded decay curves. The associated loss of photons can be seen in high‐count rate data. It should be noted, however, that the effects described here happen at count rates which rarely occur in real FLIM experiments.

FLIM techniques using detection in different time gates or at different phase angles and recording the corresponding data sequentially are prone to photobleaching artefacts. If the sample bleaches during the measurement the intensities in the time windows and the intensities for different phases become impaired and the lifetime becomes wrong. Similar errors are induced if there are changes by motion or physiological effects in the sample. To a certain degree, such errors can be reduced by permutation of the measurement sequence, but the remaining artefacts are hard to quantify.

### 2.6 Data Analysis

#### 2.6.1 Phasor Analysis

Phasor Analysis and the Phasor Plot are based on the analysis of the decay data in the frequency domain. A transformation of the decay data, *n*(*t*), into the frequency domain delivers a complex function of frequency, *G*(ω) + i*S* (ω) with

(1) $$G\left(\omega \right)=\frac{1}{N}\sum \cos\left(\mathrm{\omega t}\right)n\left(t\right)\;\text{and}\;S\left(\omega \right)=\frac{1}{N}\sum \sin\left(\mathrm{\omega t}\right)n\left(t\right)$$

As it turns out, a good representation of the decay function is obtained already if only *G* and *S* at the fundamental repetition frequency (the first Fourier component) are used. The decay function is then described by just two numbers, *G* and *S*, for ω = 2π/*T*, where *T* is signal period of the fluorescence signal. The data points, *G* + i*S*, can also be expressed by the magnitude, *M*, and the phase, *φ*, in the plane of complex numbers:

(2) $$M=\sqrt{G^{2}+S^{2}}\;\varphi =\arctan S/G$$

Time‐domain systems calculate M and φ by the procedure described above, and frequency‐domain FLIM systems deliver M and φ directly. In both cases, M and φ represent the measurement signal, not the real parameters of the fluorescence decay. M and φ still must be corrected by the *M*_irf_ and φ_irf_ values of the instrument‐response function (IRF). The corresponding transformation is a simple multiplication in the plane of complex numbers. The fact that the transformation is mathematically easy has led to the misconception that the phasor analysis does not need an IRF. This is wrong—the effects of a wrong or neglected IRF are the same as in time‐domain analysis.

In the IRF‐corrected form, the representation of a decay curve in the M and φ plane is called a ‘phasor’, and a density plot of the phasors of all pixels of an image or an image area is called a ‘phasor plot’ [42, 60].

**FIGURE 20:**
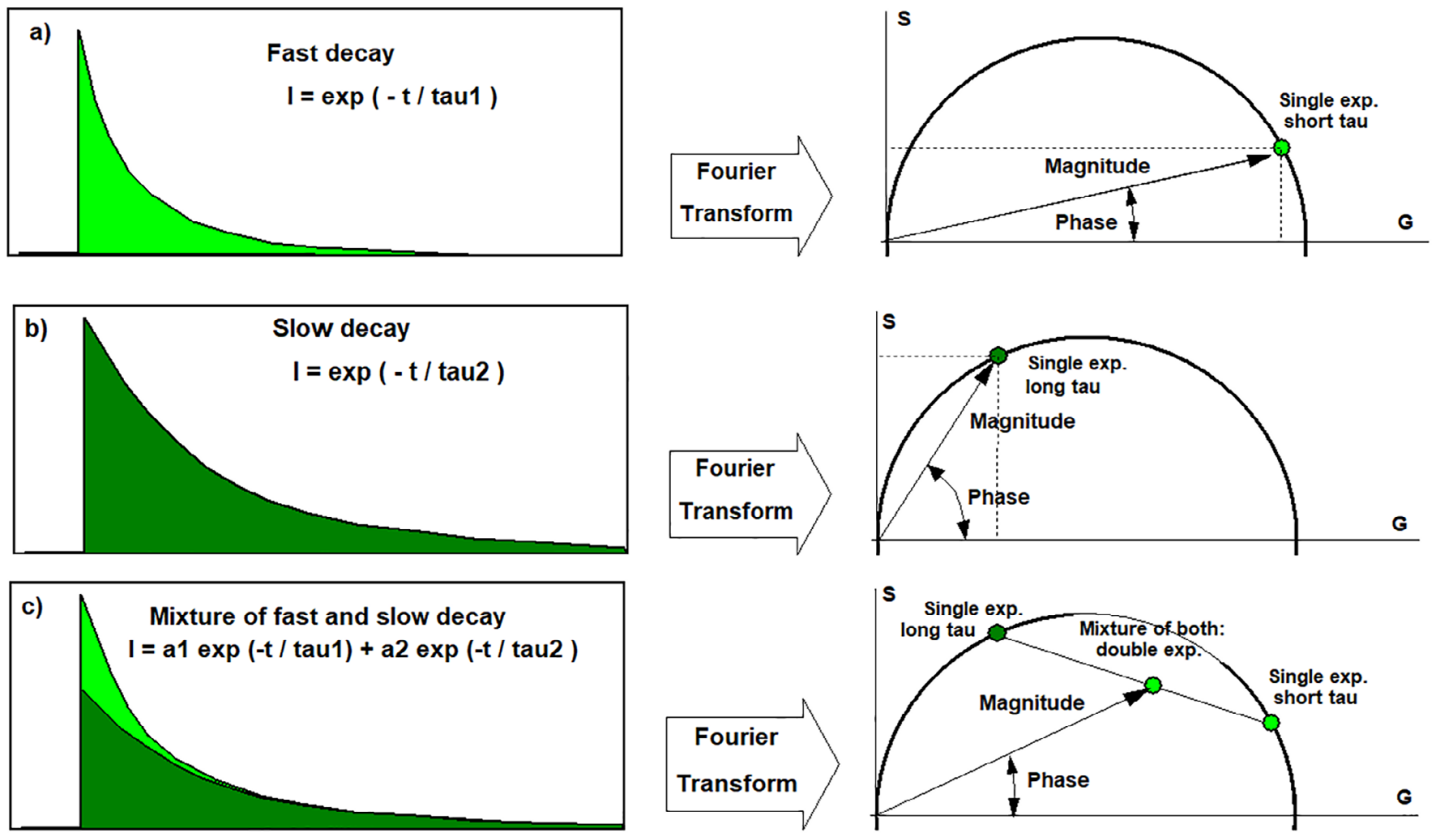
Relationship between decay functions in the time domain (left) and phasors in the frequency domain (right).

The relationship between different decay curves and their phasors is illustrated in Figure 20. The phasors of single‐exponential decays are located on a semi‐circle in the *G*‐*S* plane. The phasor of a fast decay (a) is located on the right, at low phase and large magnitude. The phasor of a slow decay (b) in located farther left, at larger phase and lower magnitude. The phasor of a double‐exponential decay (c) is a linear combination of the phasors of the decay components. It is located inside the semicircle.

**FIGURE 21:**
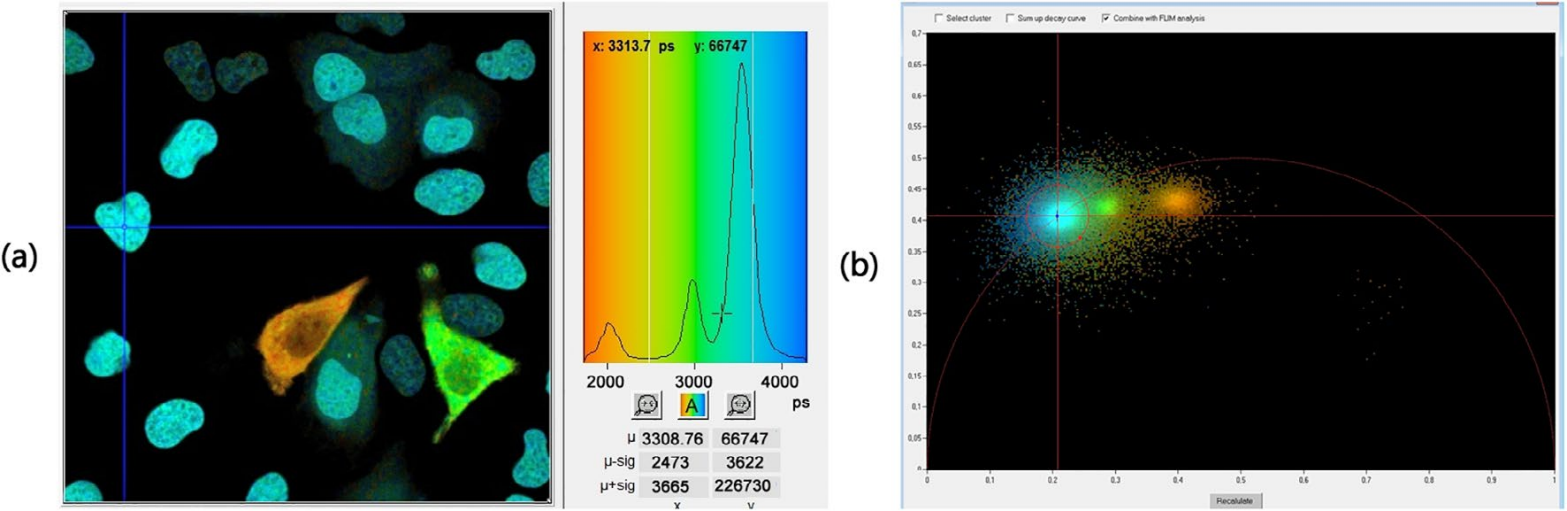
Time‐domain lifetime image (a) and corresponding phasor plot (b). Pixels with similar decay profiles form clusters in the phasor plot. Image size: 100 × 100 μm.

A fluorescence‐lifetime image and the corresponding phasor plot are shown in Figure 21. The pixels of objects with similar decay signatures in the FLIM image (a) form clusters in the phasor plot (b).

Single‐exponential decay times can be derived from the phasor plot by centroiding the corresponding phasor clusters, that is, the blue one in Figure 21b. Double‐exponential decays require at least one of the component phasors to be known.

It is a common misconception that phasor analysis generally needs less photons than time‐domain analysis. This is not the case. The accuracy of a single phasor is not higher than the lifetime for a single pixel in time‐domain analysis. The reason for high accuracy is the centroiding, that is, averaging the phasors of a large number of pixels. With binning or image segmentation time‐domain analysis achieves similar accuracy. Moreover, phasor analysis of a multi‐exponential decay function uses a priori knowledge of the phasors of the pure decay components. This is equivalent to time‐domain analysis with fixed component lifetimes. Under these conditions, time‐domain analysis delivers similar accuracy as frequency‐domain analysis.

#### 2.6.2 Time‐Domain Analysis

Time‐domain techniques deliver an array of temporal data over the pixels of the image. As for phasor data, the temporal data in the pixels do not directly represent the shape of the fluorescence decay. Instead, they are the convolution of the instrument response function with the real decay profiles. The convolution integral [110] can easily be calculated in forward direction but, in the general case, cannot be calculated back. A simple solution is to use moments for lifetime calculation [5, 34]. The first moment is easy to calculate and—under ideal conditions—delivers a fluorescence lifetime with the minimum possible standard deviation [4]. However, it delivers only a single‐exponential approximation of the lifetime and is prone to systematic effects if there is a baseline offset or the fluorescence does not decay completely in the observation time interval. Time‐domain analysis therefore uses a fit procedure. A model function is convoluted with the IRF, the result is compared with the data, and the model parameters are optimised until the best fit is obtained [110].

##### 2.6.2.1 Least‐Square Fit

**FIGURE 22:**
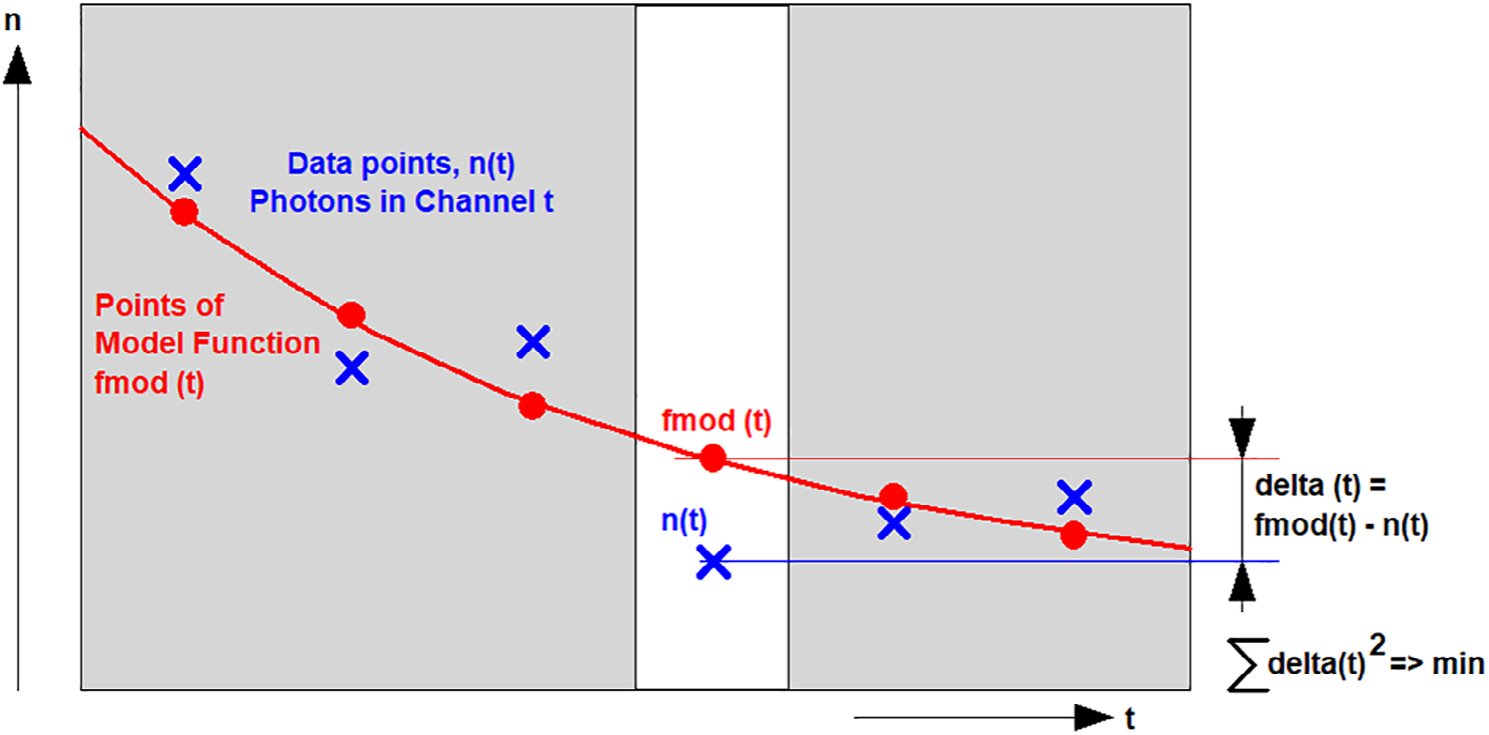
Least‐square fit procedure.

When it comes to fitting users usually think of the traditional least‐square fit. The procedure is shown in Figure 22. The differences between the data points and the corresponding points of the model function are calculated, squared and summed up for the entire curve. Then parameters of the model function are optimised until the error‐square sum is at minimum.

Unfortunately, the procedure has a flaw. The problem lies in the photon statistics. The photon numbers in the data points are Poisson‐distributed. That means the noise in the decay data depends on the photon number in the particular time channel: the (absolute) variance of the photon numbers in the time channel is the square of the photon number. Therefore, a correct result is only obtained by weighting the residuals. The weight for data points with low photon number must be higher than that of the points with higher photon number. Worse, the weight for data points with *n* = 0 had to be infinite—a practical impossibility [5, 92, 110]. As a result, the fluorescence lifetime is determined too short for decay curves with low photon number.

##### 2.6.2.2 Maximum‐Likelihood Estimation

**FIGURE 23:**
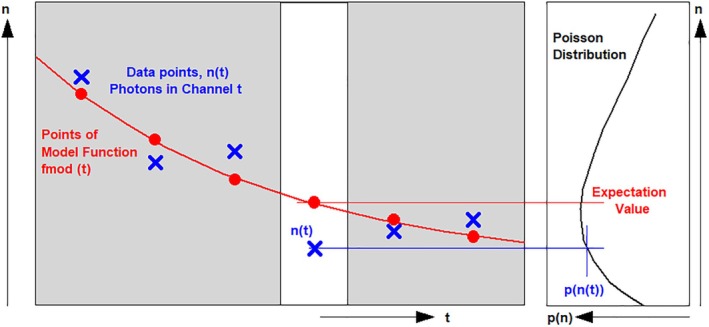
Principle of MLE fit. For each point of the model function, *f*_mod_(*t*), a Poisson distribution is associated. The function delivers a probability *p*(*n*(*t*)), that a given data point, *n*(*t*) fits to the corresponding point of the model function. The product of *p*(*n*(*t*)) over all time channels is used for optimising the parameters of the model function.

MLE is based on calculating the *probability* that the values of the model function correctly represent the data points of the decay function. The principle is illustrated in Figure 23. To each point of the model function, *f*_mod_(*t*), a Poisson distribution with an expectation value equal to *E* = *f*_mod_(*t*), is associated, see Figure 23, right. From this distribution, the probability, *p*(*n*(*t*)), is calculated. The probability *p*(*n*(*t*)) tells how likely it is that the point of the model function is a correct representation of the data point. *p*(*n*(*t*)) is calculated for all time channels, that is, for all pairs of data points and model‐function points. The product of these probabilities is the probability that the model function represents the data. The parameters of the model functions are then optimised until the maximum probability is obtained.

The MLE fit has no problem with data points of low photon number or even a photon number of zero. The Poisson distribution associates correct probabilities to all these situations, and the product of the probabilities correctly describes the quality of the fit. Consequently, there is no bias toward shorter lifetime, as it occurs for the weighted least square fit. As a side effect, MLE yields more reliable parameters for the components of multi‐dimensional decay functions. This is important for molecular imaging applications, where the information is often more in the amplitudes and lifetimes of the decay components than in the average decay time. For a detailed description, please see [4, 5, 110].

##### 2.6.2.3 Computation Speed

Time‐domain FLIM analysis by fit procedures is computationally intensive. Therefore, it has a reputation for being slow. There have been various attempts to replace the fit procedure with faster algorithms [102], some of which are approaches revived from the pre‐computer era. Most of these procedures sacrifice the multi‐exponential decay capabilities of the fit procedures or deliver decay data at an accuracy far from the theoretical maximum of N^−1/2^. With the introduction of Graphics Processing Units (GPUs) such compromises are no longer necessary. On a GPU, the deconvolution and fit procedure of many pixels runs in parallel, resulting in extremely short computation times. For images with 512 × 512 pixels and 1024 time channels, computation times in the range of 0.5–5 s are typical [110].

##### 2.6.2.4 Global Fit

The short computation time makes it possible to run a ‘Global Fit’ on the FLIM data. A global fit makes use of the fact that often one or several lifetimes in a multiexponential decay do not change over the image area. A global fit runs several analysis cycles with different values of these lifetimes and optimises their values until the best fit for the entire image is obtained. Global fitting can significantly improve the accuracy of FRET analysis and metabolic‐FLIM analysis [116].

##### 2.6.2.5 Intelligent Binning

In time‐domain analysis, there is no automatic clustering or centroiding function for the decay data as it exists for the phasors in the frequency domain. Therefore, time‐domain analysis makes use of ‘Intelligent Binning’: Microscopy images are normally over‐sampled. Typically, the point‐spread function should be sampled by 5 × 5 or more pixels [117]. The decay data within this area are, of course, highly correlated. There is no point in analysing them independently. Therefore, time‐domain analysis uses an overlapping binning recipe. For every pixel of the image, the decay information is not only taken from this pixel, but also from pixels within a certain radius around it. The binning radius is adjusted to the radius of the point spread function, and decay analysis is performed on the binned data. The result is a substantial increase in the effective photon number and, consequently, in the accuracy of the decay parameters obtained. The total number of pixels in the image—and thus the spatial oversampling factor—is not changed. A detailed description and examples can be found in [4, 5].

##### 2.6.2.6 Fit Quality Indicators

The quality of the fit of time‐domain analysis is indicated by the ‘Residuals’ and the ‘Reduced Chi‐Square’ (χ^2^) [66, 110]. The residuals are the differences between the values of the model function (convoluted with the IRF), *f*_mod_(*t*), and the data points of the recorded decay curve, *n*(*t*), divided by the square root of *f*_mod_(*t*):

(3) $$R\left(t\right)=\frac{f_{\mathrm{mod}}\left(t\right)-n\left(t\right)}{\sqrt{f_{\mathrm{mod}}\left(t\right)}}$$

The background of this expression is as follows. Provided the model function reproduces the shape of decay function properly the differences between *f*_mod_(*t*) and *n*(*t*) can be considered the ‘noise’ in the data. The noise comes from the photon statistics. The average noise amplitude is statistically defined and is $\sqrt{n}$. If the model function is correct the denominator of *R*(*t*) is equal to the expectation value of the photon number, *n*(*t*). That means the residuals should not depend on the photon number in the respective time channel of the decay function, and vary randomly with a ‘Sigma’ of one. Any systematic deviation of the convoluted model function *f*_mod_(*t*) from the photon data, *n*(*t*) causes systematic wobble in the residuals. Examples of residuals can be seen under the decay curves in Figures 25, 32 and 37.

There is a feature of the residuals which sometimes causes confusion. When the photon number gets very large the amplitude of the systematic variations increases linearly with the photon number, n, whereas the random variations increase only with $\sqrt{n}$. The residuals can therefore not be used to compare data of largely different photon number.

The other indicator, χ^2^, is calculated by

(4) $$\chi^{2}=\sum_{k}\frac{{\left(f_{\mathrm{mod}}\left(t\right)-n\left(t\right)\right)}^{2}}{k\left(n\left(t\right)+1\right)}$$

*f*_mod_(*t*) is the (convoluted) model function, *n*(*t*) is the photon number in the time channel *t*, and *k* is the number of time channels. The background of the formula is similar as for the residuals. The numerator is the square of the difference between the model function and the photon number in the corresponding time channel. For large *n*, the denominator, *n*(*t*) + 1, is the square of the expected noise in the photon number. Consequently, an ideal fit should deliver a χ^2^ of approximately one.

A confusing feature of χ^2^ is that it increases for large photon numbers. The reason is the same as for the residuals: The amplitude of systematic variations increases linearly with the photon number, *n*, whereas the random variations increase only with $\sqrt{n}$. Therefore, χ^2^ increases with the photon number. It can only be used to compare the fit quality for different models and different fit parameters within a given set of decay data but not for different data with largely different photon numbers.

#### 2.6.3 Deep Learning: Data Analysis by Artificial Intelligence

**FIGURE 24:**
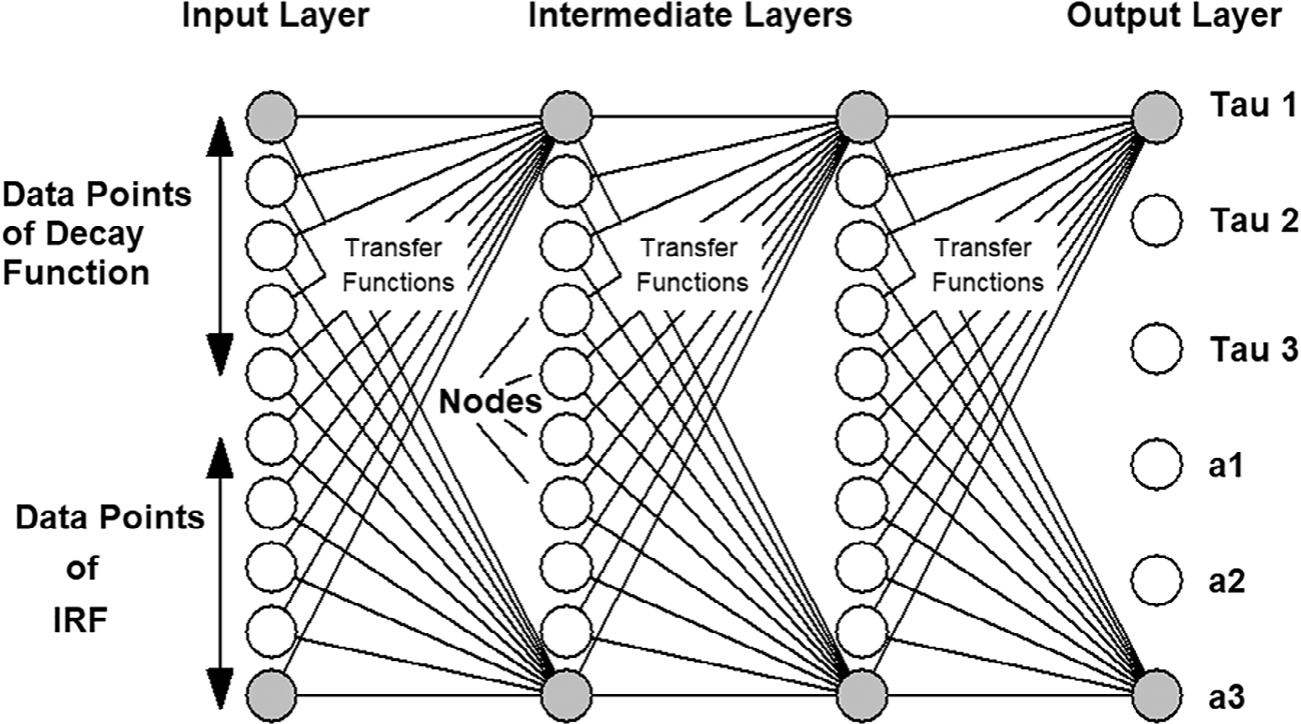
Decay analysis by a neural network. Interconnections are shown for the first and last node (grey) of each layer only. Two intermediate layers are shown. There can be more, depending on the complexity of the problem.

Data analysis by artificial intelligence (AI) is based on a neural network [118]. A neural network has a number of input variables, a number of successive ‘layers’, and a number of output variables. The first layer is the ‘input’ layer. It is followed by a number of ‘intermediate’ layers. The last layer is the ‘output layer’ which contains the output variables (Figure 24). The variables in the nodes of each layer depend on the values of the variables in the nodes of the previous layer. In the simplest case, the variables are linear combinations of the variables in the nodes of the previous layer.

Initially, a neural network is entirely undefined in the transfer of data from the input layer to the output layer. The interconnection between the input and the output has to be defined by training. The process is also called ‘deep learning’. It means the network is fed with measured or simulated data for which the output data are known. For each training data set, an optimisation algorithm determines the parameters of the transfer functions from the nodes of one layer to the next. After repeating this procedure for a large number of data sets a set of transfer function parameters is found which satisfies the input–output relations for all training data sets.

Once the parameters of the transfer functions are defined unknown data can be sent though the network and the outputs be expected to correct solutions to the particular input data set. For decay analysis, the nodes of the input layer would be the data points of the measured fluorescence decay and the data points of the IRF, the nodes of the output layer the lifetimes and amplitudes of the decay components (Figure 24). Of course, the output nodes can also contain other parameters than decay parameters, especially such which relate directly to clinical diagnosis. A review of AI‐based FLIM analysis can be found in [119].

As fascinating as AI‐based analysis may appear, it is not free of pitfalls. First, AI analysis cannot be better than the training data are. If, for example, the training data are based on curve fitting of real measurement data, possible fit artefacts—by deficiencies of the fit procedure or by user mistakes—are transferred into the neural network. Data processed by this network then contain the same artefacts. If training is based on simulated data the results become wrong if the simulation neglects one or several essential parameters, such as counting background, incomplete decay, or additional decay components. Moreover, the common opinion that AI analysis is generally faster than a fit procedure [119, 120] is not correct. The computational effort of AI processing is enormous, especially in the training phase. But even for routine data analysis, it is high, especially when larger numbers of time channels and pixels are used. Therefore, AI algorithms normally use GPU processing. Of course, this makes them faster than a fit algorithm running purely on the CPU. However, state‐of‐the‐art time‐domain analysis uses GPU processing as well. The computation speed is then at least as high as for AI‐based procedures [110].

## 3 Standard Molecular‐Imaging Applications

### 3.1 Molecular‐Environment Parameters

Superficially considered, the measurement of molecular environment parameters is the most straightforward FLIM application. There is a large number of fluorophores which change their fluorescence lifetime depending on the pH, the concentration of physiologically or environmentally relevant ions, glucose concentration, local viscosity and others [19, 121, 122, 123]. The lifetime changes are usually large, and easy to measure with all lifetime imaging techniques described in this article. The tricky part is the calibration of the lifetime change of the dyes as a function of the measured cell parameter. The calibration must be performed in the real biological environment and is thus not easy. Moreover, it is often desirable to detect several molecular parameters simultaneously and their mutual dependence. This requires detection in several spectral channels, multi‐wavelength FLIM, or excitation wavelength multiplexing.

### 3.2 Calcium Transients

Things become difficult when the measured cell parameter changes on a fast time scale. A typical example is Calcium‐ion concentration in neurons. Ca^2+^ changes (calcium transients) occur within a period of about 50 ms. It is often attempted to use fast FLIM techniques to record such signals. However, this faces the usual problems of low photon budget and, if the laser power is increased, photobleaching [32]. One solution is to integrate the decay over the entire image, giving up spatial resolution. The other is to trigger the lifetime change electrically and use TCSPC FLIM with triggered accumulation [88, 89] (Figure 15).

### 3.3 Membrane Potential

**FIGURE 25:**
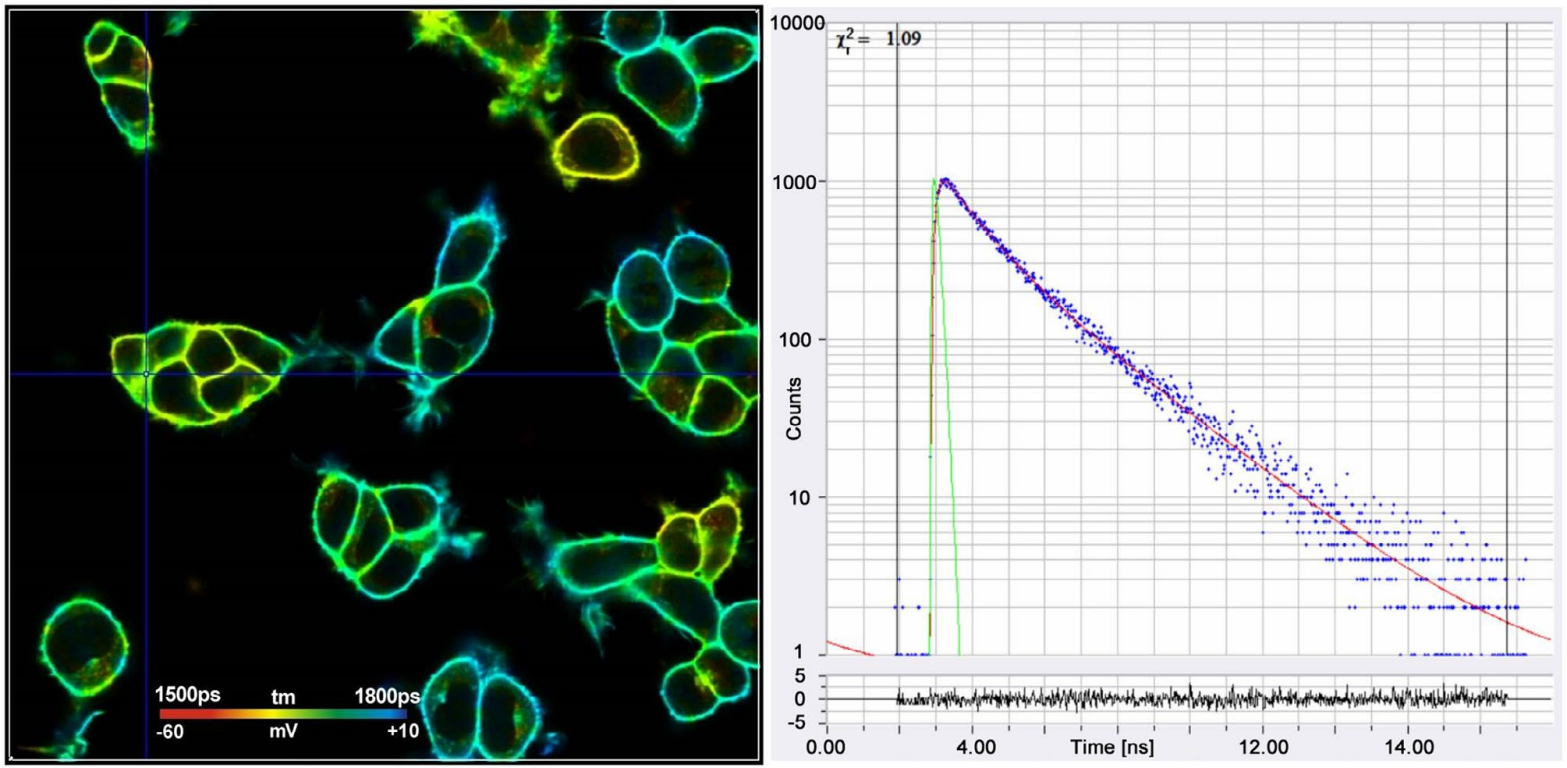
Membrane‐potential measurement by FLIM. Image size: 150 × 150 μm. Lifetime image with voltage scale and decay curve at an arbitrary cursor position. Residuals (Section 2.6.1.6) are shown under the decay curve, fit‐quality indicator χ^2^ upper right of the decay curve.

Cells have concentration gradients of ions across their membranes. These gradients produce a voltage over the membrane which is characteristic of several fundamental cellular processes [124]. Techniques for precise measurement of membrane potentials are rare. A promising solution is FLIM with a voltage‐sensitive dye [125, 126]. An estimation of the number of photons needed to reach a given voltage accuracy with VoltageFluor VF2.1.Cl and a high‐resolution membrane‐voltage image of HEK cells are given in [127]. An example is shown in Figure 25.

### 3.4 Förster Resonance Energy Transfer

**FIGURE 26:**
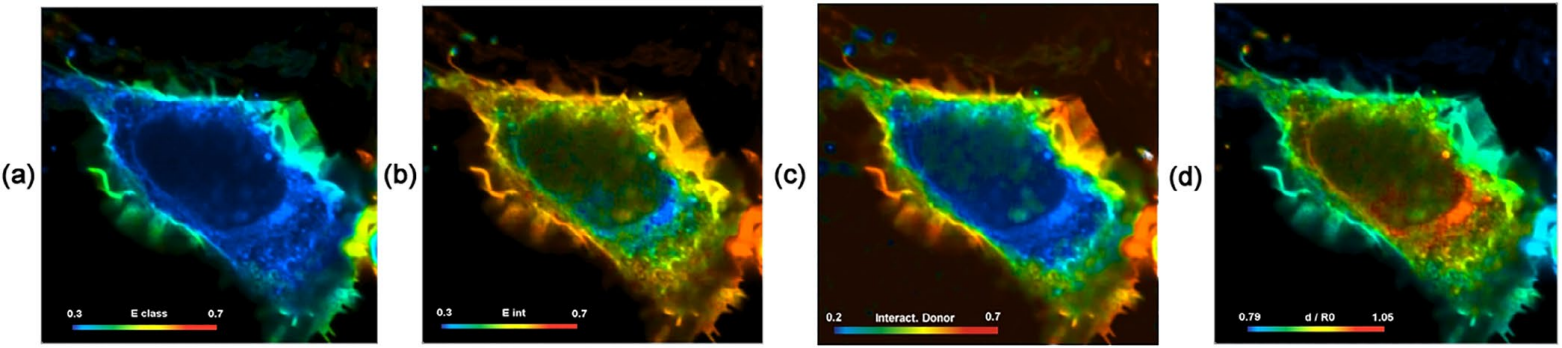
FRET Measurement in a life cell. Let to right: Classic FRET efficiency, FRET efficiency of interacting donor, amount of interacting donor and ratio of donor‐acceptor distance to Förster radius. Double‐exponential FRET technique [132], Image size: 20 × 20 μm, resolution 512 × 512 pixels. Zeiss LSM‐880 with Becker and Hickl SPC‐152 FLIM system, SPCImage NG data analysis.

Förster resonance energy transfer (FRET) [10, 11] is commonly used to probe protein interaction and protein structure in biological systems. The proteins under investigation are labelled with a donor and an acceptor. The excitation energy is absorbed by the donor. It then transfers to the acceptor, and is emitted via the emission band of the acceptor. The energy‐transfer rate increases with the 6th power of the reciprocal distance. FRET measurement by intensity‐based techniques [128] is possible but difficult to calibrate, and does not deliver quantitative results. Apart from difficult calibration for donor bleedthrough and directly excited acceptor, intensity‐based FRET does not distinguish between the effects of the donor–acceptor distance and the amount of interacting donors. These calibrations are avoided by FLIM–FRET: The donor lifetime predictably decreases with increasing FRET intensity [16, 68, 129, 130]. However, traditional single‐exponential decay measurement still has the problem that distance and interacting donor fraction are not distinguished [131]. Quantitative results can only be obtained by accurate measurement of the donor decay curve by TCSPC FLIM and double‐exponential decay analysis [4, 131, 132]. The fast decay component is the decay function of the interacting donor, and the slow component is the decay function of the non‐interacting donor. From the amplitudes and the lifetimes of the components can be calculated the classic FRET efficiency, the true FRET efficiency of the interacting donor, the relative amount of interacting donor and (with the known Förster radius, *r*_0_) the donor–acceptor distance. By taking the lifetime of the non‐interacting donor function as a reference, the technique even becomes free of external calibration. An example is shown in Figure 26. An overview of the FLIM FRET literature is available in [4], chapter ‘Förster Resonance Energy Transfer (FRET)’.

It is sometimes criticised that the double‐exponential model is a simplification in that the interacting‐donor decay should actually be a distribution of components of different lifetimes. This is due to the fact that there is probably a distribution in the donor–acceptor distance and in the donor–acceptor angle. However, the statistics of available FLIM FRET data (including our own ones) are insufficient to distinguish between the two models. We therefore consider it reasonable to stay with the relatively simple double‐exponential model until its capabilities are fully exhausted.

### 3.5 Anisotropy‐Decay Imaging

When fluorophores are excited by polarised light the polarisation of the fluorescence is smaller than that of the laser beam. The depolarisation in the fluorescence emission is described by a parameter called ‘Anisotropy’ [4, 133]. Because the molecules can rotate the anisotropy decreases after the excitation pulse. Typical anisotropy‐decay times range from a few 100 ps for small molecules in low‐viscosity solvents to microseconds for fluorophores bound to biological macromolecules. The anisotropy‐decay time is therefore another indicator of the local molecular environment. Anisotropy‐decay measurement requires the fluorescence to be recorded in two polarisation channels, parallel and perpendicular to the polarisation of the excitation. Of course, the anisotropy decay, *r*(*t*), overlays with the fluorescence decay. It has therefore to be calculated from the difference of the two signals, parallel and perpendicular to the laser, *I*_para_(*t*) and *I*_perp_(*t*).

$$r\left(t\right)=\left(I_{\text{para}}\left(t\right)-I_{\text{perp}}\left(t\right)\right)/I_{\text{total}}\left(t\right)$$

with *I*_total_(*t*) being the total emission signal.

It should be noted that there is disagreement about what exactly *I*_total_(*t*) in a microscope is, how it has to be calculated from *I*_para_(*t*) and *I*_perp_(*t*), and how the anisotropy decay influences fluorescence‐decay measurement. Please see [4] for an analysis of the problem.

Different from normal FLIM, intensity nonlinearity can impair the accuracy of anisotropy measurement. Intensity nonlinearity can occur with all FLIM techniques. In TCSPC FLIM, it is mainly caused by counting loss. If the count rate gets into a range where counting loss becomes apparent it is recommended to use TCSPC modules with a parallel intensity channel [134] or TCSPC modules with short dead time [4]. For applications of anisotropy‐decay imaging, please see [27, 135, 136, 137, 138, 139, 140, 141].

### 3.6 Metabolic Imaging by NAD(P)H and FAD Fluorescence

NAD(P)H (reduced form of nicotinamide adenine dinucleotide (phosphate)) and FAD (flavin adenine dinucleotide) are coenzymes involved in the cell metabolism. NADH and NADPH are spectrally indistinguishable and referred to collectively as NAD(P)H. Both NAD(P)H and FAD are fluorescent and are unique in the sense that their fluorescence intensities and fluorescence decay functions bear direct information on the metabolic state of the cells. First, the fluorescence intensities depend on the redox state: NAD(P)H is fluorescent when in the reduced state, and FAD is fluorescent when in the oxidised state. The oxidised forms of NAD(P)H and the reduced form of FAD are non‐fluorescent or weakly fluorescent. The intensity ratio of NAD(P)H and FAD—the redox ratio—is therefore an indicator of the redox state of the cell [142, 143]. Second, the fluorescence lifetimes of NAD(P)H and FAD depend on the binding to proteins [144, 145, 146]. For both molecules, the binding state changes with the state of the metabolism, and so does the fluorescence decay curve. Although usually only two decay components are considered the decay dynamics of NAD(P)H and FAD are more complex [147, 148, 149, 150]. An overview of NAD(P)H and FAD fluorescence and the corresponding literature is available in [4].

#### 3.6.1 Redox Ratio

The redox ratio is the ratio of the fluorescence intensities of NAD(P)H and FAD: RR = Intensity _NADH_/Intensity_FAD_ [142, 143, 151].

The redox ratio is a sensitive indicator of the redox state of a cell and, consequently, of its metabolic state. However, it is not a quantitative parameter. It changes with the relative concentrations of NAD(P)H and FAD in the particular cell, with the sensitivity of the instrument in the spectral emission ranges of NAD(P)H and FAD, and the laser powers and laser wavelengths used for their excitation.

#### 3.6.2 Fluorescence Lifetimes

**FIGURE 27:**
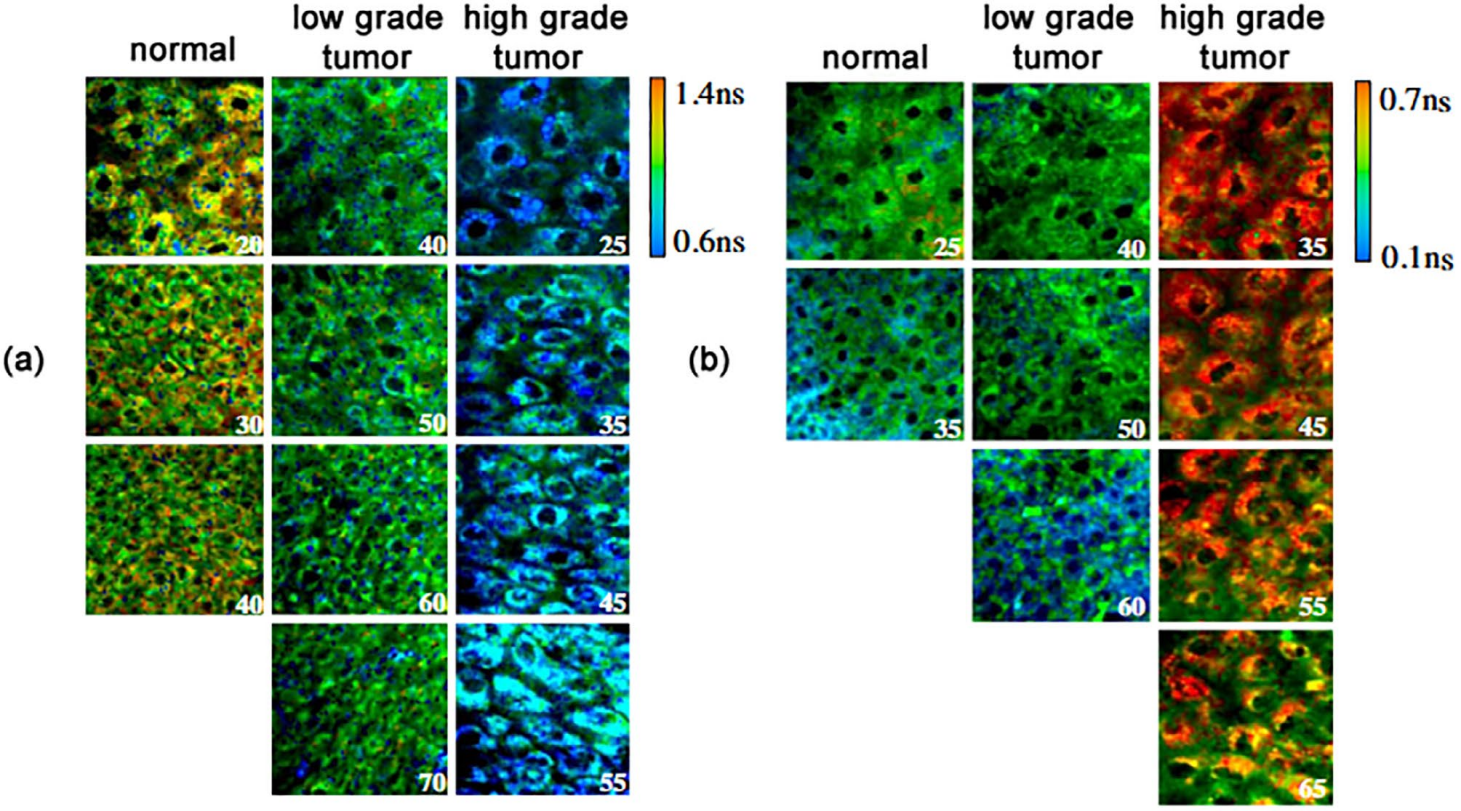
Change in the mean (amplitude‐weighted) lifetime of NADH (a) and FAD (b) with the state of the cells, from normal (oxidative phosphorylation) to high‐grade tumour (glycolysis). With permission, from [159].

What happens to the decay functions when cells pass from oxidative phosphorylation to glycolysis? Several studies have investigated this relationship [146, 147, 148, 152, 153, 154, 155, 156, 157, 158, 159, 160]. A result from [159] is shown in Figure 27. The figure shows NADH (left) and FAD (right) lifetime images for normal cells and different grades of tumours. To separate their signals, NADH and FAD were excited at different wavelengths, and the emission was collected with bandpass filters covering the respective wavelength wavelengths. It is clearly visible that the NADH lifetime becomes shorter and the FAD lifetime becomes longer as the tumour progresses. In other words, the lifetime changes with the metabolic state.

Although Figure 27 shows a clear dependence of the lifetimes on the cell metabolic state, this relationship is often ambiguous. The main reason is that NAD(P)H and FAD are not always cleanly separated. Depending on how much NAD(P)H and FAD are detected, the size or even the direction of the lifetime change varies. Moreover, an influence of the redox ratio on the apparent lifetime is unavoidable. To maximise the separation of NAD(P)H and FAD signals, different excitation wavelengths or, better, excitation‐wavelength multiplexing (Section 2.2.1.2) have to be used [4]. Another reason for inconsistent lifetimes is that the NAD(P)H and FAD decay functions depend not only on the metabolic state but also on other parameters, such as mitochondrial pH [161]. The fluorescence lifetime is therefore not a quantitative parameter to determine the metabolic state.

#### 3.6.3 Optical Metabolic Index

Skala and Walsh combined normalised values of the redox ratio, RR, and the amplitude‐weighted fluorescence lifetimes of NAD(P)H, τ_m NADH_, and FAD, τ_m FAD_, into a single ‘Optical Metabolic Imaging’, or OMI index [151].

OMI index = RR + τ_m NADH_—τ_m FAD_ (all parameters normalised to reference values).

The authors used the OMI index successfully to check the response of cultured human cancer cells from clinical biopsies to various anti‐cancer drugs [151, 157, 160, 162, 163, 164].

The OMI index is, however, also not free of problems. The redox ratio depends on the relative amounts of NAD(P)H and FAD in the cells and on instrumental details. OMI index values are therefore difficult to compare for different cell types and different instruments. That means the OMI index is a robust and sensitive metabolic indicator for a given type of cell and a given instrument, but it is not fail‐safe when different types of cells measured with different instruments are compared.

#### 3.6.4 Amplitudes of the Decay Components

In the past 10 years, improvements in time resolution and efficiency of FLIM systems and FLIM data analysis have massively increased the options for measuring the amounts of bound and unbound NAD(P)H and FAD directly. The decay curves are fitted with a double‐exponential model.

$$f\left(t\right)=a_{1}e^{-t/\tau_{1}}+a_{2}e^{-t/\tau_{2}}\cdot \left(a_{1}+a_{2}=1\right)$$

The amplitudes, *a*_*1*_ and *a*_*2*_, then represent directly the amounts of bound and unbound components. For NAD(P)H *a*_*1*_ is the unbound, for FAD *a*_*1*_ is the bound component. Both the amplitude *a*_1_ (metabolic indicator) and the ratio *a*_1_/*a*_2_ (metabolic ratio) are in use. The amplitudes turned out to be very robust, possibly even absolute, indicators of the metabolic state. They appear to be comparable even for different types of cells. The component amplitudes are therefore more and more replacing other metabolic parameters [51, 81, 83, 99, 165, 166, 167, 168, 169, 170, 171, 172].

#### 3.6.5 FLIRR Ratio

To navigate around the problems of the classic redox ratio a fluorescence–lifetime redox ratio (FLIRR) has been defined [152, 173, 174]. The FLIRR is the ratio of the fractional amplitudes of the bound decay components of NAD(P)H, *a*_2_/(*a*_1_ + *a*_2_) _NADH_ and FAD, *a*_1_/(*a*_1_ + *a*_2_)_FAD_.

$$\text{FLIRR}=\frac{a_{2}/{\left(a_{1}+a_{2}\right)}_{\text{NADH}}}{a_{1}/{\left(a_{1}+a_{2}\right)}_{\mathrm{FAD}}},\text{or},\text{with}\;a_{1}+a_{2}=1,\;\text{FLIRR}=\frac{a_{2\text{NADH}}}{a_{1\mathrm{FAD}}}$$

The authors have shown that the FLIRR is a robust parameter for the evaluation of the cell status. The advantage of the FLIRR is that it does not depend on the relative amounts of NAD(P)H and FAD in the cells, and not on the laser power, focus accuracy, focus quality or other instrument parameters. A comparative evaluation of the FLIRR, component amplitudes and component lifetimes has been given by Kalinina et al. [149].

#### 3.6.6 Combination of Metabolic FLIM With Oxygen Sensing

**FIGURE 28:**
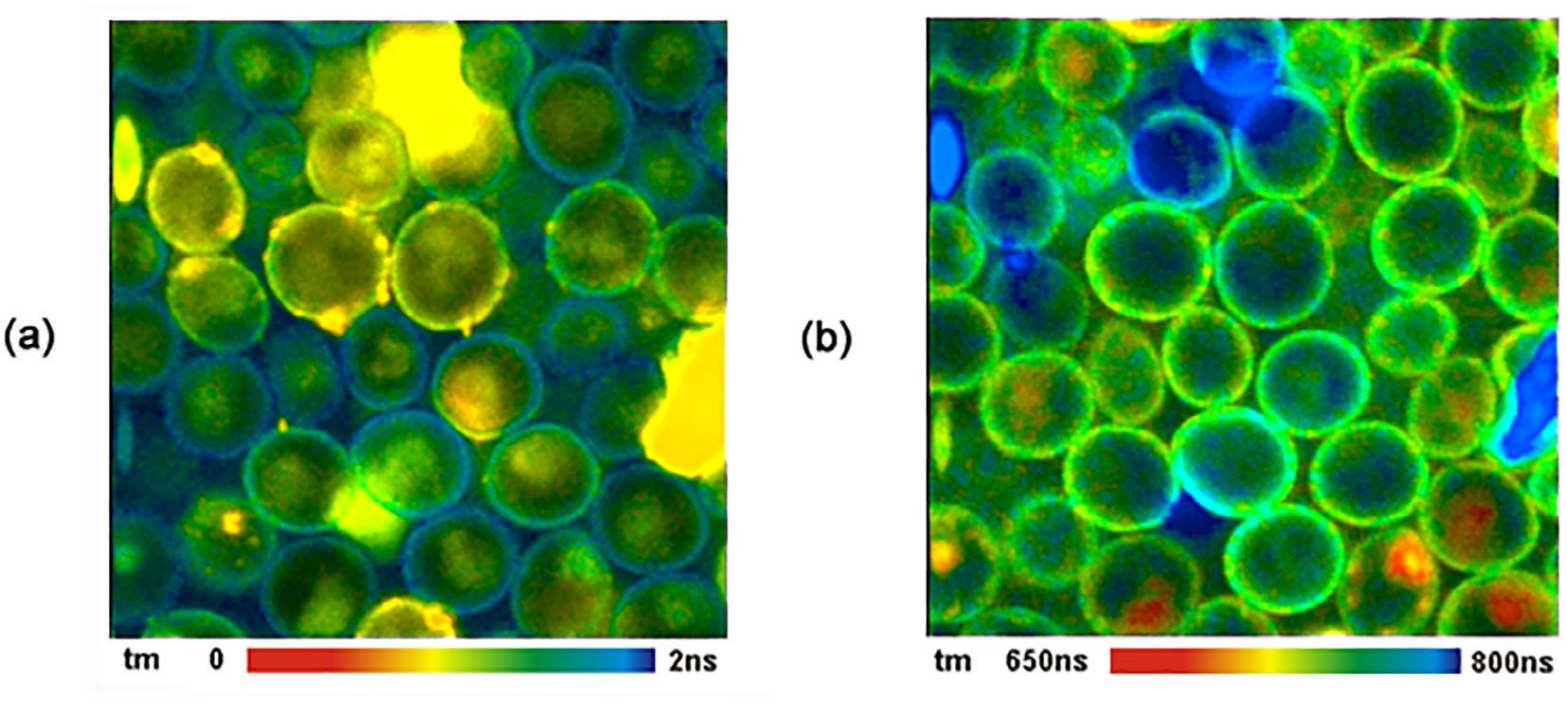
FLIM and PLIM images of yeast cells stained with (2,2′‐bipyridyl) dichlororuthenium (II) hexahydrate, recorded simultaneously. Excitation 473 nm, 256 × 256 pixels. Image size: 25 × 25 μm. Becker and Hickl DCS‐120 confocal FLIM system.

The oxygen concentration has an influence on the metabolic state of cells and tissues. It is therefore desirable to combine metabolic FLIM with oxygen sensing. This is possible by TCSPC‐based simultaneous FLIM/PLIM measurement [4, 97], see Section 2.2.1.4. There is a large number of publications on FLIM/PLIM, from the characterisation of phosphorescence probes to correlative measurement of metabolic parameters and O_2_ concentration [98, 99, 100, 175, 176, 177, 178, 179, 180]. Please see [4] for an overview. An example is shown in Figure 28.

## 4 Clinical and Preclinical Applications

Clinical applications of FLIM are almost exclusively based on metabolic FLIM, that is, on the recording of the decay functions of NAD(P)H and/or FAD with lifetimes, component amplitudes, OMI index, or FLIRR as indicators of the metabolic state. However, also other endogenous fluorophores, such as Melanin, Lipofuscin and Carotenoids can bear clinically important information on the state of the tissue. Technically, there are two classes of applications: Ex vivo samples from biopsies or material excised in surgery can be examined by FLIM, or FLIM can be performed inside the body via endoscopes or fibre optics.

### 4.1 FLIM Histology

**FIGURE 29:**
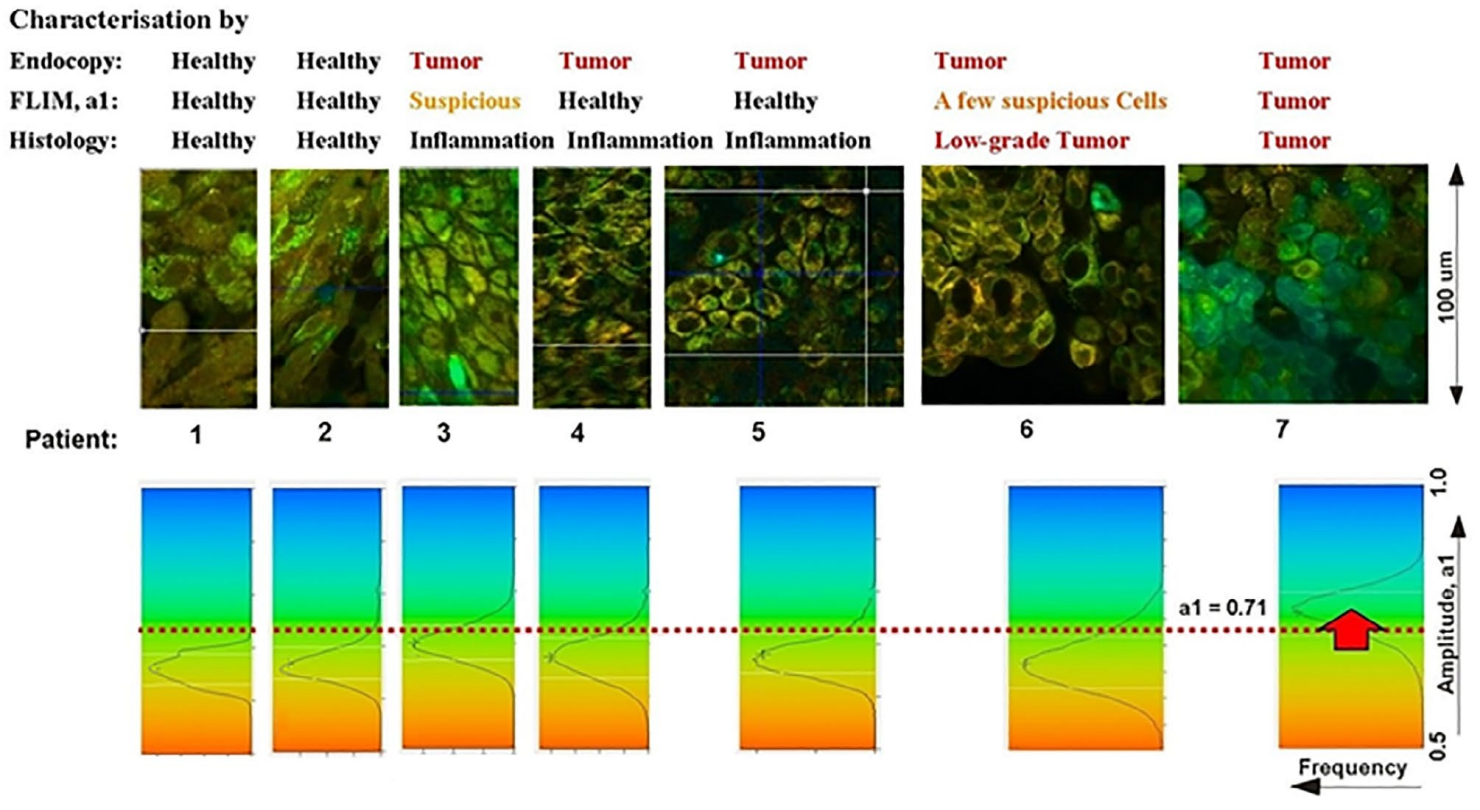
Comparison of classic endoscopy, NAD(P)H FLIM and histology. *Upper row*: FLIM images, showing the amplitude of fast decay component, *a*_1_. Red to blue corresponds to *a*_1_ = 0.5–1.0. Vertical image size 100 μm. *Lower row*: Histograms of amplitude of fast decay component, *a*_1_, showing normalised frequency of pixels with different a1 values. Patients numbered 1–7, left to right. The dotted line indicates the tumour threshold, *a*_1_ = 0.71. Histograms with a maximum above 0.71 or a long tail into the region > 0.71 indicate tumourous conditions (from [4]).

Material from surgery or a biopsy is examined by FLIM under a confocal or multiphoton microscope. NAD(P)H images are recorded, and the data are analysed by double‐exponential analysis. An example is shown in Figure 29. The amplitude of the fast decay component, *a*_1_, was used as an indicator of the metabolic state. FLIM images from seven patients are shown in the figure. Initial white‐light endoscopy had diagnosed two of them with non‐cancer lesions, and five of them with cancer [81, 83]. Figure 29 shows the results of classic endoscopy, classic histology and NAD(P)H FLIM.

The *a*_1_ FLIM images display normal *a*_1_ for patients 1 and 2, a suspiciously increased a_1_ for patient 3, an increased *a*_1_ for some of the cells from patient 6, and a massively increased a_1_ in the entire sample from patient 7. Using a selection criterion of *a*_1_ = 0.71 as a tumour threshold, the FLIM‐based diagnosis is indicated in the figure. A threshold of *a*_1_ = 0.71 may appear somewhat artificial but is supported by other NAD(P)H FLIM data [4].

Histology diagnosed patients 1 and 2 as healthy, patients 3, 4 and 5 as inflammation, patient 6 as low‐grade tumour and patient 7 as high‐grade tumour. The FLIM results were thus in good agreement with histology, and much better than the initial white‐light endoscopy results [83]. We therefore believe that FLIM can favourably complement histology. A substantial benefit of FLIM is that, in contrast to histology, the results are available within minutes after or even during the surgery.

### 4.2 Personalised Chemotherapy

**FIGURE 30:**
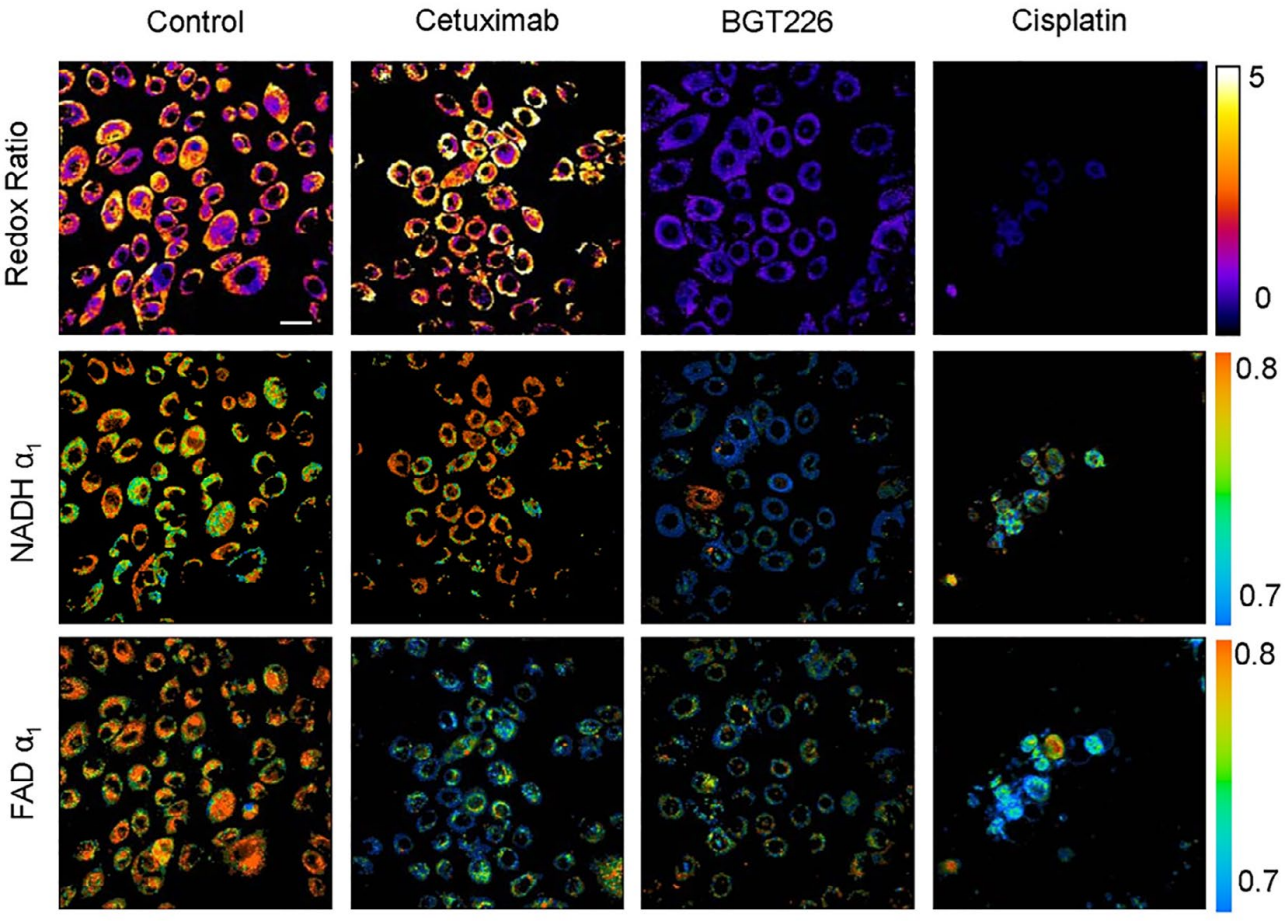
Response of SCC25 cells to Cetuximab, BGT226 and Cisplatin. Redox ratio, *a*_1_ of NADH, *a*_1_ of FAD (from [170]).

Metabolic FLIM was used to evaluate the effect of different cancer drugs on cells from biopsies of patients. There is a steep increase in the number of publications on this subject, see, for example, [33, 146, 162, 163, 164, 166, 170, 181, 182, 183, 184, 185, 186, 187, 188]. Cells from a biopsy are cultured, the cell cultures are treated with the drugs and the cells are repeatedly imaged by TCSPC FLIM. The redox ratio, the OMI index, the mean lifetime, tm, the metabolic indicator, a1 or the metabolic ratio, *a*_1_/*a*_2_ of NAD(P)H are used as an indicator of the response to the drugs. An example is shown in Figure 30. If the drug is effective a change in these parameters toward oxidative phosphorylation is observed. Within a few days, the most efficient drug can be determined and a treatment strategy for the patient be developed. If the technique can be transferred into routine clinical use it has the potential to revolutionise cancer treatment.

### 4.3 T Cells and Macrophages

T cells and macrophages play an important role in immune response and in tumour suppression. Assessment of the metabolic function of these cells is therefore important to the diagnosis of diseases and monitoring of therapeutic efficacy. Walsh et al. performed autofluorescence FLIM of T cells [189]. They were able to classify the cells based on the τ_m_ of NAD(P)H, the τ_m_ of FAD and the redox ratio. Heaster et al. [190, 191] used τ_m_ of NAD(P)H and FAD in combination with the redox ratio to assess macrophage heterogeneity on the cell level. Izosimova et al. investigated T cells in normal and tumour‐bearing mice by FLIM of NAD(P)H [165]. In T cells of the tumour mice, they found an increase in *a*_1_, that is, an increase in free NAD(P)H. Moreover, triple‐exponential analysis showed that a decay component from NADPH appeared in tumour‐bearing mice which was not detectable in normal mice.

### 4.4 Metabolic FLIM of Oocytes

Sanchez et al. have shown that 2‐photon excited TCSPC FLIM of NAD(P)H and FAD can be used to differentiate normal oocytes from those with metabolic dysfunctions [168]. The authors used the fluorescence intensities, the component lifetimes, τ_1_ and τ_2_, and the amplitudes of the bound components to quantify the metabolic state of mouse embryos. The authors also conducted studies of the impact of the illumination on the blastocyte development rates. They did not find measurable differences between embryos that had been exposed to the FLIM measurement and reference embryos which had not been illuminated. More publications related to metabolic FLIM of oocytes have appeared in the following years: Metabolic FLIM combined with SHG imaging of the spindle [169] proved useful for embryo assessment, and differences in the metabolic parameters of euploid and aneuploid blastocytes [171], variations in the metabolic parameters between different blastocytes and within the same blastocytes over time [172], and changes with patient age and between mature and immature oocytes were found [192]. An overview of non‐invasive metabolic profiling of cumulus cells, oocytes, and embryos by TCSPC FLIM has been given in [193].

### 4.5 Stem Cells

**FIGURE 31:**
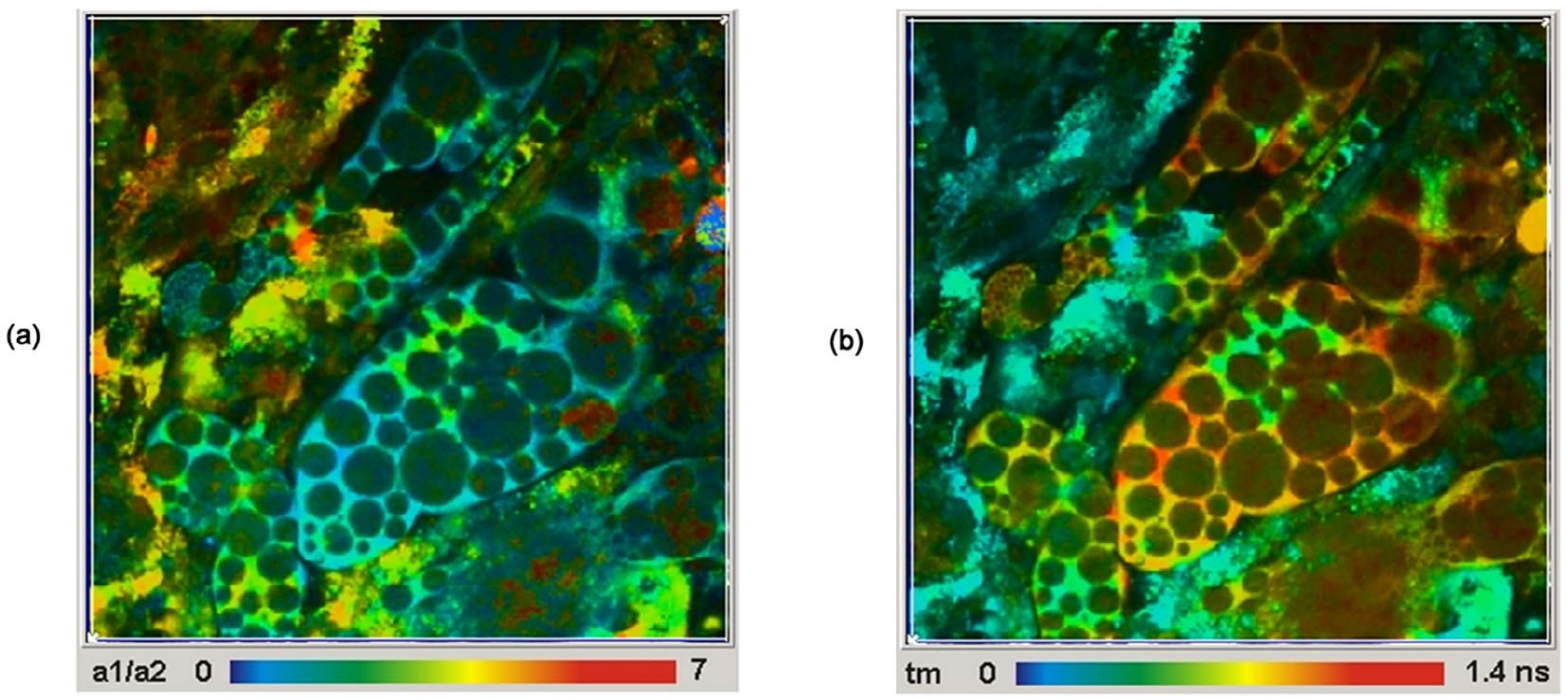
FLIM of human salivary gland stem cells. Differentiated cells have significantly lower *a*_1_/*a*_2_ (a) ratio and longer mean lifetime (b). Data courtesy of Aisada Uchugonova and Karsten König, Saarland University, Saarbrücken.

Gou et al. used FLIM of the decay components of NAD(P)H to distinguish human mesenchymal stem cells from differentiated progenies [194]. Uchugonowa and König applied two‐photon autofluorescence lifetime imaging to human and animal stem cells [195, 196]. Using both the amplitude ratio, *a*_1_/*a*_2_, and the amplitude‐weighted lifetime, tm, they found significant changes in the fluorescence lifetimes on differentiation and maturation of the cells. An example is shown in Figure 31. The differentiated cells have smaller *a*_1_/*a*_2_ ratios and longer mean lifetime, *t*_m_.

Meleshina et al. simultaneously tracked the redox ratio and the *a*_1_/*a*_2_ (unbound/bound) ratio during the maturation of stem cells. For different stages of differentiation, they observed metabolic shifts both in the direction to glycolysis and to oxidative phosphorylation [197, 198].

Qian et al. used metabolic FLIM to track and predict the differentiation of stem cells into cardiomyocytes for regenerative medicine [199]. Molugo et al. tracked the reprogramming status of patient‐derived elytroid progenitor cells to select high‐quality donor‐specific induced pluripotent stem cells [200].

### 4.6 Metabolic FLIM of Macroscopic Objects

**FIGURE 32:**
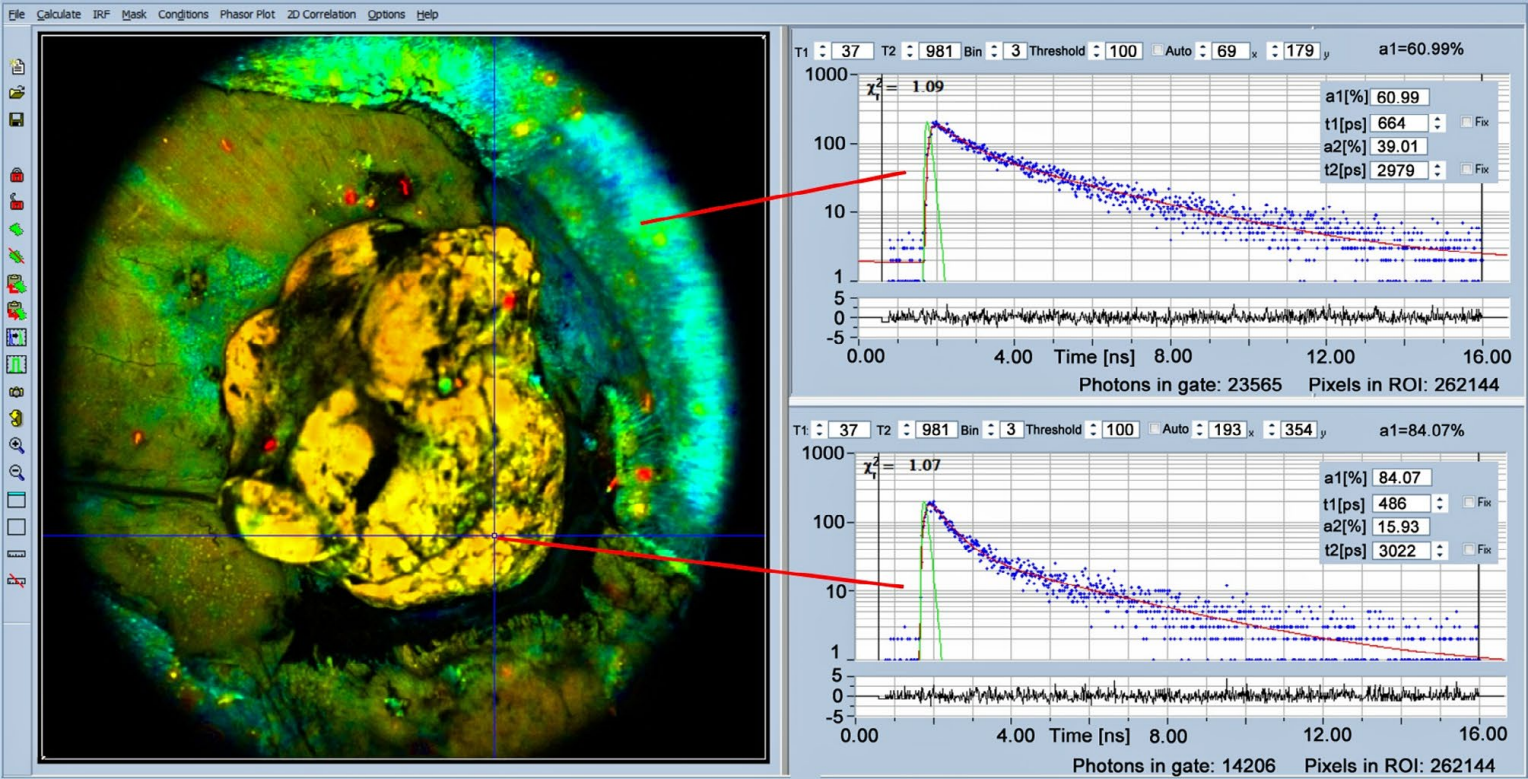
Open tumour in a mouse. Colour represents the amplitude, *a*_1_, of the fast decay component. The borders of the tumour are clearly defined. Decay functions in non‐tumour and tumour regions are displayed on the right. Decay parameters, residuals and χ^2^ shown in the curve windows. Becker & Hickl DCS MACRO system, image size is 15 mm. Adapted from [104].

TCSPC FLIM can record metabolic‐FLIM images of areas as large as 18 mm in diameter. The data have high spatial resolution, and high resolution of the fluorescence decay functions [4, 77, 104]. The technique delivers colour‐coded images of the amplitudes of the decay components. These show the metabolic state of the tissue quantitatively. As an example, an open tumour in a mouse is shown in Figure 32. The colour represents the amplitude, a1, of the fast decay component of NAD(P)H. Image size is about 18 × 18 mm. As can be seen from the image, the tumour is clearly identified in the *a*_1_ data: In the tumour *a*_1_ is 0.84, outside the tumour it is 0.61. Details of the imaging system and further results are described in [4, 104, 201].

Despite the potential to deliver quantitative results, the technique has not been used for intraoperative in vivo imaging yet. One reason is the small working distance. To obtain high collection efficiency the scan lens must be close to the image plane, which blocks access to the surgery area. The problem can be mitigated by putting a periscope in front of the scanner, as has been shown in [4].

### 4.7 Ex Vivo Biopsy by Macro FLIM

**FIGURE 33:**
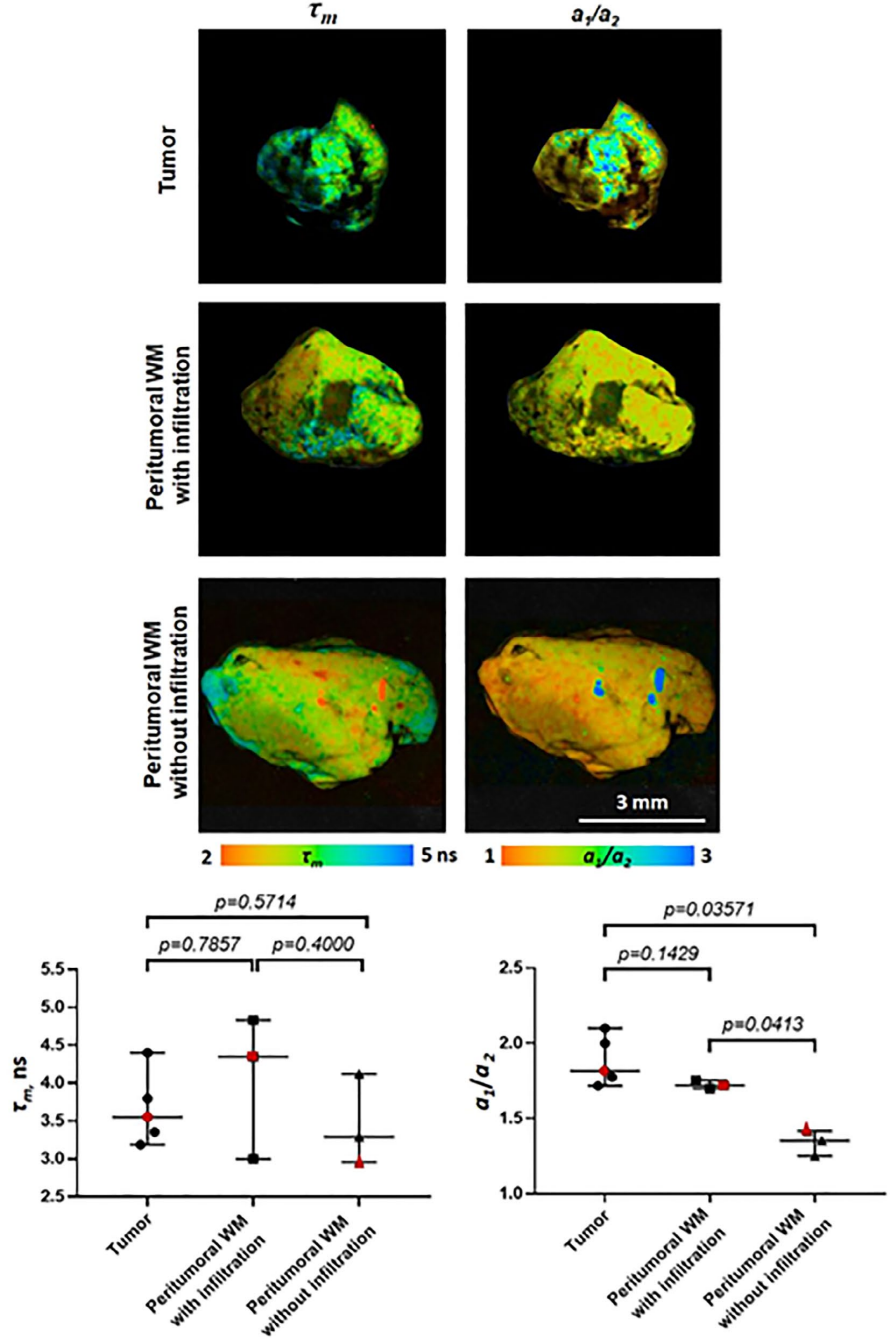
Macro‐FLIM of human glioblastoma and the peritumoural white matter with and without infiltration by tumour cells. *Lower figures*: quantification of tm and *a*_1_/*a*_2_ ratio in tumours and the peritumoural white matter. Scatter dot plot displays the measurements for individual samples (dots) and the median, minimum and maximum (horizontal lines). tm is the mean fluorescence lifetime. *a*_1_/*a*_2_ is the ratio of relative contributions of short and long components. Adapted from [107].

Macroscopic FLIM can be used for optical biopsy ex vivo [107, 188]. Clinical results using a macro‐FLIM system have been demonstrated in neuro‐oncology, where glioblastoma was differentiated from the white matter in the NAD(P)H spectral channel by the higher free/bound ratio (*a*_1_/*a*_2_). An example is shown in Figure 33.

### 4.8 Multiphoton Lifetime Tomography of Human Skin

**FIGURE 34:**
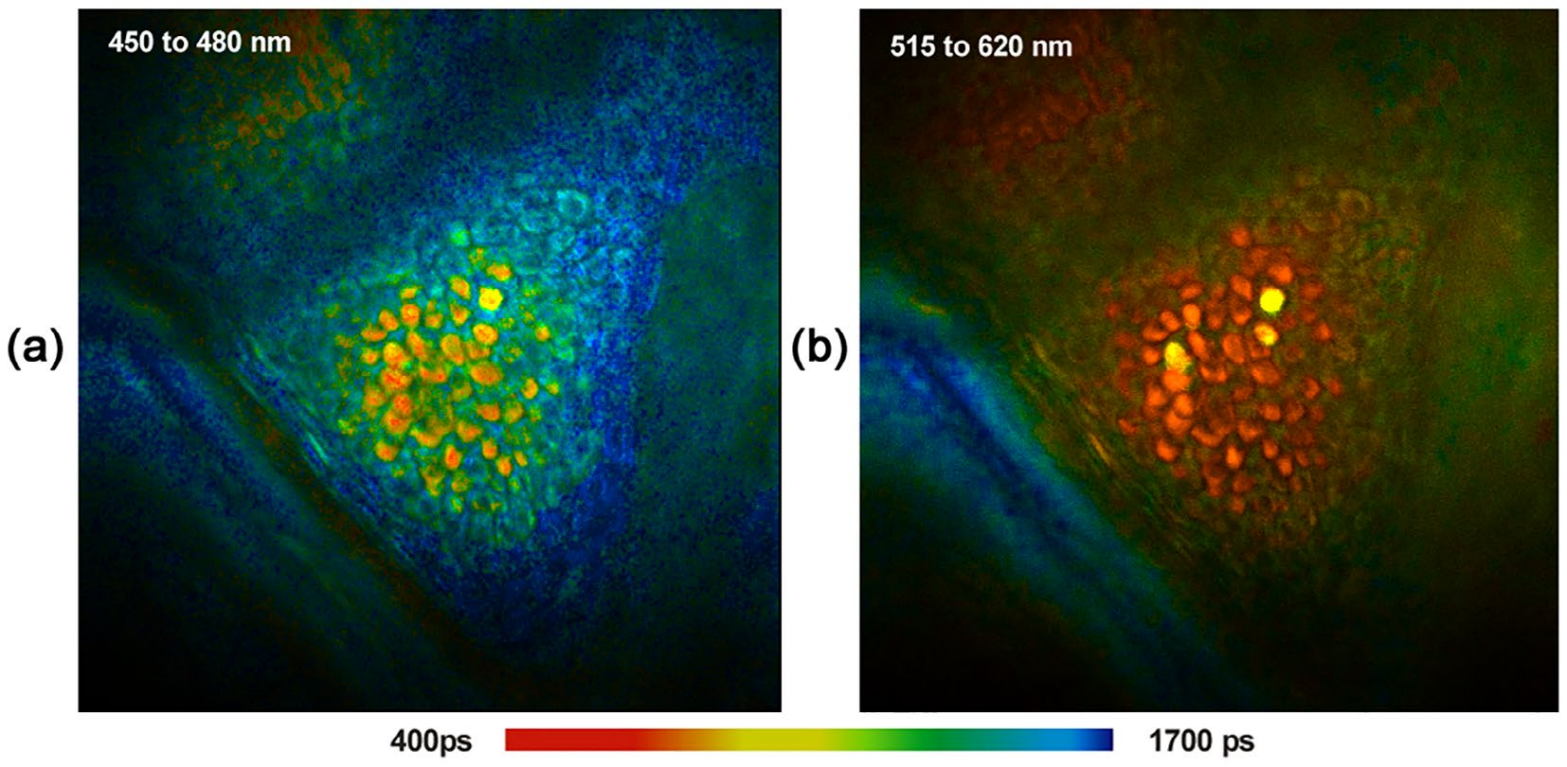
FLIM of the stratum granulosum at the forearm of a human volunteer. Data courtesy of Washington Sanchez and Michael Roberts, University of Queensland, Brisbane, Australia. (a) 450–480 nm emission band, containing mainly emission from NAD(P)H. (b) 515–620 nm, containing the emission of flavins and melanin. Image size: 300 × 300 μm, resolution 512 × 512 pixels.

Multiphoton tomography of human skin exploits the fact that two‐photon excitation in combination with non‐descanned detection delivers optically sectioned images of tissue layers as deep as 100 μm. The three‐dimensional tissue structure can be reconstructed from the data at sub‐cellular resolution. The technique goes back to the work of Gratton, König, Masters, So and Tromberg who showed that in vivo two‐photon imaging of cells and, especially, human skin is possible without impairing the viability. Since its introduction 20 years ago [1, 202, 203], FLIM of human skin has been developed to the stage of clinical application [196, 204, 205, 206, 207, 208, 209, 210]. Please see [4] for more references. An example is shown in Figure 34.

**FIGURE 35:**
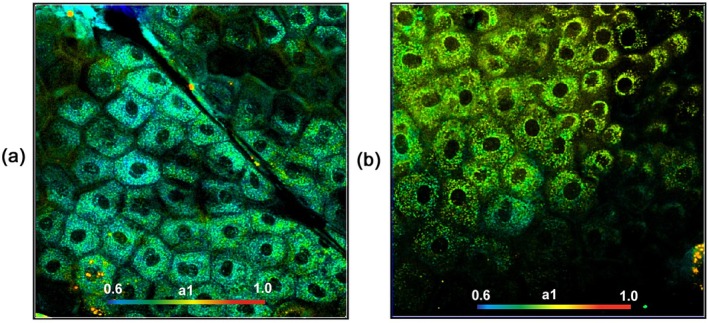
Mouse skin tissue, NAD(P)H short lifetime amplitude (*a*_1_) images. (a) Healthy skin tissue. (b) Tumour tissue. Image size: 200 × 200 μm, resolution 512 × 512 pixels. FLIM data from [211], images reprocessed by SPCIMage NG, MLE fit [110].

Also in skin data, healthy tissue and tumour tissue can be distinguished by the amplitudes of the NAD(P)H‐fluorescence components [211]. An example is shown in Figure 35. The images show two‐photon excited NAD(P)H images of the stratum spinosum of mouse skin. Healthy tissue is shown on the left, and tumour tissue on the right. As expected, the amplitude of the free‐NAD(P)H component, *a*_1_, is significantly higher in the tumour.

### 4.9 Malignant Melanoma

**FIGURE 36:**
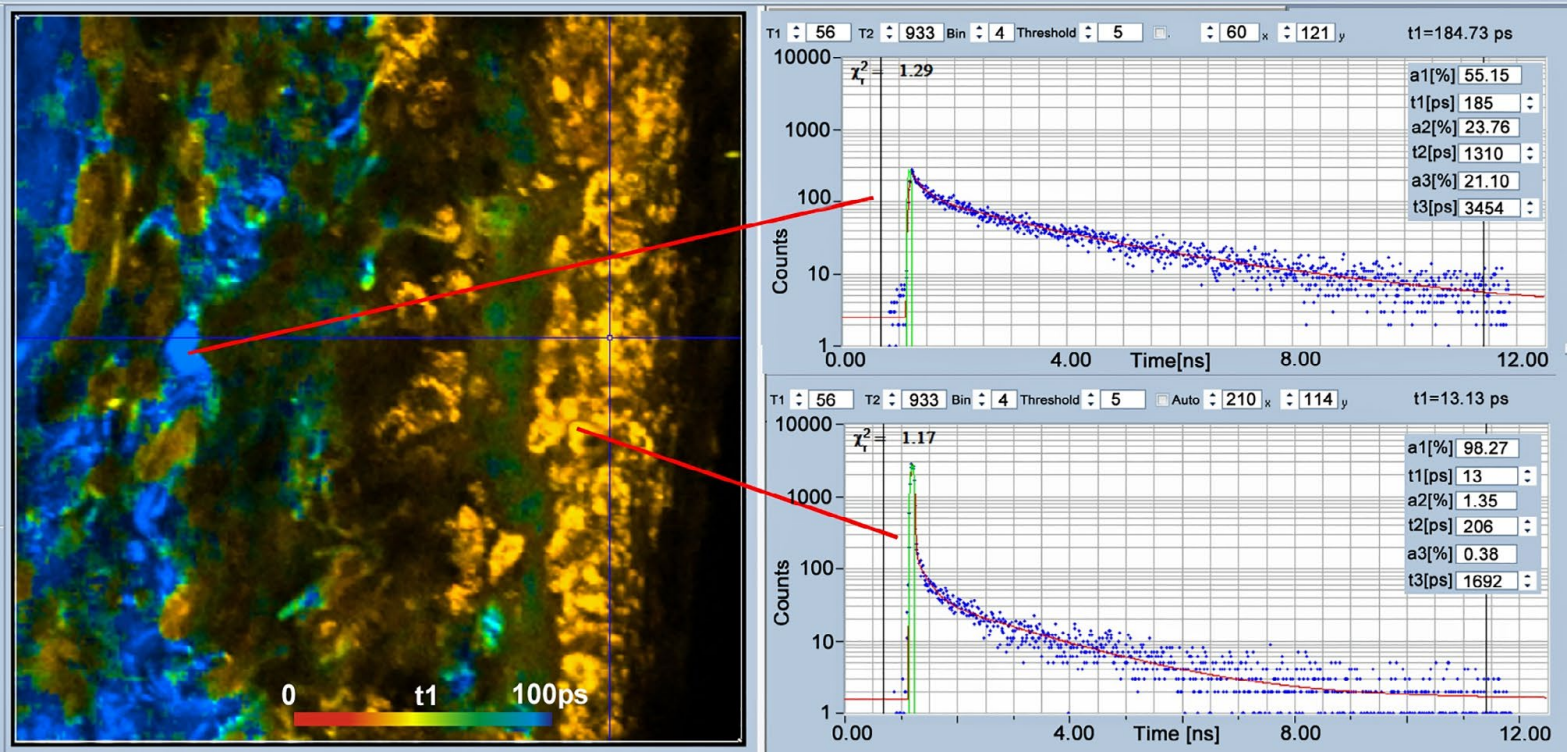
Axial cross‐section through melanoma tissue. Colour‐coded image of the lifetime of the fast component, *t*_1_, of a triple‐exponential fit of the data. The fast decay component in the tumour has a lifetime, *t*_1_, of 13 ps and amplitude, *a*_1_, of 0.98. Red to blue corresponds to 0–100 ps. Decay curves in characteristic spots of the image are shown on the right. Image size: 220 × 220 μm, resolution 512 × 512 pixels.

There have been earlier indications that the fluorescence lifetimes in malignant melanoma are shorter than in healthy tissue and in benign skin lesions [212, 213, 214]. With the limited time resolution of the instruments available at this time, it was not possible to determine what the true lifetime was, whether the decay functions were single‐ or multiexponential, and which of the decay components were the source of the short apparent lifetime. Ultra‐fast TCSPC FLIM (IRF width 18 ps, FWHM, [111]) has shown that the lifetime is indeed extremely short. Figure 36 shows a lifetime image of an axial section through melanoma tissue. Superficial layers are shown on the right in the image, and deeper layers on the left. Colour coding shows the lifetime of the fast decay component, t1, obtained by fitting the decay data by a triple‐exponential model. Decay curves from selected spots of the image are shown on the right. The sharp peak in the decay curve from the superficial layer (bottom, right) shows visibly that there must be an extremely fast decay component. The fit delivers a lifetime, *t*_1_, of 13 ps and an amplitude, *a*_1_, of 98% for this component (Remark: The peak is not SHG, this was excluded by filtering). The peak is not present in the decay curve from deep tissue layers, see top right. The component lifetimes in these areas are in a more or less ‘normal’ range, and compatible with a mixture of NAD(P)H and FAD, and possibly FMN [215]. Lifetime images taken from samples of benign pigmented lesions did not show the ultra‐fast component. Please see [15] for details.

### 4.10 Ophthalmic FLIM

**FIGURE 37:**
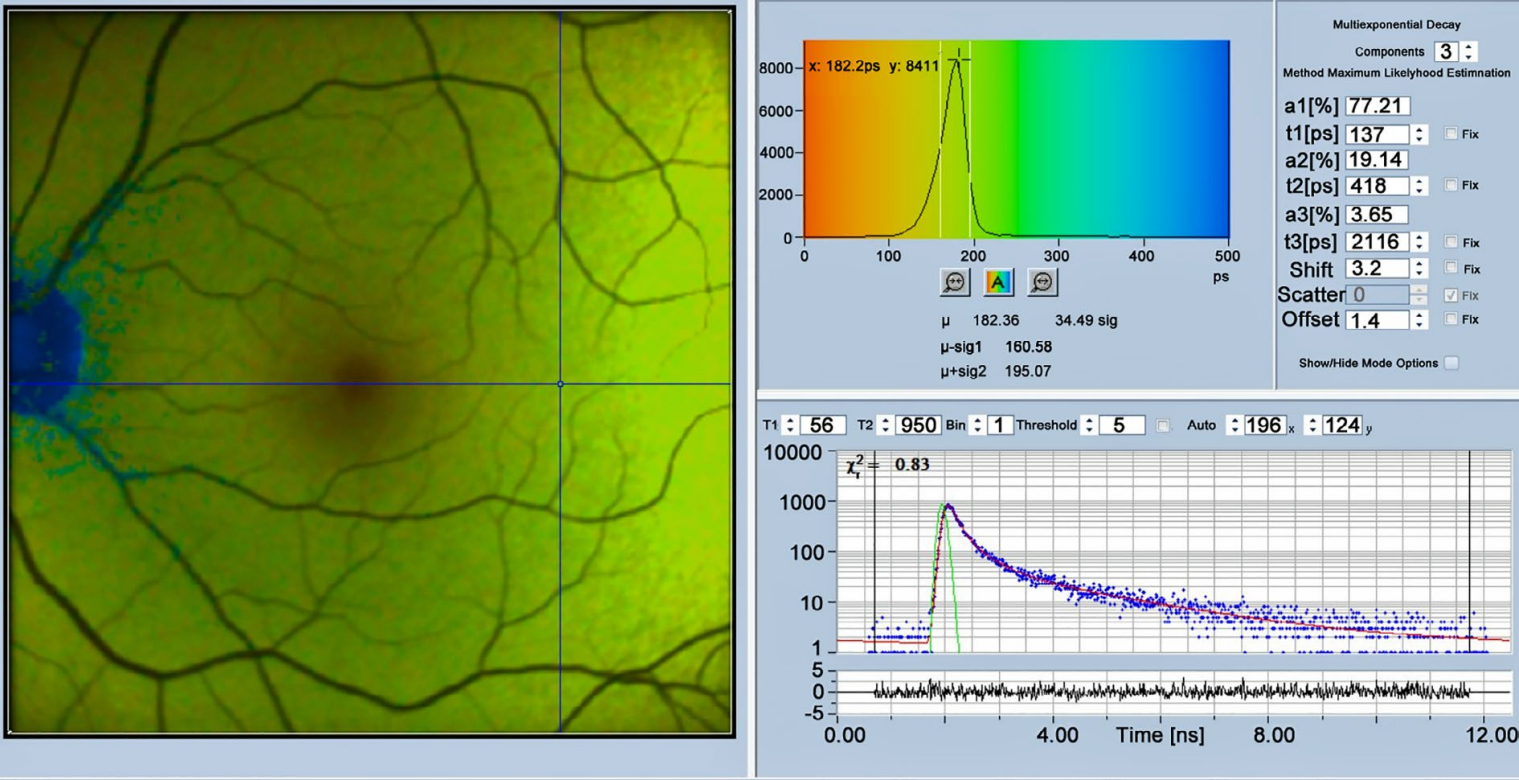
FLIO image. Triple‐exponential decay analysis, image of the mean lifetime, tm12, of the fast and the medium decay component. Distribution of tm12, colour coding of tm12, and parameter values are shown upper right. Decay curve at the cursor position is shown lower right. Residuals and χ^2^ shown in the decay‐curve window.

The introduction of FLIM into ophthalmic imaging was pioneered by Schweitzer and Hammer [216, 217]. Their work eventually resulted in the development of a clinical ophthalmic FLIM system by Heidelberg engineering. Ophthalmic FLIM (FLIO) uses the typical ophthalmic‐scanner principle. The ocular fundus is scanned through the pupil of the eye by a ps‐diode laser beam, the fluorescence is collected back through the pupil, descanned, filtered, detected by two fast hybrid detectors, and recorded by TCSPC FLIM [4, 16, 218]. Data recording compensates for eye motion. Data analysis takes into account the early arrival of fluorescence from the lens of the eye, delivering clean images even from cataract patients [4]. A typical FLIO FLIM image obtained with the Heidelberg Engineering instrument is shown in Figure 37. The data were analysed by MLE fitting with a triple‐exponential model. The colour shows the mean lifetime, *t*_12_, of the fast and the medium decay component. These components come from the fundus. The slow component, *t*_3_, is fluorescence from the lens of the eye, and is not included in *t*_12_ [4, 110].

Schweitzer et al. have shown that the decay components can be associated with different fluorophores and different tissue layers [219, 220]. A review on FLIM ophthalmoscopy can be found in [35]. General clinical studies with the Heidelberg FLIO instrument are described in [221, 222, 223]. There are numerous studies on metabolic alterations due to diabetes mellitus, retinitis pigmentosa, Stargardt disease, arterial occlusion, retinal pigment atrophy, central serous chorioretinopathy, mydriasis, glaucoma, macular telangiectasia, AMD, macular holes, pigment epithelial detachments and diabetic retinopathy. Please see [4], chapter ‘Ophthalmic FLIM (FLIO)’.

An interesting application of ophthalmic FLIM is the possible detection of Alzheimer's disease. Alzheimer's disease is related to the expression of the phosphor tau 181 protein. This protein is present in the eye and can be detected via its fluorescence. In the FLIO data, it shows up as an increased intensity contribution of the medium lifetime component [218, 224].

### 4.11 FLIO With Two‐Photon Excitation

The lens of the eye is intransparent for wavelengths shorter than 400 nm. This precludes the use of the metabolically and spectroscopically most relevant fluorophore, NAD(P)H. It has been shown in pre‐clinical applications that the problem can be solved by two‐photon excitation in combination with adaptive optics [225]. The spatial resolution of the instrument is so good that individual rods and cones are resolved [225, 226].

### 4.12 FLIM Endoscopy

Recording metabolic FLIM through an endoscope would, of course, be an ideal solution to clinical imaging. However, as mentioned in Section 1, there are several technical obstacles: It is optically impossible to obtain simultaneously a large field of view, high collection efficiency, and small diameter of the endoscope. Moreover, the glass of the endoscope fluoresces under UV excitation, which contaminates to signal from the sample. The problem can be mitigated by wide‐field imaging by a gated or modulated image amplifier, a gated SPAD array or a TDC‐based SPAD array and wide filed illumination through a separate optical fibre. The excitation then does not directly generate glass fluorescence in the endoscope. However, some glass fluorescence may be excited by excitation light scattered back from the sample into the endoscope. Moreover, a FLIM system of this type has no depth resolution and thus records mainly fluorescence from the surface of the tissue.

**FIGURE 38:**
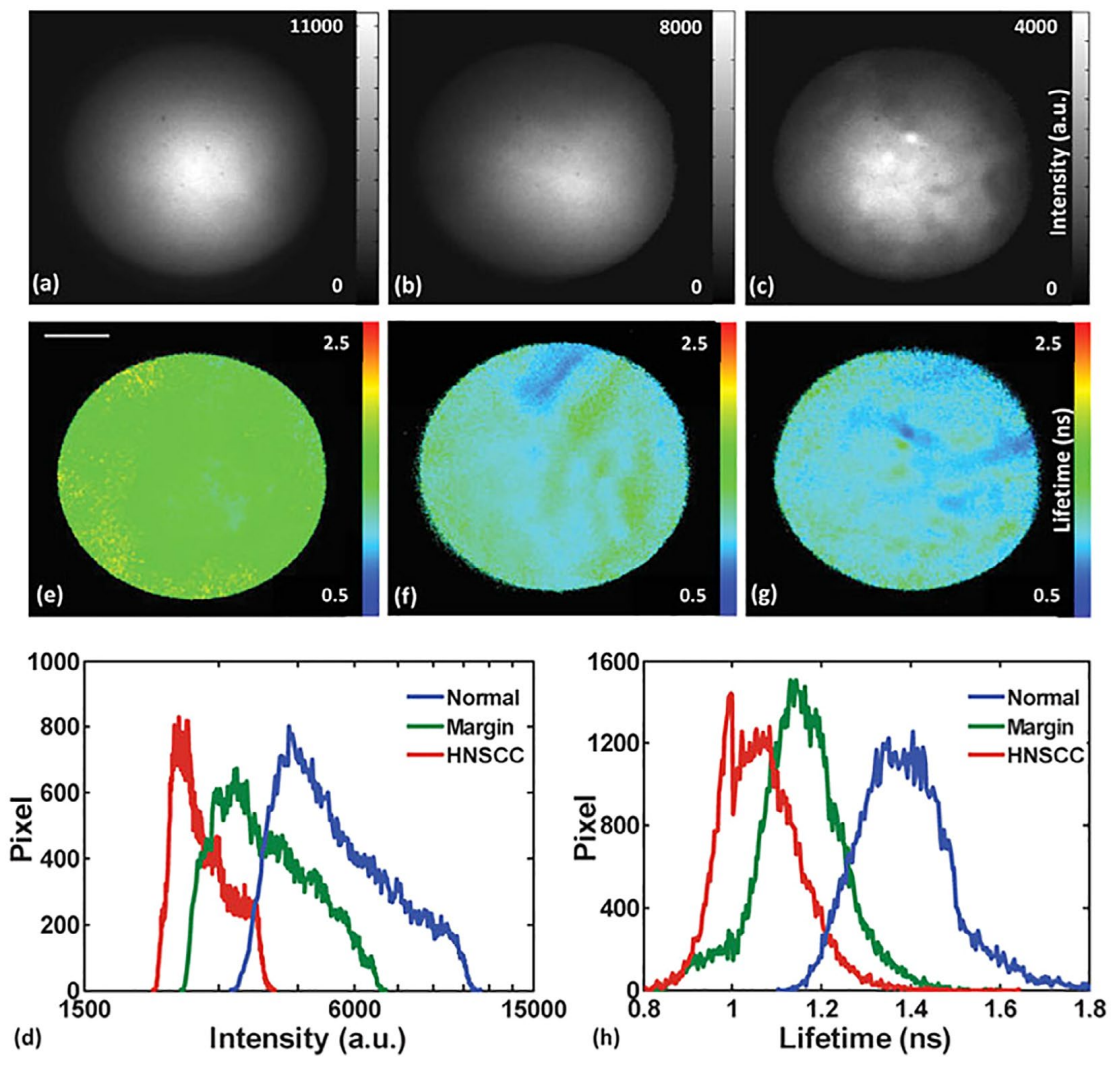
Autofluorescence fluorescence lifetime images of human buccal mucosa: (a)–(c) depict the intensity images, and (e)–(g) depict the average lifetime images from three areas: normal, tumour and adjacent normal‐tumour, their corresponding histograms are depicted in (d) for intensity and (h) for an average lifetime. HNSCC, head and neck squamous cell carcinoma (from [23]).

Sun et al. [23] developed a clinically compatible endoscopic FLIM system based on excitation by a 337 nm Nitrogen laser, a fibre‐bundle endoscope, and wide‐field imaging by a gated image intensifier. To reduce glass fluorescence in the fibre bundle they transferred the excitation light to the image area by a separate optical fibre. With a minimum gate width of 0.2 ns and a laser pulse width of 0.7 ns, the effective IRF width was ~800 ps or longer. This allowed for the detection of the apparent lifetime of NAD(P)H but not of the amplitudes of the decay function. A result is shown in Figure 38.

### 4.13 FLIM‐Assisted Surgery

**FIGURE 39:**
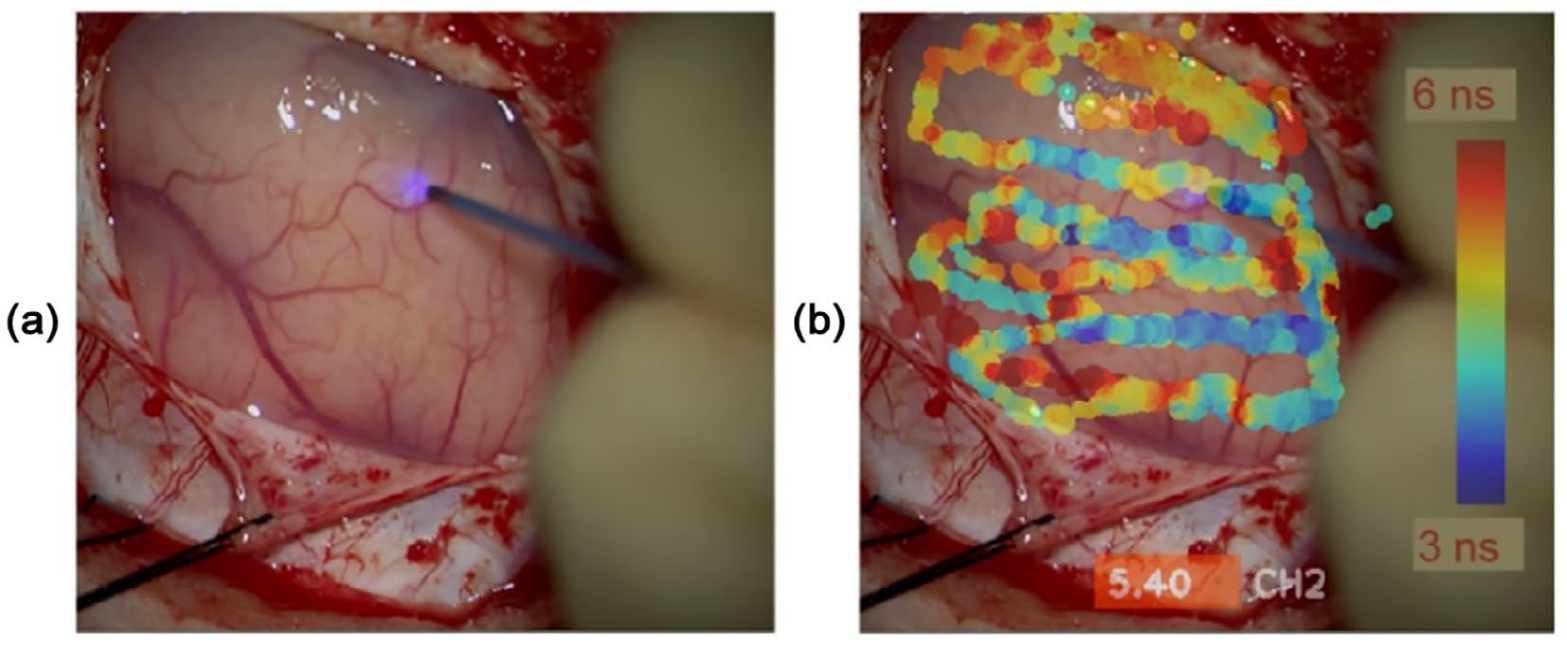
*Left*: White light image of surgery area, with fibre‐optic probe. *Right*: White‐light image with lifetime overlay.

Another solution uses a combination of white‐light imaging by a CMOS video camera and single‐point fluorescence‐decay measurement through an optical fibre. Excitation is performed by a 355 nm Q‐switched laser with 2 kHz repetition rate. The fluorescence is detected by an MCP PMT in the analogue mode and recorded by a fast digitizer. Separate fibres are used for excitation and detection to avoid contamination by glass fluorescence. The end of the fibre probe is identified in the camera image, and the corresponding pixels in the CMOS image are colour‐coded by the measured lifetime [63, 65, 106, 227, 228, 229]. By moving the fibre over the image area, the area of interest is sampled point by point, and the corresponding pixels are colour‐coded in the CMOS image by the fluorescence lifetime. The tumour to non‐tumour transitions can be found and used to guide the surgery. An example is shown in Figure 39. The CMOS white‐light video image is shown on the left, and the CMOS image with the fluorescence‐lifetime overlay on the right.

A similar fibre‐based system with TCSPC detection has been described in [85]. The system uses a 375‐nm diode laser for excitation and a 785‐nm guidance laser for localising the excited spot in the camera image. The photons are detected by a fast hybrid detector and recorded in a TCSPC module. From the information available in [85] the time‐resolution (IRF width) can be estimated to be about 120 ps, full width at half maximum. With a time resolution this short, the system is able to determine not only the apparent lifetime of NAD(P)H but also the metabolic indicator, *a*_1_, or the metabolic ratio, *a*_1_/*a*_2_. Data analysis was performed by phasor analysis. With an estimated count rate of 10^6^ s^−1^, which is safely within the counting capability of the TCSPC module, 10.000 photons can be recorded in 10 ms, giving a theoretical lifetime accuracy of 1%. The system can be used under bright illumination of the surgery area by LEDs. This is achieved by multiplexing the TCSPC measurement in anti‐phase with the illumination (Section 2.2.1.2). The system has been shown to work for optical biopsy of gastrointestinal tumours [230]. A similar system with two multiplexed lasers of 378 nm and 445 nm and three spectral TCSPC channels has been described by Herrando et al. [82].

### 4.14 Optical Biopsy

The term ‘Optical Biopsy’, relates to optical detection of tissue parameters through endoscopes or fibre optics, and the assessment of the tissue conditions in situ. The first experiments that could be termed as optical biopsy have been done by Alfano et al. who tried to record spectral profiles from cancerous and normal rat kidney and prostate tissues [231]. An early fluorescence‐lifetime biopsy system has been described by Butte et al. [62]. The system used a nitrogen laser for excitation, a fibre probe with separate excitation and detection fibres, an MCP PMT in the analogue‐detection mode, and a digital oscilloscope for recording decay profiles. Despite the limited time resolution, the system was able to distinguish brain tumours from normal brain tissue in a human clinical trial. Nie et al. [64] described a similar system with a 355 nm Nd:YAG laser of 300 ps pulse width and a acousto‐optical filter for emission wavelength selection. A fibre‐endoscopic system for simultaneous fluorescence‐lifetime and Raman detection has been described by Lagarto et al. [232]. The fluorescence‐detection part uses a high‐frequency pulsed diode laser of 375 nm and dual‐channel TCSPC detection. The TCSPC detection was multiplexed with field illumination.

**FIGURE 40:**
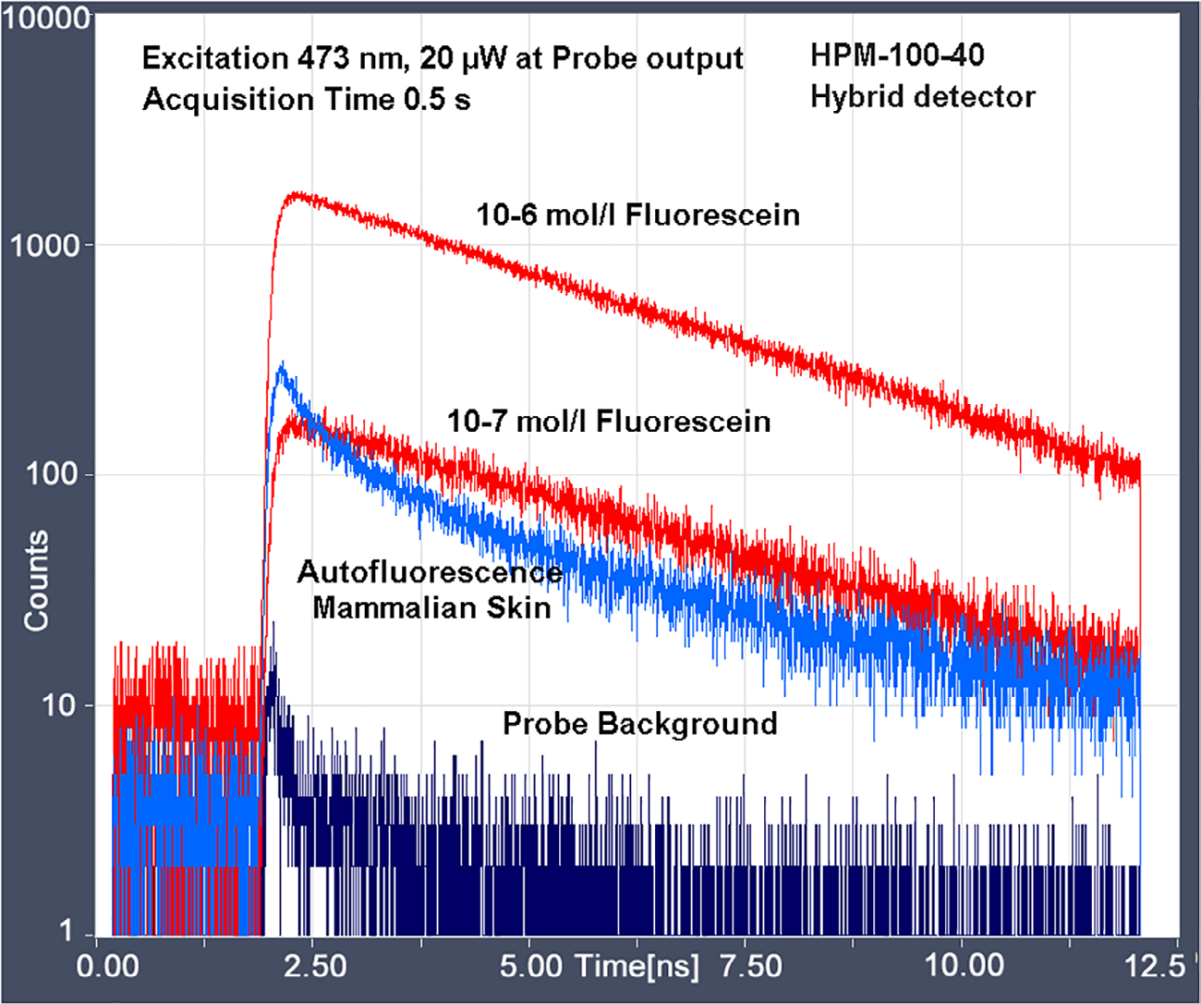
Decay curves measured through an optical fibre endoscope. Fluorescein solution, 10^−6^ and 10^−7^ mol/L and decay curve of mammalian skin compared to the background signal of the fibre probe itself. Collection time 0.5 s, laser power at probe output 20 μW (from [4]).

A commercially available high‐resolution optical‐biopsy system based on TCSPC is described in [4, 233]. A 375‐nm picosecond‐diode laser is used for excitation. A fibre probe with a replaceable tip and separate excitation and detection fibres delivers the excitation light to the tissue and feeds the emission light back to a fast hybrid detector. The decay curves are recorded by TCSPC. The IRF width is about 120 ps, full width at half maximum. 20 μW of laser power at the fibre output are enough to record decay curves within 0.5 s (Figure 40). The system can also be used with a multi‐wavelength detector and with fast time‐series recording. A high‐resolution optical‐biopsy system based on this system has been used by Lukina et al. [234]. A similar system with a longer optical‐biopsy needle and two parallel spectral TCSPC channels has been used by Zherebtsov et al. [235] to characterise inoculated hepatocellular carcinoma (HCC) and adjacent liver tissue by NAD(P)H and FAD decay parameters [236, 237, 238].

## 5 Summary

The fluorescence decay function of a fluorophore is a direct indicator of its interaction with its molecular environment. By placing characteristic fluorophores in defined molecular locations or by using endogenous fluorophores in known binding conditions and detecting their fluorescence decay, molecular information on biological systems can be obtained. Therefore, FLIM is much more than a simple contrast technique in microscopy—it is a technique of molecular imaging.

FLIM techniques can be classified into time‐domain and frequency‐domain techniques, analogue and photon counting techniques and scanning and wide‐field techniques. Virtually all combinations of these techniques are in use. The resulting instruments differ in time resolution, photon efficiency, depth discrimination, optical sectioning capability, suppression of scattering, acquisition speed and intensity range they can be applied to. Differences exist also in the range of applicable excitation pulse rate, and in the principles of the optical systems, the techniques can be used with.

These differences can be substantial. For instance, a technique that restricts FLIM to the detection of simple fluorescence lifetimes does not exploit the full information contained in the fluorescence decay function. This is important when the technique is used for protein interaction experiments by FRET, where interacting and non‐interacting donor fractions must be distinguished. It is also essential in label‐free (autofluorescence imaging), where several fluorophore or fluorophore fractions in different binding states to proteins are present. These fluorophore fractions display different fluorescence lifetimes which can only be distinguished by recording the full decay function at high time resolution and analysing the data by multi‐exponential decay analysis.

The need for multi‐exponential decay analysis has an influence also on the technique used for spatial resolution. Multi‐exponential decay analysis can only be successful if out‐of‐focus fluorescence, lateral‐scattering and background fluorescence from the optics are suppressed. Scanning techniques suppress such unwanted signals, but wide‐field techniques do not. This splits FLIM techniques into two general groups: Wide‐field techniques with gated cameras or gated SPAD sensors that are fast but do not efficiently resolve complex decay functions, and scanning techniques with TCSPC detection which resolve complex decay profiles but are limited in recording speed by the frame rate of the scanner.

There is a common misconception about the acquisition speed a FLIM technique reaches. The acquisition speed in the first instance depends on the photon rate that is available from the sample, not on the photon rate a recording technique can process. Of course, a wide‐field technique is intrinsically faster because it records decay data in many pixels in parallel. However, these photons do not necessarily come from the desired image plane and from the right lateral position. The signals may also be contaminated by glass fluorescence, especially if excitation in the UV is used. Moreover, subsequent measurements with different gate delays are necessary to obtain the fluorescence lifetime or, more challenging, at least some approximate multi‐exponential decay information. As a result, the wide‐field technique needs more photons than a scanning technique and loses a large part of its speed advantage.

An often neglected feature of a FLIM technique is its ability to record several parameters of a biological system simultaneously and in their mutual dependence. In this respect, scanning TCSPC FLIM with its multi‐dimensional recording principle is at advantage. Techniques like multi‐wavelength FLIM, excitation‐wavelength multiplexed FLIM, simultaneous FLIM/PLIM or fast triggered accumulation of dynamic lifetime effects are logical extensions of the TCSPC FLIM principle.

Applications of FLIM include measurements of the local molecular conditions in biological systems, such as pH, ion concentrations, glucose concentration, local viscosity and a lot of other physiologically relevant parameters, membrane‐potential measurements, FRET measurements and FLIM of endogenous fluorophores like NAD(P)H and FAD. NAD(P)H and FAD FLIM have gained enormous potential in that the fluorescence‐decay functions of these compounds yield direct access to the metabolic state of cells and tissues. Due to their relationship to metabolism, these techniques have been termed ‘metabolic FLIM’ and become the basis of clinical FLIM applications. Also, FLIM of virtually non‐fluorescent endogenous compounds, like carotenoids, lipofuscin, and melanin by high sensitivity and high‐resolution TCSPC FLIM may gain importance in future.

Due to the dependence of fluorescence lifetimes on physiological parameters, FLIM has made it into a number of clinical applications. Most of them are based on metabolic FLIM. Conditions such as inflammation, oxygen saturation, redox state and possible change from oxidative phosphorylation to glycolysis and cancer development are reflected in the amplitudes of the decay components of NAD(P)H and FAD. Applications include FLIM histology, cancer detection, personalised chemotherapy, selection of T cells, and inspection of oocytes and stem cells. Instruments for multiphoton tomography of skin, ophthalmic FLIM, FLIM‐guided surgery and fluorescence‐lifetime biopsy exist and are in the state of clinical trials or are even clinically approved.

Widespread introduction and approval of clinical FLIM techniques is currently impeded by a lack of cooperation between manufacturers of optical systems, clinical partners and manufacturers of clinical systems. The introduction of new methods and techniques into clinical use is extremely laborious and expensive and can only be achieved by a joint effort of all these partners.

## Conflicts of Interest

V. I. S.: Becker&Hickl GmbH (E), W. B.: Becker&Hickl GmbH (E). The rest of the authors declare no conflicts of interest.

## Data Availability

The data that support the findings of this study are available on request from the corresponding author. The data are not publicly available due to privacy or ethical restrictions.
